# The A-to-I editing of KPC1 promotes intrahepatic cholangiocarcinoma by attenuating proteasomal processing of NF-κB1 p105 to p50

**DOI:** 10.1186/s13046-022-02549-1

**Published:** 2022-12-08

**Authors:** Chengming Gao, Guangming Zhou, Jie Shi, Peipei Shi, Liang Jin, Yuanfeng Li, Xiaowen Wang, Song Liao, Han Yan, Junjie Wu, Yiming Lu, Yun Zhai, Jinxu Zhang, Haitao Zhang, Hongxing Zhang, Chenning Yang, Pengbo Cao, Shuqun Cheng, Gangqiao Zhou

**Affiliations:** 1 State Key Laboratory of Proteomics, National Center for Protein Sciences at Beijing, Beijing Institute of Radiation Medicine, 27 Taiping Road, Beijing, 100850 China; 2 Eastern Hepatobiliary Surgery Hospital, Navy Military Medical University, 225 Changhai Road, Shanghai, 200433 China; 3 Hebei University, Baoding City, China; 4 State Key Laboratory of Proteomics, National Center for Protein Sciences at Beijing, Beijing Institute of Lifeomics, Beijing, China; 5 Medical School of Chinese PLA, Beijing, China; 6 Anhui Medical University, Hefei City, China; 7 Collaborative Innovation Center for Personalized Cancer Medicine, Center for Global Health, School of Public Health, Nanjing Medical University, Nanjing City, China

**Keywords:** Intrahepatic cholangiocarcinoma, RNA editing, KPC1, p105, NF-κB pathway

## Abstract

**Background:**

Aberrant RNA editing of adenosine-to-inosine (A-to-I) has been linked to multiple human cancers, but its role in intrahepatic cholangiocarcinoma (iCCA) remains unknown. We conducted an exome-wide investigation to search for dysregulated RNA editing that drive iCCA pathogenesis.

**Methods:**

An integrative whole-exome and transcriptome sequencing analysis was performed to elucidate the RNA editing landscape in iCCAs. Putative RNA editing sites were validated by Sanger sequencing. *In vitro* and *in vivo* experiments were used to assess the effects of an exemplary target gene *Kip1 ubiquitination-promoting complex 1* (*KPC1*) and its editing on iCCA cells growth and metastasis. Crosstalk between *KPC1* RNA editing and NF-κB signaling was analyzed by molecular methods.

**Results:**

Through integrative omics analyses, we revealed an adenosine deaminases acting on RNA 1A (ADAR1)-mediated over-editing pattern in iCCAs. *ADAR1* is frequently amplified and overexpressed in iCCAs and plays oncogenic roles. Notably, we identified a novel ADAR1-mediated A-to-I editing of *KPC1* transcript, which results in substitution of methionine with valine at residue 8 (p.M8V). KPC1 p.M8V editing confers loss-of-function phenotypes through blunting the tumor-suppressive role of wild-type KPC1. Mechanistically, KPC1 p.M8V weakens the affinity of KPC1 to its substrate NF-κB1 p105, thereby reducing the ubiquitinating and proteasomal processing of p105 to p50, which in turn enhances the activity of oncogenic NF-κB signaling.

**Conclusions:**

Our findings established that amplification-driven *ADAR1* overexpression results in overediting of KPC1 p.M8V in iCCAs, leading to progression *via* activation of the NF-κB signaling pathway, and suggested ADAR1-KPC1-NF-κB axis as a potential therapeutic target for iCCA.

**Supplementary Information:**

The online version contains supplementary material available at 10.1186/s13046-022-02549-1.

## Background

The intrahepatic cholangiocarcinoma (iCCA) arises from the epithelial lining of the peripheral intrahepatic bile duct epithelium and is the second most common lethal primary liver cancer (PLC), accounting for 5% ~ 10% of all PLCs [1–3]. The worldwide incidence of this malignancy has been increasing steadily and substantially over the past four decades [1]. The prognosis of iCCA is devastating and the mortality rate is high [4]. Recently, the landscapes of genomic and epigenomic alterations in iCCA have been characterized. The spectrum of these alterations encompasses recurrent mutations (*e.g.*, in *IDH1/2*, *TP53*, *KRAS*, *ARID1A*, *FGFR1/2/3*, *EPHA2*, *PBRM1* and *BAP1*) [5], copy number alterations (*e.g.*, focal deletions of *CDKN2A* and amplifications of *MCL1*) [6], gene fusions (*e.g.*, *FGFR2* fusions) [5] and epigenetic deregulation (*e.g.*, *ARID1A, IDH1/2* and *BAP1*) [6, 7]. These studies revealed high genetic heterogeneity of iCCAs, which confound the effectiveness of medical treatment. Thus, there remains a compelling need to identify novel molecular targets and oncogenic processes operative in iCCA, which might be helpful for developing novel treatment strategies for this malignancy.

In addition to genomic alterations, RNA editing is an epigenetic mechanism in which sequence alterations are introduced into the genome-encoded RNA transcripts, while leaving the underlying genome intact and unmodified [8]. The most common type of RNA editing in humans involves adenosine-to-inosine (A-to-I) editing, which is catalyzed by the adenosine deaminase acting on RNA (ADAR) enzymes [9]. A-to-I editing has been shown to increase both transcript and proteome diversities, as inosine residues are recognized as guanosine by the cellular translational machinery. Due to the diverse impacts of RNA editing on gene expression and function, several dysregulated editing events have been asserted as pivotal determinants in progression of multiple human cancers, including myeloma, glioblastoma, and liver, gastric, colorectal and esophageal cancers [10–15]. To date, however, the roles of ADARs and the landscape of RNA-edited targets in iCCA transcriptome remain unknown.

In this study, we performed an integrative whole-exome and transcriptome sequencing analysis of iCCA tissues and their matched adjacent non-tumor liver tissues. We found evidence of widespread RNA misediting and ADAR1 overexpression in iCCA tissues relative to non-tumor liver tissues, and a significant correlation between higher ADAR1 expression and poor clinical outcomes of iCCA patients. Further, using an exemplary target gene *Kip1 ubiquitination-promoting complex 1* (*KPC1*), we identified an ADAR1-mediated recoding RNA editing event causing an amino acid substitution from methionine (Met) to valine (Val) at codon 8 of KPC1 (p.M8V), conferring a loss-of-function phenotype that neutralizes the tumor-suppressive ability of the unedited KPC1. These results highlighted that RNA editing might be another mechanism of sequence alteration contributing to iCCA progression.

## Methods

### Clinical samples

This study included a total of 194 pairs of iCCA tissues and matched non-tumor liver tissues derived from the patients of three cohorts, including one discovery cohort (designated as DISC cohort) and two validation cohorts (designated as VALI1 and VALI2, respectively), which were all recruited from the Eastern Hepatobiliary Surgery Hospital (Shanghai, China) (Supplementary Table 1). The diagnosis of iCCA was described in detail previously [16]. Briefly, the iCCA cases were newly diagnosed, previously untreated by chemotherapy or radiotherapy, pathologically confirmed, and proved not to have other cancers. Patients with gallbladder cancer or perihilar cholangiocarcinoma were excluded. All the patients underwent surgical resection and received no chemotherapy or radiotherapy before surgery. The RNAs extracted from the tissues of the patients from the DISC cohort (*n* = 15; recruited between August, 2008 and December, 2012) were used for RNA-seq, and the matched genomic DNAs were used for WES and genome-wide SNP genotyping by Affymetrix SNP Array 6.0, respectively. The genomic DNAs, RNAs and proteins from the VALI1 cohort (*n* = 26; recruited between August, 2008 and December, 2012) were used for RNA editing validation by Sanger sequencing, mRNA expression quantification by quantitative real-time PCR (qRT-PCR) assays and protein expression quantification by immunoblotting assays. The tissue microarrays from the iCCA patients of the VALI2 cohort (*n* = 137; recruited between July, 2001 and December, 2005) were used for IHC assays. Additionally, we collected 18 non-tumor bile duct tissues (NTBD) derived from cholangiocarcinoma patients (recruited between September, 2022 and November, 2022 from the Eastern Hepatobiliary Surgery Hospital [Shanghai, China]) for quantifying the protein levels of ADAR1/2 and *KPC1* RNA editing levels. Written informed consent was obtained from each patient, and the demographic and clinical data were collected by structured questionnaire.

### Identification of the putative RNA editing events

RNA-seq was performed at MyGenostics Inc. (Beijing, China). The libraries for RNA-seq were prepared using the Illumina Tru-Seq RNA Sample Preparation v2 Kit according to the manufacturer’s instructions, and then subjected to massively parallel sequencing using Illumina HiSeq3000. The TopHat (v2.0.10) [17] was used to align the reads to the NCBI human reference genome assembly (build 36.1) and the UCSC annotated genes with default parameters. Genomic DNA was captured using the SureSelect Human All Exon V5 + UTRs kit (Agilen, USA). Captured DNAs were then subjected to massively parallel sequencing using Illumina HiSeq2000. WES was performed by CapitalBio Corp. (Beijing, China). The sequencing reads were aligned to the NCBI human reference genome assembly (build 36.1) by BWA (v0.5.9) with default parameters.

To identify putative RNA editing events, we first detected the variants in RNA-seq data by VarScan (v2.2.5) [18]. The calling of variants was constrained to those locations within gene regions with at least 8 × , variation frequency of ≥ 5%, no strand bias, and base quality ≥ 20. Then, we further removed the somatic SNVs obtained by WES and known SNPs (including SNPs with MAF > 0.001 in dbSNP [database version 135] or the 1000 Genomes Project [February 2012 data release]). Next, we discarded those variants in simple repeats according to RepeatMasker annotation. Further, all the variants were separated into Alu and non-Alu groups. The mismatches in Alu elements showed a convincingly high fraction of A-to-G mismatches and did not receive further stringent filtering. The variants in non-Alu regions were subjected to further refinement according to more stringent criteria: variation frequency of ≥ 10%; not located in regions of high similarity to the other parts of the genome determined by BLAT as previously reported [19]. The conservations of these putative RNA editing sites were predicted using the PhyloP [20].

### Estimation of RNA editing levels by Sanger sequencing

First, we performed semi-quantitative PCR using the following protocol: typically, ~ 50 ng of gDNA or ~ 10 ng of cDNA template were prepared in a 25 μL PCR reaction assembled with 1 × SYBR Green Supermix (Bio-Rad), and 200 nM of the forward and reverse primers, and then were subjected to PCR program as previously described [19]. The primers of candidate editing sites used in this study are shown in Supplementary Table 8. Finally, the PCR products were subjected to Sanger sequencing by BGI Genomics Co Ltd (Shenzhen City, China), and the percentage of RNA editing levels were estimated from Sanger sequencing using QSVanalyzer [21]. Among the 29 putative A-to-I editing sites in coding regions, 25 ones were replicated by Sanger sequencing, including 14 non-synonymous sites and 11 synonymous sites.

### Cell lines

The human intrahepatic biliary epithelial cell line HIBEpiC, human embryonic kidney cell line HEK293T, and cholangiocarcinoma cell lines QBC939, TFK-1 and HuCC-T were maintained in high-glucose Dulbecco’s modified Eagle’s medium (DMEM; HyClone, USA), and the human cholangiocarcinoma cell lines HCCC9810, RBE and Huh28 were maintained in Roswell Park Memorial Institute-1640 medium (RPMI-1640; Invitrogen, USA), supplemented with 10% fetal bovine serum (FBS; Gibco, Australia), 100 U/mL penicillin, and 100 μg/mL streptomycin. The HIBEpiC, QBC939, HuCC-T1 and TFK-1 cell lines were provided by Prof. Lianxin Liu (The First Affiliated Hospital, The University of Sciences and Technology of China, Hefei City, China); The HCCC9810, RBE and Huh28 cell lines were provided by Prof. Xianghuo He (Shanghai Cancer Institute, Renji Hospital, Shanghai, China). The cells were incubated at 37 °C in a humidified incubator containing 5% CO_2_. All the cell lines used in this study were regularly authenticated by morphological observation and tested for mycoplasma contamination.

### Immunoblotting assays

For protein analysis of whole-cell lysates, cells were lysed in RIPA (CWBIO, China) buffer plus EDTA-free protease inhibitor cocktail (04,693,132,001; Roche) for 30 min in ice. Cell lysates were then collected after centrifugation at 12,000 rpm for 15 min at 4 °C. Total proteins (10—20 μg) were re-suspended in Laemmli buffer (63 mM Tris–HCl, 10% glycerol, 2% SDS, 0.0025% bromophenolblue, pH 6.8) and electrophoresed on 10% SDS–polyacrylamide gels. Then, the proteins were transferred to a Pure Nitrocellulose Blotting membrane (Life Science, Mexico) by 200 mA, 2 h (h), and 4 °C. The membrane was blocked with 5% skim milk for 1 h before incubated with the indicated primary antibodies at 4 °C overnight, and anti-mouse or anti-rabbit secondary antibodies for 1 h at room temperature conjugated to horseradish peroxidase (HRP). Finally, the immunoreactive bands were detected using the SuperSignal™ West Pico chemiluminescent substrate kit (Thermo Fisher Scientific, USA) and Western blotting detection system (Bio-Rad, USA). The following antibodies were used in this study: anti-KPC1 (1:500; H00063891-M01, NovusBio, USA), anti-ADAR1 (1:1,000; ab88574, Abcam, UK), anti-ADAR2 (1:1,000; F6011, Sigma, USA), anti-p65 (1:1,000; No.21014–2, Signalway Antibody, USA), anti-p-p65 (1:1,000; ser536; No.11014–2,, Signalway Antibody, USA), anti-tubulin (1:2,000; 66,240–1-Ig, Proteintech, USA), anti-GAPDH (1:2,000; 60,004–1-Ig, Proteintech, USA), anti-Myc (1:1,000; 16,286–1-AP, Proteintech, USA), anti-Flag (1:1,000; 66,008–2-Ig, Proteintech, USA), anti-H3 (1:1,000; 17,168–1-AP, Proteintech, USA), anti-p27 (1:500; 25,614–1-AP, Proteintech, USA), anti-HA (1:1,000; M180-3, MBL, Japan), anti-NF-κB1 p105/p50 (1:1,000; No.3035, CST, USA), anti-E-cadherin (1:1,000; No.14472, CST, USA), anti-N-cadherin (1:1,000; No.13116, CST, USA), anti-Vimentin (1:1,000; No.5741, CST, USA), HRP-conjugated goat anti-mouse IgG (1:3,000; CW0102M, CWBIO, China) and HRP-conjugated goat anti-rabbit secondary antibody (1:3,000; CW0103M, CWBIO, China).

### KPC1 or ADAR1 knockdown and overexpression assays

Lentiviral vector construction, viral packaging and infection were performed as previously described. Briefly, two specific shRNAs targeting the RNA sequences of *KPC1* or *ADAR1* (Supplementary Table 8) were designed and synthesized by Inovogen (Beijing, China). These shRNAs were cloned into the pLVshRNA-EGFP(2A)-Puro Lentiviral vector system (Inovogen, China) and confirmed by Sanger sequencing. A non-targeting scrambled shRNA which had no known homology with any mammalian gene was designed and used as the non-specific control. After 24 h, the infected cells were selected with puromycin (2 μg/mL; AXIO0053, Sigma, USA) for 2 weeks before evaluation for knockdown efficiency.

The pLV-EGFP(2A)Puro lentiviral vector was used for *KPC1* overexpression and the pLV-neo lentiviral vector was used for *ADAR1* overexpression. The constructs were confirmed by DNA sequencing. The vectors with shRNAs or the intact sequence of *KPC1* and *ADAR1* along with viral packaging plasmids (pMD2.G and psPAX2) were transfected into human embryonic kidney 293 T cells using Lipofectamine 2000. Virus supernatant was harvested after 48 h, filtered through a 0.45 μM filter, and incubated on target cells for 6 h at a 1:10 dilution with 10 μg/mL polybrene (107,689, Sigma, USA). Infected cells were selected with 2 μg/mL puromycin or 500 ng/mL G418 (A1720, Sigma, USA) for 2 weeks before evaluation for overexpression efficiency.

### Cells growth, colony formation, migration and invasion assays

The growth curve of iCCA cells were measured using Cell Counting Kit-8 (CCK-8) (Dojindo, Japan) according to the manufacturer’s instructions. For cells colony formation assays, cells were seeded at a density of 2,000 ~ 3,000 cells in one 6-cm culture dish and cultured for 2 weeks; the colonies were stained with 1% crystal violet and counted. For cells migration assays, 5 × 10^4^ cells (200 μL) were planted on the top chamber of each insert (BD Biosciences, USA) with 8 μm-diameter pores on its membrane. Six hundred μL DMEM supplemented with 20% FBS was injected into the lower chambers. After incubation at 37 °C for 24 ~ 36 h, the cells adhering to the lower side of the inserts were stained with 0.1% crystal violet solution, and then imaged and counted by IX71 inverted microscope (Olympus, Japan). For cells invasion assays, all the experimental protocols are the same as the migration assays except the special chamber used (354,480, CORBING, USA) and longer incubation (48 ~ 72 h). All the experiments were repeated independently at least three times.

### Nude mice assays

The animal studies were approved by the Medical Ethical Committee of Beijing Institute of Radiation Medicine (Beijing, China). Male athymic BALB/c nude mice (6-weeks old) were purchased from Charles River (Beijing, China). For tumorigenicity assays, the exponentially growing RBE cells were stably transfected with an empty vector (pLV-EGFP-2A) or with vectors coding for pLV-EGFP-2A-KPC1-WT/EDT. Cells were dissociated with trypsin and washed with PBS. Cell suspension (3 × 10^6^ cells) was inoculated subcutaneously at the left flank of nude mice (*n* = 9). Xenograft size was determined every four days by externally measuring the growing tumors in two dimensions using a caliper. Tumor volume (V) was determined by the equation V = L × W^2^ × 0.5, where L and W are the length and width of the xenograft, respectively. At the end of the experiment, mice were sacrificed and xenografts were harvested, weighed and fixed in formalin. Paraffin-embedded sections were stained with Ki-67 and p105/p50.

For metastasis assays, RBE cells bearing firefly luciferase were stably transfected with an empty vector (pLV-EGFP-2A) or with vectors coding for pLV-EGFP-2A-KPC1-WT/EDT. Cells were dissociated with trypsin and washed with PBS. These cells (4 × 10^4^) were intravenously injected into tail veins of 6-weeks old BALB/C nude mice (*n* = 6). Lung metastatic signals were detected using the IVIS system (Xenogen, USA). The mice were sacrificed at 37 days after injection, and the lungs were harvested and fixed in 4% paraformaldehyde. The detectable tumor nodules on the surface of whole lung were counted for the metastatic index.

### Co-immuoprecipitation (Co-IP) assays

QBC939 or RBE cells were transfected with 2.0 μg of the Myc-tagged wild-type or edited KCP1 plasmid (Myc-KPC1-WT or Myc-KPC1-EDT) and 1.0 μg Flag-tagged p105 plasmid. Transfected cells were harvested and lysed in HEPES lysis buffer (20 mM HEPES, pH 7.2, 50 mM NaCl, 0.5% Triton X-100, 1 mM NaF, 1 mM dithiothreitol) for 30 min in ice and boiled with 2 × SDS-PAGE loading buffer for 10 min. For immunoprecipitation, cell lysates were prepared in 500 mL HEPES buffer supplemented with protease inhibitor cocktail (Roche) and phosphatase inhibitors (10 mM NaF and 1 mM Na_3_VO_4_). Immunoprecipitations were performed using mouse anti-Myc (2 mg), anti-Flag (2.5 mg) monoclonal antibodies for 4 h at 4 °C followed by incubation with protein A/G-agarose beads (Santa Cruz, USA) overnight at 4 °C. The beads were then washed three times in HEPES buffer. The proteins were released from the beads by boiling in SDS-PAGE sample buffer and analyzed by immunoblotting with indicated antibodies.

### Ubiquitination assays

For p105 ubiquitination assay, HA-ubiquitin, Myc-KPC1-WT/EDT, Flag-p105 were transiently transfected into the QBC939 or RBE cells with Lipofectamine 2000. Twenty-four hours after transfection, the cells were treated with 20 μM of proteasome inhibitor MG132 (133,407–82-6, Sigma, USA) for 8 h. The cells were washed with PBS, pelleted, and lysed in HEPES buffer (20 mM HEPES, pH 7.2, 50 mM NaCl, 1 mM NaF, 0.5% Triton X-100) plus 0.1% SDS, 20 μM MG132 and protease-inhibitor cocktail (Roche, Germany). The lysates were centrifuged to obtain cytosolic proteins and incubated with anti-Flag antibody for 3 h and protein A/G agarose beads (Santa Cruz, USA) for a further 8 h at 4 ℃. Then the beads were washed for three times with HEPES buffer. The proteins were released from the beads by boiling in SDS-PAGE sample buffer and analyzed by immunoblotting with indicated antibodies.

### Luciferase reporter assays for NF-κB pathway

The luciferase reporter gene plasmids for NF-κB were obtained from Promega (E3292, USA). For NF-κB luciferase reporter assays, the reporter plasmids and pRL-TK plasmids were co-transfected into QBC939 or RBE cells by Lipofectamine 2000 (Invitrogen, USA) in 24-well plates. Forty-eight hours after transfection, cells were harvested and analyzed using Dual-Luciferase® Reporter Assay System (Promega, USA). Firefly activity was normalized to Renilla luciferase activity. All the experiments were repeated independently at least three times.

### Chromatin immunoprecipitation (ChIP) assays

The experiments were performed on freshly obtained cell lysates from QBC939 or RBE cells by using the ChIP Assay Kit (17–295, Millipore, USA) according to the manufacturer’s instructions. First, cells were harvested after fixation with 1% formaldehyde. After cell lysis and sonication, the fragmented DNA was diluted in ChIP dilution buffer and pre-adsorbed with protein A agarose/salmon sperm DNA (16-157C, Millipore, USA) for 1 h at 4 °C. Then 5% of the chromatin was removed and saved as input. It was then incubated overnight at 4 °C with 3–5 μg of the antibody against NF-κB p50 (1:50; No.13586, CST, USA) or normal rabbit IgG (PP4B, Millipore, USA). Antibody-chromatin complexes were captured by incubation with protein A sepharose and centrifuged. Reversal of protein cross-linking and proteinase K digestion, followed by purification of the DNA, was then achieved. The binding abilities of p50 to the promoters of its targets (*IL6*, *IL6R*, *MMP9* and *VEGFA*) were measured by quantitative PCR with the iQ5™ Optical Module (BIO-RAD, USA) System using SYBR Green PCR Kit (TaKaRa, Japan). The primers used for ChIP-qPCR were provided in the Supplementary Table 8.

### Statistical analyses

The mouse studies were randomized. Student’s *t* test was used to compare two groups of quantitative data assuming equal variances. If measured values did not meet the assumptions of normality and homogeneity of variances, log-transformation was then used before the *t*-tests were performed. Fisher’s exact test or *χ*^2^ test was used for the analyses of contingency tables depending on the sample sizes. Survival curves were calculated using the Kaplan–Meier method, and the significance was determined by log-rank test. *P* < 0.05 was considered to be statistically significant. All the statistical analyses, except where otherwise noted, were performed using R version 3.3.0 software (www.rproject.org) or GraphPad Prism (version 6).

## Results

### Identification of exome-wide protein-coding A-to-I RNA editing in human iCCAs

**Fig. 1:**
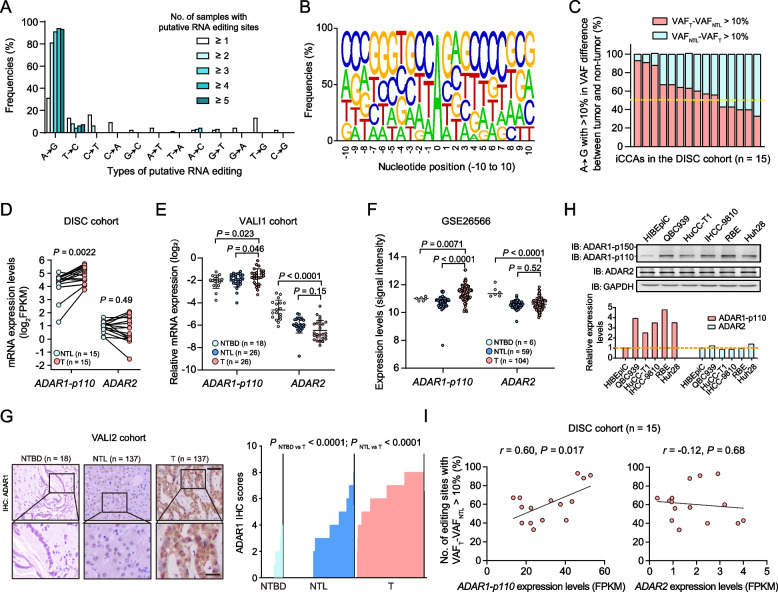
RNA over-editing pattern in protein-coding regions of iCCA genomes is mediated by ADAR1.** a** The distribution of 12 types of putative protein-coding RNA editing event. **b** Neighbor preferences (± 10 nucleotide [nt]) of the A-to-I RNA editing sites. A, adenosine; T, thymidine; C, cytosine; G, guanosine; I, inosine. **c** The percentages of the A-to-I editing sites (present in ≥ 2 samples) with > 10% increase or decrease in variation frequency (VAF) between the iCCA tissues (T) and non-tumor liver tissues (NTL) from the discovery cohort (DISC, *n* = 15). **d-e** The mRNA expression levels of *ADAR1-p110* and *ADAR2* in NTL and T from the patients of DISC cohort (*n* = 15) assessed by RNA-seq. FPKM, fragments per kilobase of exon model per million reads mapped. **e** and **f** The mRNA expression levels of *ADAR1-p110* and *ADAR2* in non-tumor bile duct tissues (NTBD), NTL and T from the patients of VALI1 cohort assessed by qRT-PCR assays (**E**), and the patients of Andersen’s cohort (GSE26566) assessed by beadchip (**F**). **g** Immunohistochemistry (IHC) analyses of ADAR1 protein levels in NTBD, NTL and T from the patients of VALI2 cohort. Scale bars: top, 300 μm; bottom, 100 μm. The *P* value was assessed by Wilcox rank sum test. **h** Immunoblotting assays for ADAR1 and ADAR2 in a panel of cell lines, consisting of one normal intrahepatic biliary epithelial cell line (HIBEpiC) and five types of human iCCA cell line (including IHCC-9810, QBC939, HuCC-T1, RBE and Huh28). **i** Correlations (Spearman’s rank correlation rho) between the percentages of A-to-I over-editing sites (VAF_T_-VAF_NTL_ > 10%; present in ≥ 2 samples) and the mRNA expression levels of *ADAR1-p110* (left) or *ADAR2* (right) in iCCA tissues from the patients of DISC cohort

We performed RNA sequencing (RNA-seq) and whole-exome sequencing (WES) in tumor tissues and paired adjacent non-tumor liver tissues from 15 patients with iCCA (designated as discovery cohort [DISC]; Supplementary Tables 1–3 and Supplementary Fig. 1A-C). To identify the RNA editing events, we first detected the putative RNA variants in the RNA-seq data and DNA variants in the WES data, respectively, by use of VarScan [18]. Then, we filtered out the somatic mutations or potential contamination of germline single nucleotide polymorphisms (SNPs) from the putative DNA and RNA variants. We also applied a more strict criteria to filter out the false positives located outside the Alu elements as previously described [19] (Methods and Supplementary Methods; Supplementary Fig. S1C). Finally, we obtained a total of 409 putative protein-coding RNA editing sites, and among which 91.2% ones (373/409) were singletons present in one sample. When only considered the 36 sites that were present in at least two samples, the frequency of A-to-guanosine (G) events achieved 80.1% (29/36) (Fig. 1a and Supplementary Fig. 1D), consistent with the findings that the A-to-I editing (recognized as A-to-G) is the most common type of RNA editing events in humans [19, 22]. As expected, after removal of singletons, the frequency of the putative RNA editing sites annotated in the publicly available databases of A-to-I editing resources DARNED [23] or RADAR [24] increased from 15.4% (63/409) to 66.7% (24/36) (Supplementary Fig. 1E). Among the 29 putative A-to-I editing sites present in at least two samples, 58.6% (17/29) were located within Alu repetitive elements (Supplementary Fig. 1F), which is in accordance with their ability to form double-stranded structures, a prerequisite for RNA editing [22]. We next performed technical validation analyses for these 29 putative A-to-I editing events by Sanger sequencing of cDNAs and genomic DNAs from the tissues of individuals in DISC cohort, and 25 ones survived (Supplementary Table 4 and Supplementary Fig. 1G). The local sequence motif analyses of these validated A-to-I editing sites showed that CAG and TAG sites predominate the most frequently edited sequence context in A-to-I editing sites (Fig. 1b), as previously reported [25].

### iCCA displays an ADAR1-mediated RNA over-editing pattern

By comparing the frequencies of the significantly altered A-to-I editing events between iCCA tissues and paired adjacent non-tumor liver tissues (defined as absolute difference of editing levels |VAF_tumor_ – VAF_non-tumor_|> 10%) among the 25 A-to-I editing sites for each patient in the DISC cohort, we observed that 66.7% (10/15) patients harbor more over-edited events than under-edited events in iCCA tissues (Fig. 1c), therefore indicating an RNA over-editing pattern for this malignancy.

Then, to explore the molecular determinants underlying this over-editing pattern in iCCA tissues, we examined the associations between the magnitude of over-editing and the expression levels of ADAR1 and ADAR2 in iCCAs, which are the major enzymes mediating A-to-I RNA editing in humans [26]. ADAR1 has two major isoforms: a shorter 110-kDa isoform (p110) and a longer interferon-inducible 150-kDa isoform (p150). ADAR1-p110 isoform and ADAR2 are ubiquitously expressed in humans and exhibit catalytic activities towards host nuclear pre-RNAs, while ADAR1-p150 isoform acts primarily on exogenous viral RNAs [27]. Thus, we first checked the abundance of *ADAR1-p110* and *ADAR2* in the RNA-seq data from the DISC cohort. The abundance of *ADAR1-p110* (~ 40 fragments per kilobase of exon model per million reads mapped [FPKM]) was shown to be 20-fold higher than that of *ADAR2* (~ 2 FPKM) (Fig. 1d). Moreover, *ADAR1-p110* showed an average of 1.8-fold greater expression levels in iCCA tissues compared to the non-tumor liver tissues (*P* = 0.0022), whereas *ADAR2* had no significant change (Fig. 1d). We further examined the ADAR1 expression levels in the iCCAs from the validation cohort 1 (VALI1; *n* = 26) by qRT-PCR assays, the Andersen’s cohort [28] by beadchip (GSE26566), and the VALI2 (*n* = 137) by immunohistochemistry (IHC) assays, respectively. Again, the results revealed significant up-regulation of ADAR1 mRNA (*P* = 0.032) and protein levels (*P* = 1.13 × 10^–39^), respectively, in iCCA tissues compared to the non-tumor liver tissues or non-tumor bile duct tissues (Fig. 1e-g). Accordingly, compared to the HIBEpiC cell line (normal intrahepatic biliary epithelial cells), five types of cholangiocarcinoma cell line (IHCC-9810, QBC939, HuCC-T1, RBE and Huh28) showed globally higher ADAR1 protein levels (Fig. 1h). Consistently, ADAR2 levels showed no significant change in both cholangiocarcinoma tissues and cell lines (Fig. 1e and g).

Next, we examined the correlation between the expression levels of ADARs and the magnitude of RNA over-editing in tissues of the patients from the DISC cohort. The increased percentage of A-to-I over-editing sites is significantly correlated with the increased expression of *ADAR1-p110* (*r* = 0.60, *P* = 0.017; Fig. 1i), but not with that of *ADAR2* (*r* = -0.12, *P* = 0.68; Fig. 1i), consistent with the findings from previous studies that the globally altered RNA editing patterns in tumors are more likely to be affected by ADAR1 rather than by the other editing enzymes [29]. Taken together, these observations suggested that the A-to-I over-editing occurs frequently in iCCAs and might be dominated by ADAR1.

### ADAR1 is frequently amplified and confers oncogenic role in iCCAs

**Fig. 2:**
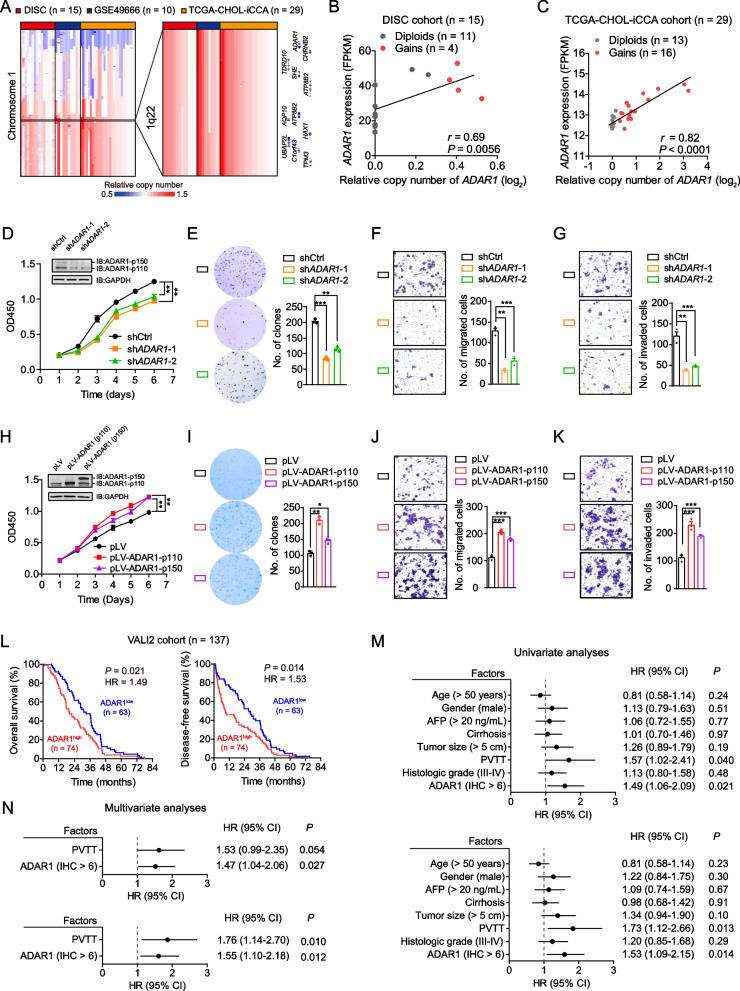
***ADAR1*** is frequently amplified in iCCA tissues and confers oncogenic roles in iCCA. a Somatic genomic amplifications of *ADAR1* in iCCAs from the patients of DISC cohort (*n* = 15), a previous study (GSE49666; *n* = 10) and the TCGA-CHOL cohort (*n* = 29), respectively. DISC, discovery; TCGA-CHOL, the Cancer Genome Atlas-cholangiocarcinoma. **b** and **c** Correlations between the relative copy number of *ADAR1* and its mRNA expression levels in iCCA tissues from the patients of DISC (**B**) and TCGA-CHOL (**C**) cohorts, which were assessed by Spearman’s rank correlation analyses. **d-g** Knockdown of *ADAR1* significantly reduced QBC939 cells growth which was assessed by CCK-8 assays (**D**), plate cell colony formation (**E**), migration (**F**) and invasion (**G**). **h–k** Overexpression of *ADAR1-p110* or -*p150* isoform significantly promoted QBC939 cells growth (**H**), plate cell colony formation (**I**), migration (**J**) and invasion (**K**). **l** Kaplan–Meier analyses showed that higher ADAR1 protein levels predict decreased overall survival (OS) and disease-free survival (DFS) rates of iCCA patients from the VALI2 cohort. ADAR1 protein levels were determined by IHC assays, and the IHC scores > 6 were designated as “high”; whereas the others were “low”. The significance was determined by log-rank test. **m** and **N** Univariate (**M**) and multivariate (**N**) Cox hazard ratios analyses for OS (Up panel) and DFS (Down panel) rates in iCCA patients from the VALI2 cohort. HR, hazard ratio; CI, confidence interval; AFP, alpha-fetoprotein; PVTT, portal vein tumor thrombus. In (**D-K)**, data are presented as the mean ± standard deviation (s.d.). ^*^P < 0.05, ^**^P < 0.01 and ^***^P < 0.001; assessed by Student’s *t* test

Next, we sought to dissect the underlying mechanism of ADAR1 overexpression in iCCA tissues. The WES data from the DISC cohort revealed no somatic mutations within *ADAR1* (data not shown). However, the copy number alteration (CNA) analyses based on the SNP array data from the DISC cohort (*n* = 15), a previous study (GSE49666 from the Gene Expression Omnibus [GEO] database; *n* = 10) [30] and the iCCAs in the Cancer Genome Atlas-cholangiocarcinoma cohort (TCGA-CHOL-iCCA; *n* = 29) revealed that the *ADAR1* locus at chromosome 1q22 is subjected to significant genomic gains, with a frequency of 27% ~ 55% in iCCA patients (Fig. 2a). Pan-cancer analyses on the basis of the TCGA datasets revealed that *ADAR1* is also frequently amplified in several other types of cancer, including the liver hepatocellular carcinoma (LIHC), breast invasive carcinoma (BRCA), uterine carcinosarcoma (UCS) and ovarian cancer (OV) (Supplementary Fig. 2A). Further, we observed a significant correlation between the *ADAR1* copy number and its elevated mRNA expression levels in either iCCAs or several other types of cancer (Fig. 2b and  c, and Supplementary Fig. 2B and C; the expression fold change [tumor/non-tumor] in TCGA-CHOL-iCCA ranked the 1st). Finally, the epigenomic analysis of DNA methylation levels at *ADAR1* promoter in the dataset from Roessler’s cohort (GSE201241) [31] and TCGA-CHOL-iCCA cohort showed no significant difference between the cholangiocarcinoma tissues and non-tumor liver tissues or non-tumor bile duct tissues (Supplementary Fig. 2D and E). Collectively, these data implicated that the genomic gain-driven overexpression of *ADAR1* might be a common event in iCCAs.

Next, we assessed the potential functional effects of ADAR1 on iCCA cells. We observed that knockdown of *ADAR1* in iCCA cell lines QBC939 and RBE significantly reduces the capabilities of cells growth, colony formation, migration and invasion (Fig. 2d-g, and Supplementary Fig. 3A-D). Conversely, overexpression of ADAR1 in QBC939 and RBE cells significantly promoted the abilities of cells growth, colony formation, migration and invasion (Fig. 2h-k, and Supplementary Fig. 3E-H). Further, we investigated whether the expression levels of ADAR1 are correlated with the clinical outcomes of iCCA patients. No significant association between the ADAR1 expression and the pathological parameters was observed in iCCAs from the VALI2 cohort (Supplementary Table 5). However, Kaplan–Meier analyses revealed that higher ADAR1 expression predicts decreased overall survival (OS; *P* = 0.021) and disease-free survival (DFS; *P* = 0.014) (Fig. 2l). Multivariable Cox proportional hazards regression analyses further revealed that the higher ADAR1 expression in iCCA tissues is an independent factor for the decreased OS (*P* = 0.027) and DFS rates (*P* = 0.012) (Supplementary Table 6 and Fig. 2m and n). Collectively, these findings supported that the genomic gain-driven overexpression of ADAR1 plays an oncogenic role in the development of iCCA.

### A22G at KPC1 transcript is over-edited in iCCA tissues and is mediated by ADAR1-p110

**Fig. 3:**
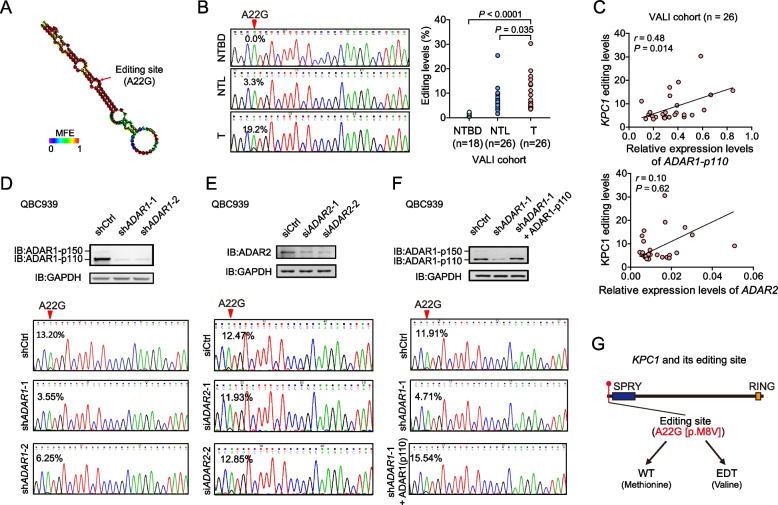
A22G at KPC1 transcript is over-edited in iCCA tissues and is mediated by ADAR1-p110.** a** Vienna prediction of RNA secondary structure of the sequences surrounding the editing site A22G at *KPC1* transcript. MFE, minimum free energy. **b** Editing levels of *KPC1* A22G in non-tumor bile duct tissues (NTBD), non-tumor liver tissues (NTL) and iCCA tissues (T) from the patients of VALI1 cohort. Left panel, the representative sequence chromatograms surrounding the editing site A22G at *KPC1* transcript. The *P* value was assessed by paired Student’s *t* test. **c** Correlations between the *KPC1* A22G editing levels and the mRNA expression levels of *ADAR1-p110* or *ADAR2*, respectively, in iCCA tissues from the patients of VALI1 cohort. The correlations were assessed by Spearman's rank correlation analyses. **d** and **e** The sequence chromatograms surrounding the editing site A22G at *KPC1* transcript in QBC939 cells upon knockdown of *ADAR1* by shRNAs (**D**) or knockdown of *ADAR2* by siRNAs (**E**). **f** The sequence chromatograms surrounding the editing site A22G at *KPC1* transcript in *ADAR1*-depleted (by sh*ADAR1*-1) QBC939 cells with or without re-expression of ADAR1-p110. **g** Schematic representation of the editing site A22G (p.M8V) at *KPC1* (NCBI No. NM_022064). SPRY, spla/ryanodine receptor domain; RING, really interesting new gene domain; WT, wide-type; EDT, edited

Among the fourteen A-to-I non-synonymous editing events (Supplementary Table 4), three ones have been experimentally dissected as pivotal modulators involving in tumorigenesis, including A1099G at *AZIN1* in HCC [12], A725G at *NEIL1* in glioblastoma [32] and A722G at *PODXL* in gastric cancer [15]. Additionally, other seven A-to-I non-synonymous editing sites have been shown to be significantly associated with certain type of cancer, but has not been functionally characterized, including A192G in *SRP9*, A6877G in *FLNB*, A1903G in *COG3*, A1096G in *ZNF669*, A712G and A646G in *METTL10* and A175G in *PPIL3* [29, 33–35]. Next, we focused on those events that reside within conserved regions (with PhyloP score > 1.0) and the tumorigenic functions of which remain unknown (Supplementary Table 4). This analysis revealed the top-ranking editing event to be A22G in *KPC1* transcript. We then performed TA cloning assays in QBC939 cells and purified the PCR products containing the *KPC1* A22G editing site, and confirmed that the A22G editing is not a sequencing error by Sanger sequencing (Supplementary Fig. 4A). Prediction of the secondary structure of the RNA sequences surrounding the A22G showed that this editing site is in a double-stranded RNA (dsRNA) stem loop, which might facilitate the binding of ADARs to the *KPC1* transcripts (Fig. 3a). By genotyping of A22G using Sanger sequencing in iCCA patients from the VALI1 cohort (Supplementary Table 1), we observed that the editing levels of this site significantly increase in iCCA tissues compared to the non-tumor liver tissues and non-tumor bile duct tissues (Fig. 3b). Consistently, over-editing levels of *KPC1* A22G were also observed in human cholangiocarcinoma cell lines compared to the normal intrahepatic biliary epithelial cell line HIBEpiC (Supplementary Fig. 4B). Together, these data suggested a close link between the over-editing site A22G in *KPC1* transcript and iCCA progression.

We then assessed the effects of ADARs on *KPC1* A22G editing levels in iCCAs. In iCCA tissues from the patients of VALI1 cohort, the higher editing levels of *KPC1* A22G were significantly correlated with increased mRNA levels of *ADAR1-p110* isoform (*r* = 0.48, *P* = 0.014), but not with that of *ADAR2* (*r* = 0.10, *P* = 0.62) (Fig. 3c). Further, we examined the abilities of ADAR1 or ADAR2 to catalyze the deamination of adenosine in *KPC1* transcripts in iCCA cell lines. Knockdown of *ADAR1* in QBE939 cells significantly reduced the *KPC1* A22G editing levels; whereas *ADAR2* knockdown had no this effect (Fig. 3d and e). Consistently, overexpression of ADAR1-p110 led to a significant increase in *KPC1* A22G editing levels, while ADAR1-p150 or ADAR2 did not (Supplementary Fig. 4C and D). Finally, the inhibitory effect of *ADAR1* knockdown on *KPC1* A22G editing levels was rescued by re-expressing of *ADAR1-p110*, but not -*p150* (Fig. 3f and Supplementary Fig. 4E). We got similar results in RBE cells (Supplementary Fig. 4F-H). Collectively, these results supported that the A22G over-editing of *KPC1* mRNA is specifically catalyzed by the ADAR1-p110 isoform.

### KPC1 inhibits iCCA tumorigenesis and its p.M8V editing displays loss-of-function effects

**Fig. 4:**
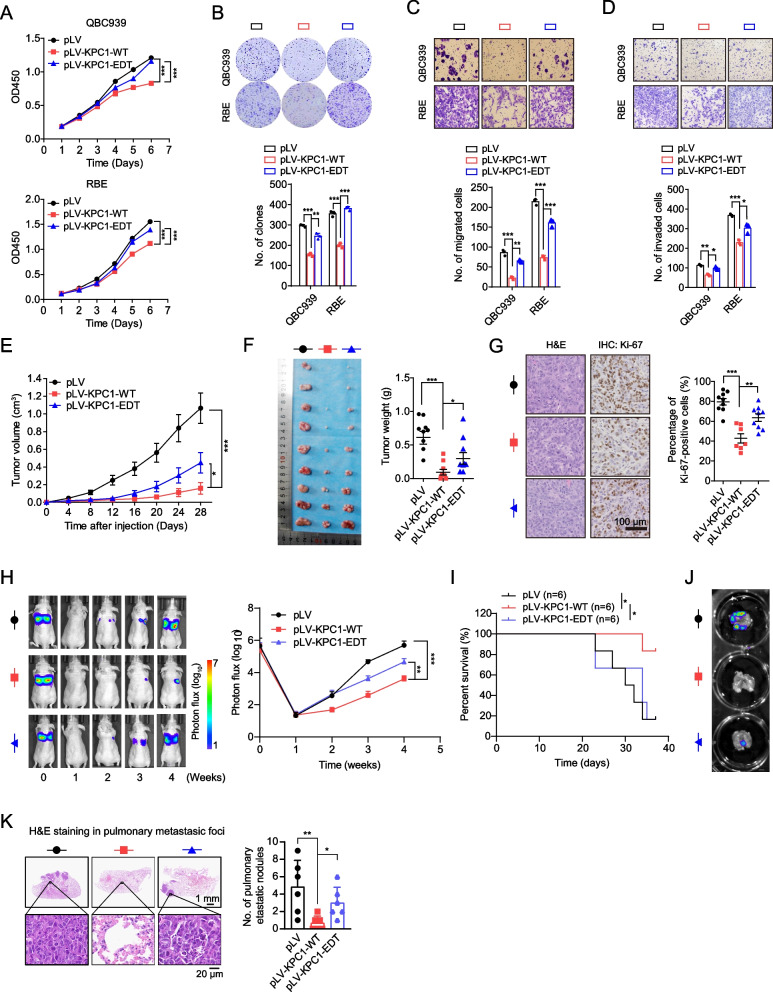
KPC1 p.M8V editing confers loss-of-function effects on the tumor suppressive roles of wide-type KPC1 in iCCAs.** a-d** The effects of overexpression of wide-type KPC1 (KPC1-WT) or edited KPC1 at p.M8V (A22G) (KPC1-EDT) on cells growth (**A**), plate colony formation (**B**), migration (**C**) and invasion (**D**) in QBC939 or RBE cells. **e** The effect of overexpression of KPC1-WT or KPC1-EDT on subcutaneous tumor growth (assessed by tumor volume) in nude mice. RBE cells with empty vector, overexpression of KPC1-WT or KPC1-EDT were implanted subcutaneously at the back of the mice (*n* = 9 mice/group). **f** Weights of the xenografted tumors at the 28th day after subcutaneous implantation at the back of the mice from the indicated groups. **g** The effect of overexpression of KPC1-WT or KPC1-EDT on tumor cells proliferation activities in nude mice, which was indicated by immunohistochemistry (IHC) staining for Ki-67. Left panel, the representative hematoxylin–eosin (H&E) and Ki-67 IHC staining images for the xenografted tumors. **h–k** The effect of overexpression of KPC1-WT or KPC1-EDT on tumor metastasis in nude mice. The empty vector-, KPC1-WT- or KPC1-EDT-overexpressed RBE cells (each also expressing luciferase) were injected via the tail vein into the nude mice (*n* = 6 mice/group). (**H)** Representative bioluminescence (BLI) images of the mice injected with RBE cells via the tail veins. (**I)** Kaplan–Meier plot of mice. The *P* value was assessed by log-rank test. (**J)** Representative ex vivo BLI images of metastases in lungs. (**K)** H&E staining of metastases in lungs. Scale bar, 100 µm. Data are presented as the mean ± standard deviation (s.d.). ^*^P < 0.05, ^**^P < 0.01 and ^***^P < 0.001; assessed by Student’s *t* test

*KPC1* encodes a catalytic subunit of the KPC complex, which displays E3 ubiquitin ligase activity. KPC1 has been shown recently to be a promising tumor suppressor in multiple human cancers [20, 36]. However, the relevance of KPC1 in the development of iCCA remains to be illustrated. The editing event A22G occurs at the residue 8 of KPC1, leading to a non-synonymous conversion from methionine to valine (p.M8V) (Fig. 3g), which may cause a deleterious effect predicted by PhyloP. Thus, we next assessed the effects of KPC1 and its recoding editing p.M8V on iCCA cells. Compared with the HIBEpiC cell line, most of the cholangiocarcinoma cell lines (including QBC939, IHCC-9810 and RBE cells) showed lower KPC1 expression levels (Supplementary Fig. 5A). Consistently, the decreased expression levels of KPC1 were also observed in iCCA tumors compared to non-tumor liver tissues and non-tumor bile duct tissues (Supplementary Fig. 5B). We observed that knockdown of *KPC1* promotes the abilities of growth, migration and invasion in RBE and IHCC-9810 cells (Supplementary Fig. 5C and D). To determine the effects of KPC1 p.M8V, we transfected the wide-type (KPC-WT) or p.M8V-edited KPC1 (KPC1-EDT) expression construct into the QBC939 and RBE cells. We observed that overexpression of KPC1-WT significantly reduces the cells growth, colony formation, migration and invasion capabilities (Fig. 4a-d and Supplementary Fig. 5D). However, ectopic overexpression of KPC1-EDT abolished, at least partly, the inhibitory effects by KPC1-WT on these malignant phenotypes, indicating a loss-of-function effect of p.M8V (Fig. 4a-d and Supplementary Fig. 5E).

The effects of KPC1 and its p.M8V editing on iCCA tumorigenesis were further tested in nude mice. Consistent with *in vitro* results, overexpression of KPC1-WT in RBE cells significantly reduced the subcutaneous tumor growth, whereas KPC1-EDT partly abolished the inhibitory effects of KPC1-WT on tumor growth (Fig. 4e-g, and Supplementary Fig. 5F and G). We further used the RBE cells that express a luciferase reporter to investigate the effects of KPC1-WT and KPC1-EDT on *in vivo* metastasis. Overexpression of KPC1-WT significantly reduced the lung metastasis burden (Fig. 4h), which consequently resulted in prolonged survival of the tumor-bearing mice (Fig. 4i). *Ex vivo* fluorescence imaging and histological analyses of the lung metastases also confirmed the inhibitory effect of KPC1-WT overexpression on HCC metastasis (Fig. 4j and k). On the contrary, KPC1-EDT partly reduced the inhibitory effects of KPC1-WT on tumor metastases (Fig. 4h-k). Taken together, these *in vitro* and *in vivo* observations suggested that KPC1 functions as a tumor suppressor in the development of iCCA and its p.M8V editing confers a loss-of-function effect.

### KPC1 p.M8V enhances activation of NF-κB signaling in iCCA cells

**Fig. 5:**
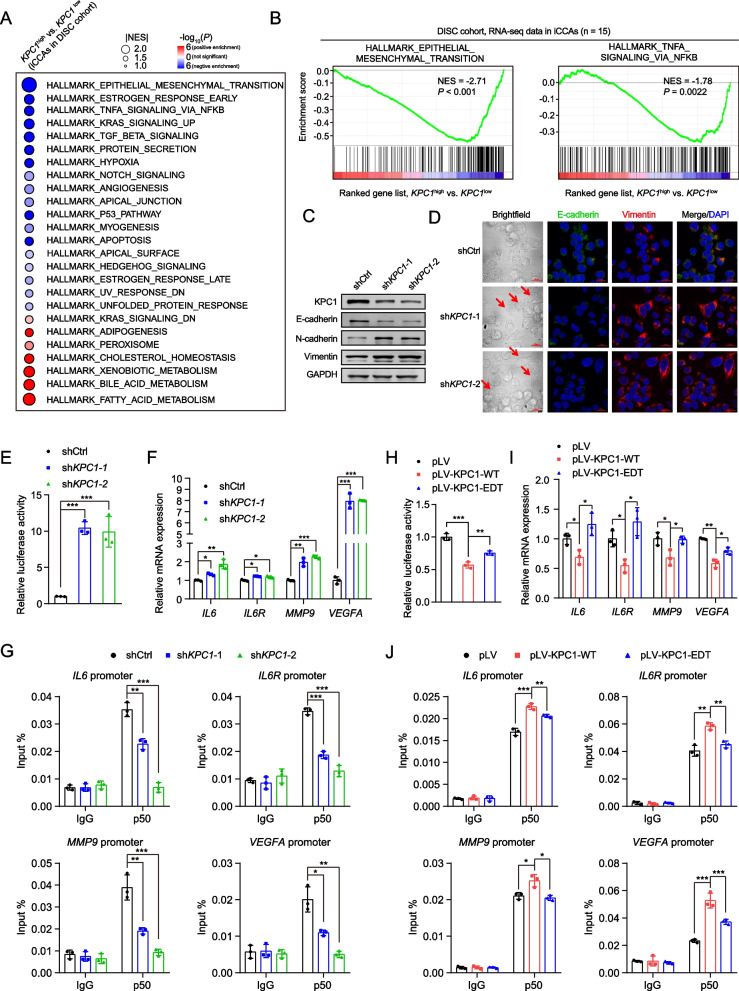
KPC1 p.M8V editing weakens the p50-mediated inactivation of NF-κB signaling.** a** KPC1-associated biological processes discriminated by gene set enrichment analyses (GSEA). GSEA was performed on the basis of the mean of the *KPC1* mRNA expression levels in iCCA tissues from the patients of DISC cohort (*KPC1*^High^ versus *KPC1*^Low^). NES, normalized enrichment score. **b** GSEA plots of the signatures of the epithelial-mesenchymal transition (Left panel) and TNF-α signaling via NF-κB (Right panel). **c** and **d** Immunoblotting (**C**) and immunofluorescence **(D)** assays in QBC939 cells showed that knockdown of *KPC1* induces epithelial-mesenchymal transition (EMT) evidenced by epithelial marker E-cadherin and mesenchymal marker N-cadherin or Vimentin. The arrows in (**D**) mark spindle-shaped cells. Scale bar in (**D**), 20 µm. **e** Luciferase reporter assays showing that knockdown of *KPC1* enhances the NF-κB signaling in QBC939 cells. **f** Knockdown of *KPC1* induces the mRNA expression levels of multiple downstream targets of p50, including *IL6*, *IL6R*, *MMP9* and *VEGFA*. **g** Chromatin immunoprecipitation (ChIP)-qPCR assays in QBC939 cells show that *KPC1* knockdown weakens the binding of p50 to the promoters of its targets *IL6*, *IL6R*, *MMP9* and *VEGFA*. **h** Luciferase reporter assays in QBC939 cells show that KPC1 p.M8V editing (KPC1-EDT) abolishes the inhibitory effect on NF-κB signaling by wide-type KPC1 (KPC1-WT). **i** Real-time PCR assays in QBC939 cells show that KPC1-EDT abolishes the inhibitory effect on expression of multiple downstream targets of p50 by KPC1-WT. **j** ChIP-qPCR assays in QBC939 cells show that KPC1-EDT abolishes the promoting effect on binding of p50 to the promoters of its targets by KPC1-WT. Data are presented as the mean ± standard deviation (s.d.). ^*^P < 0.05, ^**^P < 0.01 and ^***^P < 0.001; assessed by Student’s *t* test

Next, we sought to determine the underlying mechanisms by which KPC1 and its editing site p.M8V play their roles in iCCAs. Gene set enrichment analysis (GSEA) based on the RNA-seq data from the DISC cohort showed that “Epithelial-mesenchymal transition (EMT)” is inactivated iCCAs with higher *KPC1* expression levels (FDR *q* < 0.001; Fig. 5a-b and Supplementary Table 7). We further performed immunoblotting and immunofluorescence assays in QBC939 cells. The results showed that knockdown of *KPC1* induced EMT evidenced by the transition of cells from round to spindle, the decreased expression of E-cadherin, and the increased expression of N-cadherin and Vimentin (Fig. 5c and d). Furthermore, overexpression of wide-type KPC1 inhibits EMT, while overexpression of edited KPC1 (KPC1-EDT) abolishes this effect (Supplementary Fig. 6A and B). Additionally, we noticed that among the enriched top-ranked gene sets, the “TNF-α signaling *via* NF-κB” pathway was also negatively associated with the expression level of *KPC1* (FDR *q* = 0.0022; Fig. 5a and b, and Supplementary Table 7). Indeed, several previous studies have reported that KPC1 could induce the NF-κB transcriptional repressor p50:p50 homodimers in several types of human cancer cell [20, 36]. We therefore hypothesized that KPC1 might exert its tumor suppressive role in cholangiocarcinoma cells by affecting the NF-κB signaling activity.

To this end, we first performed luciferase reporter gene assays that are driven by NF-κB binding sites in cholangiocarcinoma cells. We observed that knockdown of *KPC1* markedly enhances the NF-κB luciferase reporter activity in QBC939 cells (Fig. 5e). Further, knockdown of *KPC1* in QBC939 cells significantly increased the mRNA levels of several established target genes of NF-κB p50, including *IL6*, *IL6R*, *MMP9* and *VEGFA* (Fig. 5f), which are central coordinators of tumorigenesis [36, 37]. Accordingly, chromatin immunoprecipitation (ChIP) assays further revealed that the *in situ* bindings of p50 to the promoters of *IL6*, *IL6R*, *MMP9* and *VEGFA* are significantly reduced upon knockdown of *KPC1* in QBC939 cells (Fig. 5g). The similar observations were obtained in RBE cells (Supplementary Fig. 6C-E).

We then assessed the effects of KPC1 p.M8V on the NF-κB response. As expected, overexpression of KPC1-WT in QBC939 cells significantly reduced the NF-κB luciferase reporter activity and the mRNA levels of *IL6*, *IL6R*, *MMP9* and *VEGFA*, and enhanced bindings of p50 to the promoters of these target genes; whereas KPC1-EDT overexpression abolished these effects (Fig. 5h-j). The similar observations were observed in RBE cells (Supplementary Fig. 6F-H). Taken together, these findings suggested that KPC1 functions as a suppressor of NF-κB pathway in cholangiocarcinoma cells; whereas the KPC1 p.M8V editing confers a loss-of-function effect on this pathway.

### KPC1 p.M8V editing weakens the ubiquitination of p105 and its cleavage to p50

**Fig. 6:**
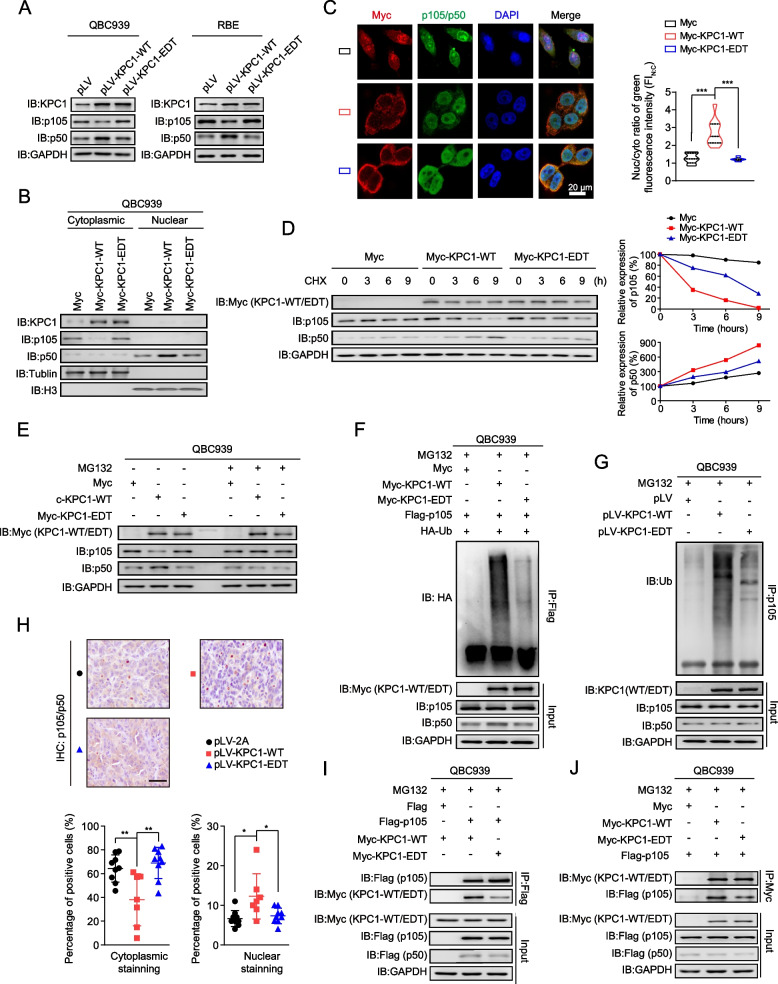
KPC1 p.M8V editing attenuates the ubiquitination of p105 and its cleavage to p50.** a** The effects of wide-type KPC1 (KPC1-WT) and KPC1 p.M8V editing (KPC1-EDT) on the levels of p105 and p50 proteins in QBC939 and RBE cells. **b** The effects of KPC1-WT or KPC1-EDT on the levels of p105 and p50 proteins in cytoplasmic and nuclear fractions of QBC939 cells. Tubulin and histone H3 were used as the cytoplasmic and nuclear markers, respectively. **c** The effects of KPC1-WT or KPC1-EDT on processing of p105 to p50 in QBC939 cells by immunofluorescence assays. Scale bars, 20 μm. ^***^P < 0.001; assessed by Wilcox rank sum test. FI_N:C_, the ratio of nuclear to cytoplasmic fluorescence intensity. **d** The effects of KPC1-WT or KPC1-EDT on the protein levels of p105 and p50 in QBC939 cells upon cycloheximide (CHX) treatment (50 μg/mL) for the indicated times. **e** The effects of KPC1-WT or KPC1-EDT on the p105 and p50 levels in QBC939 cells without or with MG132 treatment (20 μM). **f** The effects of KPC1-WT or KPC1-EDT on the ubiquitination of exogenous Flag-p105 in QBC939 cells. **g** The effects of KPC1-WT or KPC1-EDT on the ubiquitination of endogenous p105 in QBC939 cells. **h** Immunohistochemistry (IHC) assays for the p105/p50 proteins in xenografted tumor tissues from the nude mice of control, KPC1-WT and KPC1-EDT groups. Scale bars, 200 μm. ^*^P < 0.05 and.^**^P < 0.01; assessed by Wilcox rank sum test. **i** Co-immunoprecipitation (co-IP) of p105 in QBC939 cells transfected with the indicated constructs using a Flag-specific antibody. Lysates were immunoprecipitated with antibody against Flag followed by immunoblotting with antibodies to Myc (p105-bounded KPC1-WT or KPC1-EDT) and Flag (p105). **j** Co-IP of KPC1-WT/EDT in QBC939 cells transfected with the indicated constructs using a Myc-specific antibody. Lysates were immunoprecipitated with antibody against Myc followed by immunoblotting with antibodies to Flag (KPC1-WT- or KPC1-EDT-bounded p105) and Myc (KPC1-WT/EDT)

KPC1 has been shown to function as an E3 ubiquitin ligase for NF-κB1 p105, which facilitates the ubiquitinating and proteasomal processing of NF-κB1 p105 to p50 in multiple human cancer cells, and then leads to generation of excessive transcriptional repressor p50:p50 homodimers in place of the transcriptional activator p50:p65 heterodimers [36]. We therefore went on to test these processes in cholangiocarcinoma cells. Indeed, knockdown of *KPC1* resulted in an elevation of p105 protein, but a reduction of p50 protein levels in QBC939 cells (Supplementary Fig. 7A). Conversely, overexpression of KPC1-WT decreased the p105, but increased the p50 protein levels in QBC939 and RBE cells; whereas overexpression of KPC1-EDT did not reveal these effects (Fig. 6a). We further test these findings by biochemical fractionation assays. The p105 protein was predominantly located in cytoplasm, and p50 was predominantly located in nucleus of QBC939 cells, respectively (Supplementary Fig. 7B). Knockdown of *KPC1* increased p105 levels in the cytoplasmic fraction but decreased p50 levels in the nuclear fraction, respectively (Supplementary Fig. 7B). On the contrary, overexpression of KPC1-WT in QBC939 cells decreased the cytoplasmic p105 and increased nuclear p50, respectively; however, KPC-EDT did not show these effects (Fig. 6b). Due to the limitation of the antibody that cannot discriminate the endogenous p105 from p50 in the *in situ* immunofluorescence confocal microscopy assays, we applied the ratio of nuclear to cytoplasmic fluorescence intensity (FI_N:C_) as readout of the processing degree of p105 to p50. Consistently, we observed that FI_N:C_ is markedly reduced in QBC939 cells expressing KPC-EDT compared to that expressing KPC1-WT (Fig. 6c). Furthermore, through cycloheximide (CHX) studies, we found that *KPC1* knockdown prolongs the half-life of p105 protein and weakens the accumulation of p50 in QBC939 cells (Supplementary Fig. 7C). Conversely, overexpression of KPC1-WT led to a shortened half-life of p105 protein and increased accumulation of p50 in QBC939 cells (Fig. 6d). Again, the KPC1 p.M8V editing diminished these effects by KPC1-WT (Fig. 6d). Taken together, these findings suggested that KPC1 reduces the p105 stability and induces p50 protein levels in cholangiocarcinoma cells; whereas its p.M8V editing confers loss-of-function effects on these abilities.

We next investigated how KPC1 and its p.M8V regulate p105 and p50 protein levels in cholangiocarcinoma cells. We observed that the effects of KPC1 and its p.M8V on the p105 and p50 protein levels are inhibited by the proteasomal inhibitor MG132 in QBC939 cells (Fig. 6e and Supplementary Fig. 7D). The similar results were observed in RBE cells (Supplementary Fig. 7E), indicating the involvement of ubiquitin proteasome system. Thus, we overexpressed Flag-p105 along with HA-Ub in QBC939 cells to examine whether KPC1 regulates p105 ubiquitination. Indeed, we found that knockdown of *KPC1* weakens the ladder of ubiquitinated p105 (Ub-p105) products (Supplementary Fig. 7F); whereas overexpression of KPC1-WT leads to a more intense ladder of Ub-p105 (Fig. 6f). As expected, overexpression of KPC1-EDT didn’t show these effects (Fig. 6f). Consistently, endogenous ubiquitination assays showed that overexpression of KPC1-WT, but not KPC1-EDT, enhances the endogenous Ub-p105 (Fig. 6g). Similar results were obtained in RBE cells (Supplementary Fig. 7G and H).

We also examined the effects of KPC1 p.M8V on processing of p105 to p50 in xenograft tumor tissues from the nude mice. IHC assays showed that in KPC1-WT tumor tissues, the cells with positive cytoplasmic staining of p105 or p50 (designated as p105/p50, due to the limitation of the antibody that cannot discriminate the endogenous p105 from p50) are decreased, but the cells with positive nuclear staining of p105/p50 are increased compared to the empty control tumors (Fig. 6h). However, the KPC1-EDT tumors showed similar staining patterns of p105/p50 to the empty control tumors (Fig. 6h). Accordingly, the qRT-PCR assays showed that the promoting effects on the expression levels of p50 targets by KPC1-WT are observed in the xenograft tumor tissues; whereas the KPC1-EDT abolishes these effects (Supplementary Fig. 7I). We also performed IHC analyses in human samples from the VALI2 cohort, and observed higher cytoplasmic staining intensity of p105/p50 in iCCA tissues than in paired non-tumor liver tissues, but negative nuclear staining signal of p105/p50 in most of the samples (Supplementary Fig. 7J). Collectively, these findings suggested that KPC1-WT promotes the processing of NF-κB1 p105 to p50 through ubiquitinating p105, whereas its p.M8V editing confers loss-of-function effects.

### KPC1 p.M8V editing attenuates its binding to p105

Next, we investigated how KPC1 p.M8V editing reduces p105 ubiquitination and its processing to p50. First, the immunoblotting assays showed that both KPC1-WT and KPC1-EDT are predominantly expressed in the cytoplasm of QBC939 cells, and no significant difference in localization distributions between them was observed (Fig. 6b), which was further confirmed by immunofluorescence assays (Fig. 6c), indicating that the p.M8V editing does not affect the subcellular localization of KPC1. We then assessed whether the KPC1 p.M8V editing affects the interaction of KPC1 with p105. Indeed, when using the Flag-p105 as baits, the capabilities of KPC1-EDT binding to p105 were much weaker than that of KPC1-WT in both QBC939 and RBE cells (Fig. 6i and Supplementary Fig. 7K). Consistently, when using the Myc-KPC1-WT or Myc-KPC1-EDT as baits, the binding of p105 to KPC1-EDT was much weaker than that to KPC1-WT (Fig. 6j and Supplementary Fig. 7L). Taken together, these observations indicated that the KPC1 p.M8V editing weakens the binding of KPC1-EDT to p105, which in turn attenuates the KPC1-mediated p105 ubiquitination and its cleavage to p50.

### KPC1 p.M8V editing is required for ADAR1’s oncogenic function in iCCA

**Fig. 7:**
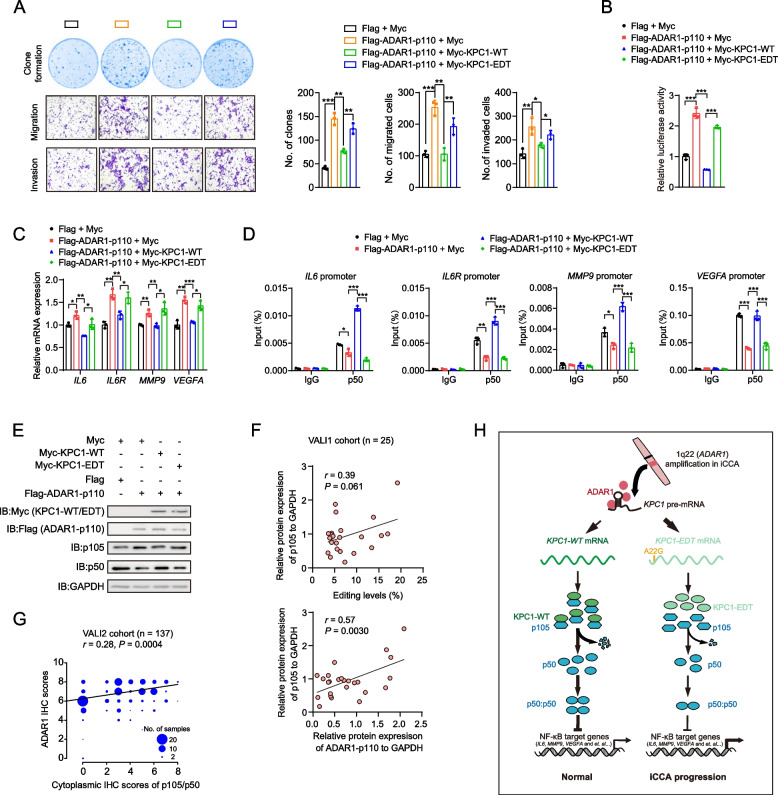
ADAR1 promotes iCCA progression, at least partly, through the KPC1 p.M8V editing.** a** The effects of overexpression of wide-type KPC1 (KPC1-WT) and KPC1 p.M8V editing (KPC1-EDT) on plate colony formation, migration and invasion capacities of the ADAR1-p110-overexpressed QBC939 cells. **b**-**d** The effects of overexpression of KPC1-WT or KPC1-EDT on NF-κB response in *ADAR1-p110*-overexpressed QBC939 cells by NF-κB reporter assays (**B**), qRT-PCR assays (**C**) and ChIP-qPCR assays (**D**). The data are presented as the mean ± standard deviation (s.d.). ^*^P < 0.05, ^**^P < 0.01 and ^***^P < 0.001; assessed by Student’s *t* test. **e** The effects of overexpression of KPC1-WT or KPC1-EDT on p105 and p50 proteins levels in QBC939 cells upon overexpression of ADAR1-p110. **f** The correlation of p105 protein levels with the *KPC1* RNA A22G editing levels (Up panel) or ADAR1-p110 protein levels (Down panel) in iCCA tissues from the patients of VALI1 cohort. The correlation efficient and *P* value were calculated by Spearman’s rank correlation analysis. **g** The correlation of ADAR1 protein levels with the cytoplasmic p105/p50 protein levels in iCCA tissues from the patients of VALI2 cohort. The correlation efficient and *P* value were calculated by Spearman’s rank correlation analysis. **h** Functional model of KPC1 p.M8V editing in the development of iCCA

We next assessed whether the oncogenic role of ADAR1 in the development of iCCA depends on KPC1 and its editing site p.M8V. We first confirmed that the presence of ADAR1 does not affect the total expressions of KPC1 at both mRNA and protein levels in QBC939 and RBE cells (Supplementary Fig. 8A). Next, we observed that overexpression of KPC1-WT reduces the promoting effects of ADAR1-p110 overexpression on QBC939 cells growth, migration and invasion; whereas KPC1-EDT overexpression abolished, at least partly, these effects by KPC1-WT (Fig. 7a). Similar results were observed in RBE cells (Supplementary Fig. 8B). These findings thus suggest that KPC1-WT neutralizes the oncogenic role of ADAR1 in iCCA, while its editing mutation p.M8V confers the loss-of-function effect.

We then investigated whether ADAR1-p110 activates NF-κB pathway in a KPC1 p.M8V editing-dependent manner. We first examined the role of ADAR1 in regulation of NF-κB pathway. Indeed, the NF-κB luciferase reporter, qRT-PCR and ChIP-qPCR assays consistently showed that overexpression of ADAR1-p110 significantly potentiates the NF-κB response in QBC939 cells (Fig. 7b-d). Next, we found that overexpression of KPC1-WT, but not KPC1-EDT, reduces these ADAR1-p110 overexpression-driven NF-κB responses in QBC939 cells (Fig. 7b-d), suggesting the involvement of KPC1 p.M8V editing in the ADAR1/NF-κB axis.

Further, we observed that KPC1-WT, but not KPC1-EDT, significantly abrogates the promoting effect of ADAR1-p110 on p105 protein levels, and the inhibitory effect of ADAR1-p110 on p50 protein levels in QBC939 cells (Fig. 7e), suggesting that the ADAR p110-mediated over-editing of KPC1 p.M8V reduces the processing of p105 to p50. Of note, we observed a marginal positive correlation between the *KPC1* A22G editing levels and p105 protein levels in iCCA tissues from the patients of VALI1 cohort (*r* = 0.39, *P* = 0.061; Fig. 7f and Supplementary Fig. 8C). Additionally, the protein levels of ADAR1-p110 were shown to be significantly correlated with the p105 protein levels in this cohort (*r* = 0.57, *P* = 0.0030; Fig. 7f and Supplementary Fig. 8C). A similar correlation between the protein levels of ADAR1-p110 and p105 was observed in iCCA tissues from the patients of VALI2 cohort (*r* = 0.28, *P* = 0.00040; Fig. 7g). Collectively, these findings indicated that KPC1 is, at least, one of the downstream substrates (*i.e.*, editing targets) by ADAR1-p110, and its editing mutation p.M8V is required for ADAR1-p110 to play its oncogenic role in the development of iCCA.

## Discussion

Accumulating evidences have established the links between A-to-I RNA editing and cancers initiation and progression. However, only a few non-synonymous RNA editing events have been functionally characterized. We here performed an integrative omics analysis and identified a collection of non-synonymous A-to-I RNA editing events in iCCAs. Among them, we characterized an ADAR1-mediated recoding editing of KPC1 (p.M8V), which was found to be relevant to iCCA pathogenesis, through neutralizing the tumor suppressive role of the wide-type KPC1. We further revealed that the p.M8V editing of KPC1 attenuates its E3 enzyme capability towards NF-κB1 p105, and then reduces the processing of p105 to p50, thereby facilitating the activation of oncogenic NF-κB signaling (Fig. 7h). To our best knowledge, our findings for the first time established a close link between the A-to-I RNA editing aberrations and iCCA progression.

KPC1 has been reported to be inactivated and inhibit anchorage-independent growth of breast cancer, bone osteosarcoma, glioma and melanoma cells [20, 36]. Here, we provided evidences in principle that KPC1 inhibits iCCA progression through enhancing p105 ubiquitination and its cleavage to p50, consistent with the findings in the other cancers in previous studies [20, 36]. Of note, KPC1 has been shown to target the cyclin-dependent kinase inhibitor p27 for degradation in fibroblasts [38]. However, KPC1 didn’t affect the p27 protein levels in glioma cells [36]. We observed that the p27 protein levels are not changed under enforced expression of either KPC1-WT or KPC1-EDT in QBC939 cells (Supplementary Fig. 9), indicating the p27-non-dependent mechanism for KPC1 in the development of iCCA. These inconsistencies among different types of cancer may be due to the cell type specificity.

Given the critical roles of KPC1 in tumorigenesis, the mechanisms underlying its inactivation have not been fully understood. A recent study has revealed that *KPC1* mRNA was directly targeted by miR-155-5p [20]. Here, we provided evidence that the *KPC1* transcripts are over-edited at A22G by ADAR1 in iCCAs. Further, the *in vitro* and *in vivo* studies provided direct evidences that the edited KPC1 confers loss-of-function phenotypes. Thus, the editing of *KPC1* transcripts might serve as a novel layer of “somatic mutations” and a novel modulator of iCCA pathogenicity. Together, our findings established a novel mechanism of weakening KPC1’s tumor suppressive roles in iCCA.

Different patterns of ADAR-mediated A-to-I mRNA editing in multiple types of cancer have been observed [29]. The divergence of RNA editing patterns was likely dominated by the dysregulation of ADARs, especially the ADAR1 [29]. In the meanwhile, either oncogenic or tumor suppressive roles of ADARs have been reported. In contrast to the generally oncogenic functions of ADAR1 involved in breast cancer [39], hepatocellular carcinoma [12, 34], lung cancer [40], gastric cancer [15], myeloma [10, 41] and leukemia [42], ADAR1 also exhibited tumor suppressive roles in metastatic melanoma [43, 44]. These inconsistent findings are likely attributed to the selectively deaminated substrates of ADAR1 [43] or other undetermined RNA editing-independent mechanism [44]. Indeed, in the present study, an ADAR1-mediated RNA over-editing pattern was observed in iCCAs, which is in accordance with high-frequency genomic amplification of *ADAR1* in iCCA tissues (Fig. 2a). Further, functional studies also established that ADAR1 exerts its oncogenic role *via* NF-κB signaling pathway, which has been shown to be essential for metastasis of iCCA [45]. Collectively, our results highlighted the ADAR1-KPC1-NF-κB axis in iCCA progression, therefore suggesting its potential therapeutic vulnerability for iCCAs.

## Conclusions

This study identified a collection of non-synonymous RNA editing events in iCCAs. Among them, we demonstrated that the p.M8V over-editing of KPC1, which is dominated by ADAR1, confers loss-of-function effects in the development of iCCA by enhancing NF-κB signaling through attenuation of p105 degradation and cleavage to p50. These findings provided new insights into the mechanisms of RNA editing in tumor progression through modulating ubiquitin E3 ligase activation and NF-κB signaling. The importance of the ADAR1-KPC1-NF-κB axis in the development of iCCA suggested new application prospects in treatment of this malignancy.

## Supplementary Information

**Additional file 1: Supplementary file 1.** 

**Additional file 2: Supplementary file 2.**

## Data Availability

The RNA-seq and SNP array data sets of 15 pairs of iCCA tissues and adjacent non-tumor liver tissues in this study have been deposited in NCBI’s GEO under accession number GSE119336 (for RNA-seq) and GSE119335 (for SNP array), respectively. The WES data set of 15 pairs of iCCA tissues and adjacent non-tumor liver tissues in this study have been deposited in the EGA (European Genome-phenome Archive) database (Accession No. EGAS00001002331) and the International Cancer Genome Consortium (ICGC) data portal (http://dcc.icgc.org/; release 25, June 8th, 2017). All the other data supporting the findings of this study are available from the corresponding author upon reasonable request.

## Abbreviations

A-to-I: Adenosine-to-Inosine
ADARs: Adenosine Deaminase Acting on RNA Enzymes
BLI: Representative Bioluminescence
CCK-8: Cell Counting Kit-8
Co-IP: Co-Immuoprecipitation
ChIP: Chromatin Immunoprecipitation
CHX: Cycloheximide
CAN: Copy Number Alteration
DMEM: Dulbecco’s Modified Eagle’s Medium
DFS: Disease-Free Survival
EMT: Epithelial-Mesenchymal Transition
EDT: Edited
FBS: Fetal Bovine Serum
FI_N:C_: The ratio of Nuclear to Cytoplasmic Fluorescence Intensity
GEO: Gene Expression Omnibus
GSEA: Gene Set Enrichment Analysis
H&E: Hematoxylin-Eosin
iCCA: Intrahepatic Cholangiocarcinoma
IHC: Immunohistochemistry
KPC1: Kip1 Ubiquitination-Promoting Complex 1
LIHC: Liver Hepatocellular Carcinoma
NTBD: Non-Tumor Bile Duct tissues
NTL: Non-Tumor Liver tissues
NES: Normalized Enrichment Score
OS: Overall Survival
PLC: Primary Liver Cancer
RPMI-1640: Roswell Park Memorial Institute-1640 medium
qRT-PCR: Quantitative Real-Time PCR
RING: Really Interesting New Gene domain
SNPs: Single Nucleotide Polymorphisms
shRNAs: Short-hairpin RNAs
T: ICCA Tissues
TCGA-CHOL: The Cancer Genome Atlas-Cholangiocarcinoma
VAF: Variation Frequency
WES: Whole-Exome Sequencing
WT: Wide-Type
