# Supplementary material for: The A-to-I editing of KPC1 promotes intrahepatic cholangiocarcinoma by attenuating proteasomal processing of NF-κB1 p105 to p50
Source: J Exp Clin Cancer Res. 2022 Dec 8;41:338. doi: 10.1186/s13046-022-02549-1 (PMC9730630; doi:10.1186/s13046-022-02549-1)
Supplement: Supplementary file 1 — Additional file 1: Supplementary file 1. [file 13046_2022_2549_MOESM1_ESM.doc]

**Supplementary Material**

**The A-to-I editing of KPC1 promotes intrahepatic cholangiocarcinoma by attenuating proteasomal processing of NF-κB1 p105 to p50**

Chengming Gao, Guangming Zhou, Jie Shi, Peipei Shi, Liang Jin, Yuanfeng Li, Xiaowen Wang, Song Liao, Han Yan, Yiming Lu, Yun Zhai, Jinxu Zhang, Haitao Zhang, Hongxing Zhang, Chenning Yang, Pengbo Cao, Shuqun Cheng and Gangqiao Zhou.

Correspondence to: Dr. Gangqiao Zhou, E-mail: zhougq114@126.com; Dr. Shuqun Cheng, E-mail: chengshuqun@aliyun.com; and Dr. Pengbo Cao, E-mail: birchcpb@163.com.

**Supplementary methods;**

**Supplementary figures 1-9;**

**Supplementary tables 1-8.**

**Supplementary methods**

**RNA-seq and data analyses**

RNA-seq was performed at MyGenostics Inc. (Beijing, China). The libraries for RNA-seq were prepared using the Illumina Tru-Seq RNA Sample Preparation v2 Kit according to the manufacturer’s instructions, and then subjected to massively parallel sequencing using Illumina HiSeq3000. The TopHat (v2.0.10) [^1^](#_ENREF_1) was used to align the reads to the NCBI human reference genome assembly (build 36.1) and the UCSC annotated genes with default parameters. Samtools rmdup was used to remove the identical reads (PCR duplicates) that mapped to the same location. The RNA-seq generated an average of 24.1 million reads per sample uniquely aligned to reference genome, with substantial coverage (an average of 74.1%) for the majority of the known mRNA transcripts (**Supplementary Fig. 1A** and **Supplementary Table 2**). The Cufflinks (v2.1.1) [^2^](#_ENREF_2) was used to estimate the gene expression levels based on the map generated by TopHat. The expression levels for each gene were normalized to fragments per kilobase of exon per million fragments mapped (FPKM) to facilitate the comparison of transcript levels among samples.

**Whole-exome sequencing and SNVs detection**

Genomic DNA was captured using the SureSelect Human All Exon V5+UTRs kit (Agilen, USA). Captured DNAs were then subjected to massively parallel sequencing using Illumina HiSeq2000. WES was performed by CapitalBio Corp. (Beijing, China). The sequencing reads were aligned to the NCBI human reference genome assembly (build 36.1) by BWA (v0.5.9) with default parameters. Samtools rmdup was used to remove the identical reads (PCR duplicates) that mapped to the same location. On average, a total of 11.7 giga base (Gb) of high-quality mappable sequence per sample were aligned to the targeted exome regions. The mean sequencing depth was 92.7 ×, and 98.8% of the targeted regions were covered at least 10 × (**Supplementary Table 3**). The somatic single nucleotide variants (SNVs) were detected by VarScan (v2.2.5) [^3^](#_ENREF_3). To eliminate the contamination from common germline mutations, the variants with minor allele frequency (MAF) > 0.001 in dbSNP database (version 135) and the 1000 Genomes Project (February 2012 data release) were removed. We used the RefSeq annotation database for transcript identification and for determining the amino acid changes.

**qRT-PCR assays**

Total RNAs from the tissues of iCCA patients, the cell lines and the subcutaneous tumors were extracted by TRIzol (Sigma, USA). To quantify the gene expression levels in cells or clinical samples, 500 ng of total RNAs were used to synthesize the cDNAs with the PrimeScript RT Master Mix (RR037A, TaKaRa, Japan), following the standard protocols provided by the manufacturer. We performed the qRT-PCR assays using the SYBR Green PCR Kit (TaKaRa, Japan) and the primers (**Supplementary Table 8**) for the following genes: *ADAR1*, *ADAR2*, *IL6*, *IL6R*, *MMP9*, *VEGFA*, *β-actin* and *GAPDH*. The amplification protocol consists of incubations at 95 °C for 15 seconds (s), 60 °C for 1 minute (min), and 72 °C for 1 min for 40 cycles. Quantification was performed using the iQ5™ Optical Module (BIO-RAD, USA). The relative mRNA expression levels of these genes were given as 2^−ΔΔCt^ and normalized to the relative expression levels that were detected in the adjacent non-tumor liver tissues or corresponding control cells.

**Vector construction and transfection**

We amplified the cDNAs encoding the full-length coding DNA sequences (CDS) of *KPC1-WT* (NCBI No. NM_022064) and *KPC1-EDT* (p.M8V), *ADAR1-p110* (NM_015841) and -*p150* (NM_015840) isoforms, *ADAR2* (NM_001112) and *p105* (NM_003998) by KOD-Plus-Neo high-fidelity thermostable DNA polymerase (Toyobo, Japan). Then, the purified PCR products of *KPC1* were subcloned into both the pLV-EGFP(2A)-Puro lentiviral vector and pCMV-Myc vector. The purified PCR products of *ADAR1-p110*, *ADAR1-p150*, *ADAR2* and *p105* were subcloned into the pLV-neo lentiviral vector and pFlag-CMV-2 vector, respectively. For rescue assays, we generated the *ADAR1-p110* mutant construct that preserves the native amino acid sequence but contains six point mutations within the sh*ADAR1*-1 targeting sequence. PCR-directed mutagenesis was performed using an inner forward or reverse primer containing those six nucleotide alterations (forward, 5’-GAGAACGGAGAAGGCACAATCCCAGTAGAGTCAAGCGATATT-3’; reverse, 5’-AATATCGCTTGAGTCTACTGGGATTGTGCCTTCTCCGTTCTC-3’) with the corresponding external primer. The constructs were transfected into target cells using Lipofectamine 2000 (11668-019, Invitrogen, USA) according to the manufacturer’s instructions. For all transfections, empty vector was used as the control vector. The *ADAR1-p110* and *-p150* cDNAs were provided by Prof. Leilei Chen (Cancer Science Institute of Singapore, Singapore); The *ADAR2* and *p105* cDNAs were provided by Prof. Jiahuai Han (Xiamen University, Xiamen City, China); The *KPC1* cDNA was provided by Prof. Aaron Ciechanover (Technion Integrated Cancer Center, Israel).

**Immunofluorescence assays**

For detecting the subcellular location of KPC1-WT or KPC1-EDT and NF-κB1 p105/p50 in iCCA cells, QBC939 cells were transfected with 2.0 μg of the Myc-tagged wild-type or edited KCP1 plasmid. After 36 h, these cells (5 × 10^5^) were grown on cover slips for one day, washed three times with 1 × PBS, and fixed with 4% paraformaldehyde for 20 min at room temperature, followed by incubation in 0.1% Triton X-100-PBS for 15 min. The cover slips were washed three times with 1 × PBS, incubated with 3% BSA-PBS for 30 min (to block the non-specific binding), and were incubated (for 24 h at 4 °C) with the NF-κB1 p105/p50 rabbit antibody (1:200; 13586, Cell Signaling Technology, USA) or Myc Tag Mouse antibody (1:50; 60003-2-Ig, Proteintech, USA) in blocking solution. Following extensive washing with 1 × PBS, the covers lips were incubated with FITC-conjugated anti-rabbit (1:50; CW0114, CWBIO, China) and Cy3-conjugated anti-mouse (1:50; CW0145, CWBIO, China) antibodies for 60 min, followed by three times washes with 1 × PBS. The cells were then stained with 4’-6’-diamidino-2-phenylindole (DAPI) in mounting medium. Staining was examined using confocal microscope (ELYRA S1, ZEISS, Germany). There are no available antibodies specific for p105 and p50, thus the ratio of nuclear to cytoplasmic fluorescence intensity (FI_N:C_) was used as a readout of processing of p105 to p50.

**Immunohistochemistry (IHC) assays**

Slides of each biopsy were stained with hematoxylin-eosin for routine histological evaluation. The slides were washed in xylene to remove the paraffin and then rehydrated through serial dilutions of alcohol, followed by washings with a solution of phosphate-buffered saline (PBS, pH 7.2). All the subsequent washes were buffered via the same protocol. The slides were then incubated with 3% H_2_O_2_ for 10 min to reduce the non-specific staining. Treated slides were placed in a citrate buffer (pH 6.0) and heated in a pressure cooker for 2 min. The slides were then incubated for overnight at 4 °C with primary antibodies separately as indicated. After washing, the slides were treated by the MaxVisionTM HRP-Polymer anti-Rabbit IHC Kit (Maxim Co., China). Then, all the slides were stained with 3, 3-diaminobenzidine tetra-hydrochloride (DAB) and the cells were counter-stained with hematoxylin. The slides were mounted with gum for examination and captured by the Olympus BX51 microscopic/Digital Camera System for study comparison. The antibodies used in this study include anti-ADAR1 (1:50; sc-73408, Santa Cruz, USA), anti-p105/p50 (1:200; NB100-56583SS, Novus Biologicals, USA) and anti-Ki-67 (1:250; ab16667, Abcam, USA). The IHC signals in mice xenografts were scored by the proportion of cells with positive staining by indicated antibody. The IHC signals in tissues were scored as previously described [^4^](#_ENREF_4). Briefly, a proportion score was assigned representing the estimated proportion of positive staining tumor cells (0, none; 1, < 1/100; 2, 1/100 to < 1/10; 3, 1/10 to < 1/3; 4, 1/3 - 2/3; and 5, > 2/3). Average estimated intensity of staining in positive cells was assigned an intensity score (0, none; 1, weak; 2, intermediate; and 3, strong). These two parameters were then combined and an overall score was obtained (0 or 2 - 8).

**Genomic copy number variation analyses**

The genomic copy number variation (CNV) status at *ADAR1* and *KPC1* loci in iCCAs from the DISC cohort were determined using the genome-wide single nucleotide polymorphisms (SNPs) genotyping data sets (GEO Accession No.: GSE119335), which were profiled by Affymetrix Genome-Wide Human SNP Array 6.0. Copy-number was estimated using Robust Multichip Analysis (CRMA, v2) method, which was used to preprocess the probe signal intensities from each raw .CEL file. Briefly, this procedure consists of a calibration for offset and global crosstalk between alleles, and normalized for probe sequence effects and fragment-length effects. Then, raw copy number was calculated by paired analysis, which takes the matched non-tumor liver tissue as reference for each tumor. Segmented copy number profiles were analyzed using the circular binary segmentation (CBS) algorithm with default parameters in the R package “DNA-copy” [^5^](#_ENREF_5). For each sample, the segmentation profile was median-centered. Germline CNVs reported in the Database of Genomic Variants (DGV, 2015-07-23) were then filtered out from the segmentation profiles. Segments that overlapped with normal CNVs (> 50%) were also removed. The copy number gains were defined as those with Log ratio > 0.3, whereas the copy number losses were defined as Log ratio < -0.3. We also performed genomic CNV analyses in iCCAs from an independent dataset (GSE49666 from the Gene Expression Omnibus [GEO] database; n = 10) from a previous study [^6^](#_ENREF_6) and TCGA-CHOL dataset (n = 29). The raw .CEL data of GSE49666 were downloaded and subjected to the CNV analyses as described above. For the TCGA-CHOL dataset, the level-3 CNV segment data were obtained and directly subjected to subsequent analyses. The CNV segment data were visualized in the integrative genomics viewer (IGV).

**Neighbor preferences analyses**

The neighbor preferences of the identified A-to-I RNA editing sites were estimated and visualized by WebLogo [^7^](#_ENREF_7). The neighboring (± 10) nucleotides of each A-to-I RNA editing sites identified in this study were assembled and subjected to WebLogo.

**Prediction of RNA secondary structure**

The secondary structure of KPC1 pre-mRNA sequences containing the edited site (100 nucleotides upstream and 100 nucleotides downstream from the edited site) were predicted by the RNAfold in ViennaRNA Package [^8^](#_ENREF_8).

**Gene set enrichment analyses (GSEA)**

The enrichment of underlying molecular mechanism was assessed by GSEA [^9^](#_ENREF_9) based on the RNA-seq data (GEO Accession No.: GSE119336) of iCCA tissues from the discovery cohort (n = 15). The GSEA was performed based on the median of *KPC1* expression levels (*KPC1*^high^ *vs*. *KPC1*^low^) to determine the biological pathways documented in the Molecular Signatures Database (MSigDB, v 6.1) that are altered after *KPC1* dysregulation. Statistical significance was calculated by permuting the gene set 1,000 times with a significance threshold of false discovery rate (FDR) *q* value less than 0.05.

**Supplementary references**

1. Trapnell C, Pachter L, Salzberg SL. TopHat: discovering splice junctions with RNA-Seq. *Bioinformatic.s* 2009, **25**(9)**:** 1105-11.

2. Trapnell C, Williams BA, Pertea G, Mortazavi A, Kwan G, van Baren MJ*, et al.* Transcript assembly and quantification by RNA-Seq reveals unannotated transcripts and isoform switching during cell differentiation. *Nat Biotechnol.* 2010, **28**(5)**:** 511-5.

3. Koboldt DC, Zhang Q, Larson DE, Shen D, McLellan MD, Lin L*, et al.* VarScan 2: somatic mutation and copy number alteration discovery in cancer by exome sequencing. *Genome Res.* 2012, **22**(3)**:** 568-76.

4. Zhang H, Zhai Y, Hu Z, Wu C, Qian J, Jia W*, et al.* Genome-wide association study identifies 1p36.22 as a new susceptibility locus for hepatocellular carcinoma in chronic hepatitis B virus carriers. *Nat Genet.* 2010, **42**(9)**:** 755-8.

5. Olshen AB, Venkatraman ES, Lucito R, Wigler M. Circular binary segmentation for the analysis of array-based DNA copy number data. *Biostatistics* 2004, **5**(4)**:** 557-572.

6. Chan-On W, Nairismagi ML, Ong CK, Lim WK, Dima S, Pairojkul C*, et al.* Exome sequencing identifies distinct mutational patterns in liver fluke-related and non-infection-related bile duct cancers. *Nat Genet.* 2013, **45**(12)**:** 1474-8.

7. Crooks GE, Hon G, Chandonia JM, Brenner SE. WebLogo: a sequence logo generator. *Genome Res.* 2004, **14**(6)**:** 1188-90.

8. Lorenz R, Bernhart SH, Honer Zu Siederdissen C, Tafer H, Flamm C, Stadler PF*, et al.* ViennaRNA Package 2.0. *Algorithms Mol Biol.* 2011, **6:** 26.

9. Subramanian A, Tamayo P, Mootha VK, Mukherjee S, Ebert BL, Gillette MA*, et al.* Gene set enrichment analysis: a knowledge-based approach for interpreting genome-wide expression profiles. *Proc Natl Acad Sci U S A.* 2005, **102**(43)**:** 15545-50.


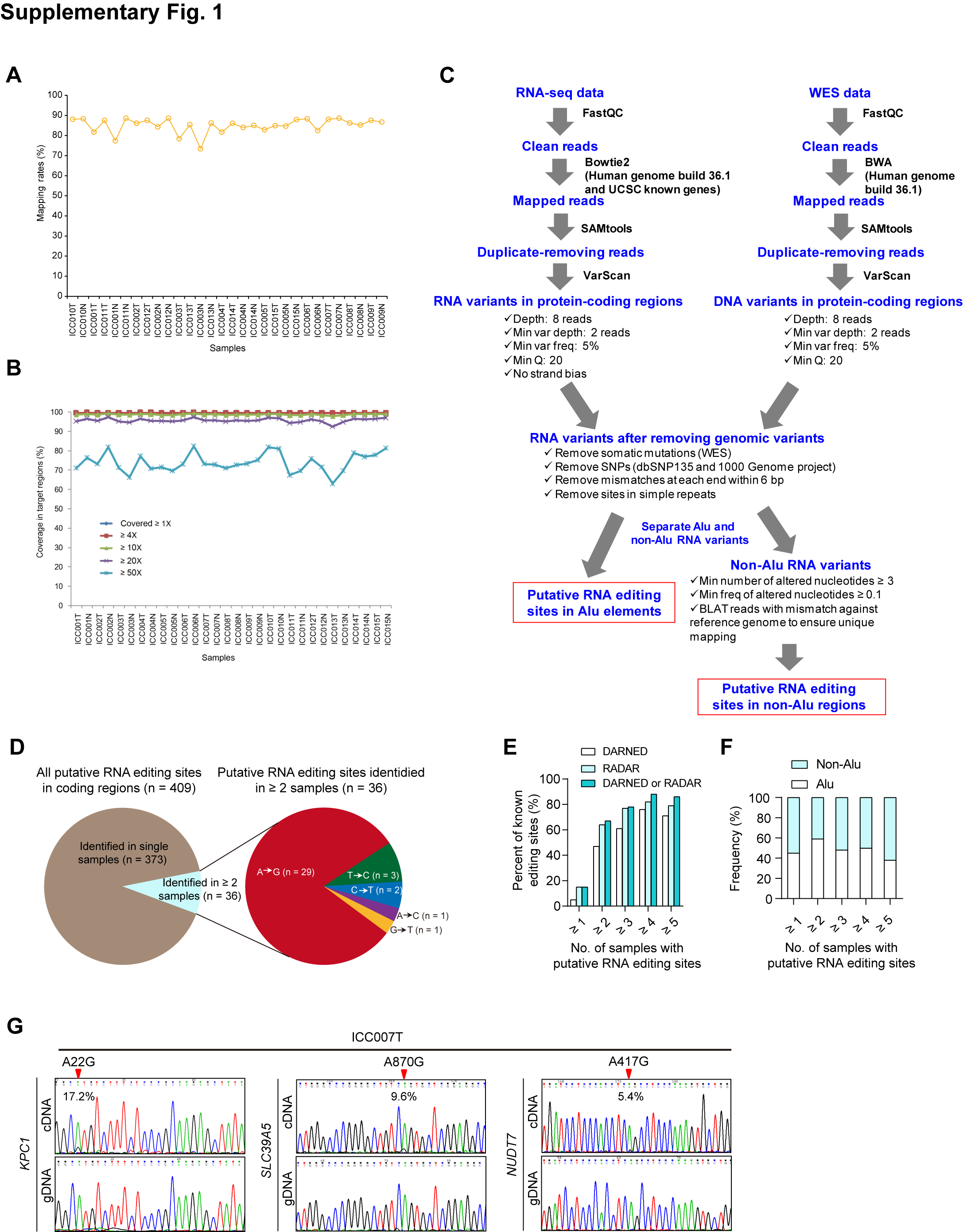


**Supplementary Fig. 1 Identification of A-to-I (G) RNA editing sites at the protein-coding regions in iCCAs genomes.**

**A** Mapping rates of the RNA sequencing (RNA-seq) data for each tumor tissue and its paired non-tumor liver tissue from the DISC cohort (n = 15). The sequencing reads were aligned to the human genome (hg19 build) and the UCSC annotated genes by Bowtie2.

**B** Coverage of the whole-exome sequencing (WES) data for each tumor tissue and its paired non-tumor liver tissue from the DISC cohort (n = 15). The sequencing reads were aligned to the human genome (hg19 build) by BWA (v0.5.9).

**C** The flowchart for identifying the RNA editing sites by use of the RNA-seq data.

**D** Distribution of all putative protein-coding RNA editing sites according to the frequency of occurrence and types of substitution.

**E** Proportion of the putative editing sites annotated in known RNA editing databases DARNED and RADAR. DARNED, a DAtabase of RNa EDiting in humans (<https://darned.ucc.ie/>); RADAR, a Rigorously Annotated Database of A-to-I RNA editing ([http://RNAedit.com](http://RNAedit.com/)).

**F** Proportion of the putative editing sites within or outside Alu elements.

**G** Exemplary presentation of three newly identified A-to-I RNA editing sites that were confirmed by Sanger sequencing in genomic DNAs (gDNA) and complementary DNAs (cDNA) from the tumor tissue of an iCCA patient (ICC007T) in the DISC cohort. Red triangles highlight the editing sites.


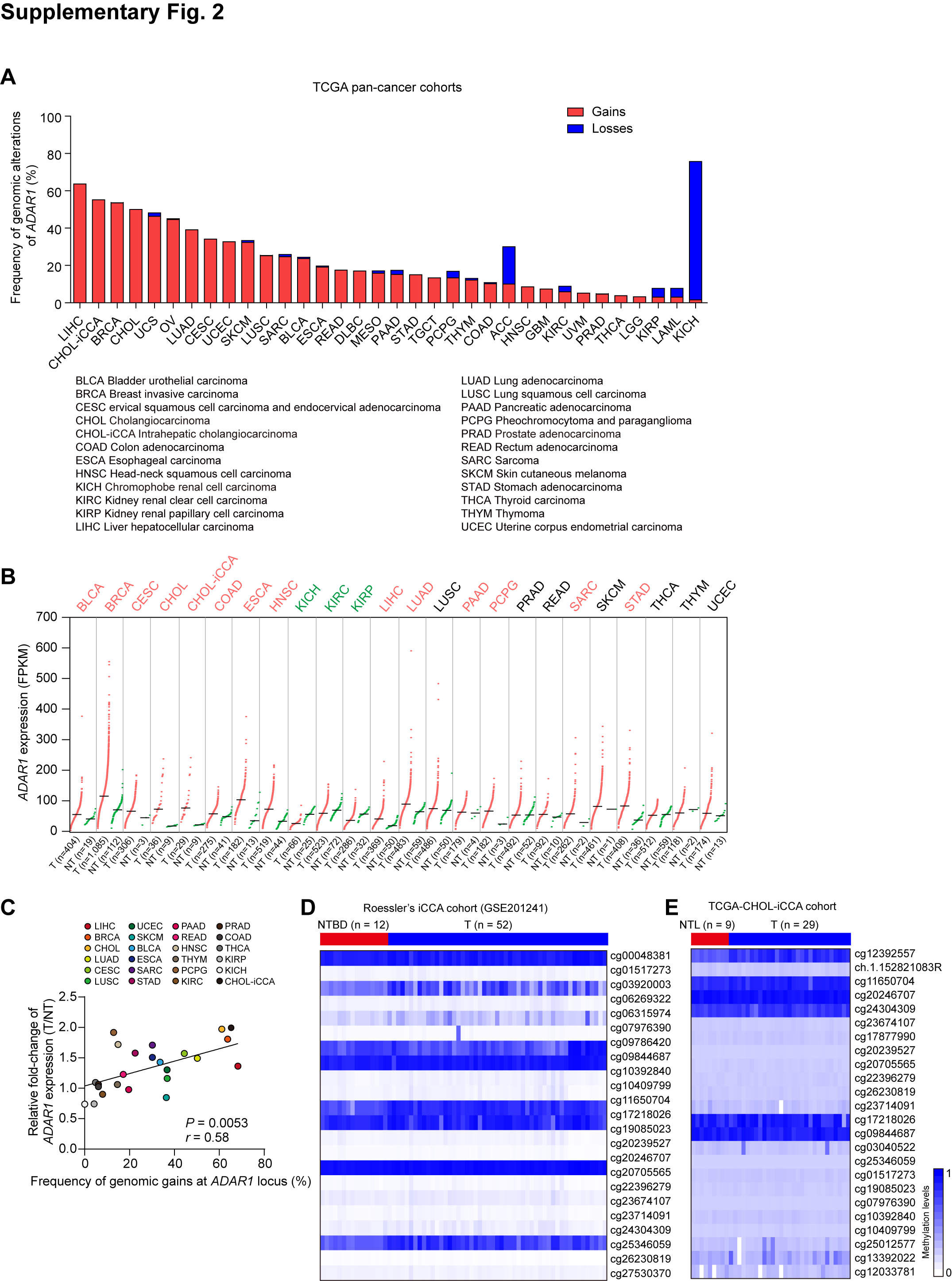


**Supplementary Fig. 2** **Alterations of *ADAR1* across cancer types in TCGA project.**

**A** Frequencies of genomic copy number alterations at *ADAR1* locus in multiple types of cancer. The whole-genome SNP genotyping profiles for cancers were obtained from The Cancer Genome Atlas (TCGA). Copy number gain was defined as Log ratio (LR) segment mean > 0.3 and loss as LR segment mean < -0.3, respectively.

**B** The *ADAR1* mRNA expression levels in tumor tissues (T) and non-tumor tissues (NT) from several types of cancer in TCGA dataset. The expression data were obtained from GEPIA ([http://gepia.cancer-pku.cn](http://gepia.cancer-pku.cn/)). Expression differences were calculated by Wilcoxon rank-sum test and considered to be statistically significant at *P* < 0.05; the green font indicates the *ADAR1* mRNA expression levels are significantly decreased in tumor tissues compared to the non-tumor tissues, while the red font indicates the *ADAR1* mRNA expression levels are significantly increased in tumors compared to the non-tumor tissues.

**C** The correlation between the frequencies of *ADAR1* genomic gains and the relative fold-changes of *ADAR1* expression (tumor/non-tumor) in multiple types of cancer from TCGA dataset.

**D** Heatmap of methylation profile of CpGs at the *ADAR1* promoter region (chr1:154,550,863 - 154,600,788 bp [hg19 build]). The DNA methylation dataset (GSE201241) were derived from the iCCA tumor tissues (T; n = 52) and non-tumor bile duct tissues (NTBD; n = 12) of Roessler’s cohort.

**E** Heatmap of methylation profile of CpGs at the *ADAR1* promoter region (chr1:154,550,863 - 154,600,788 bp [hg19 build]). The DNA methylation dataset were derived from the iCCA tumor tissues (T; n = 29) and non-tumor liver tissues (NTL; n = 9) of the TCGA-cholangiocarcinoma (TCGA-CHOL) cohort.


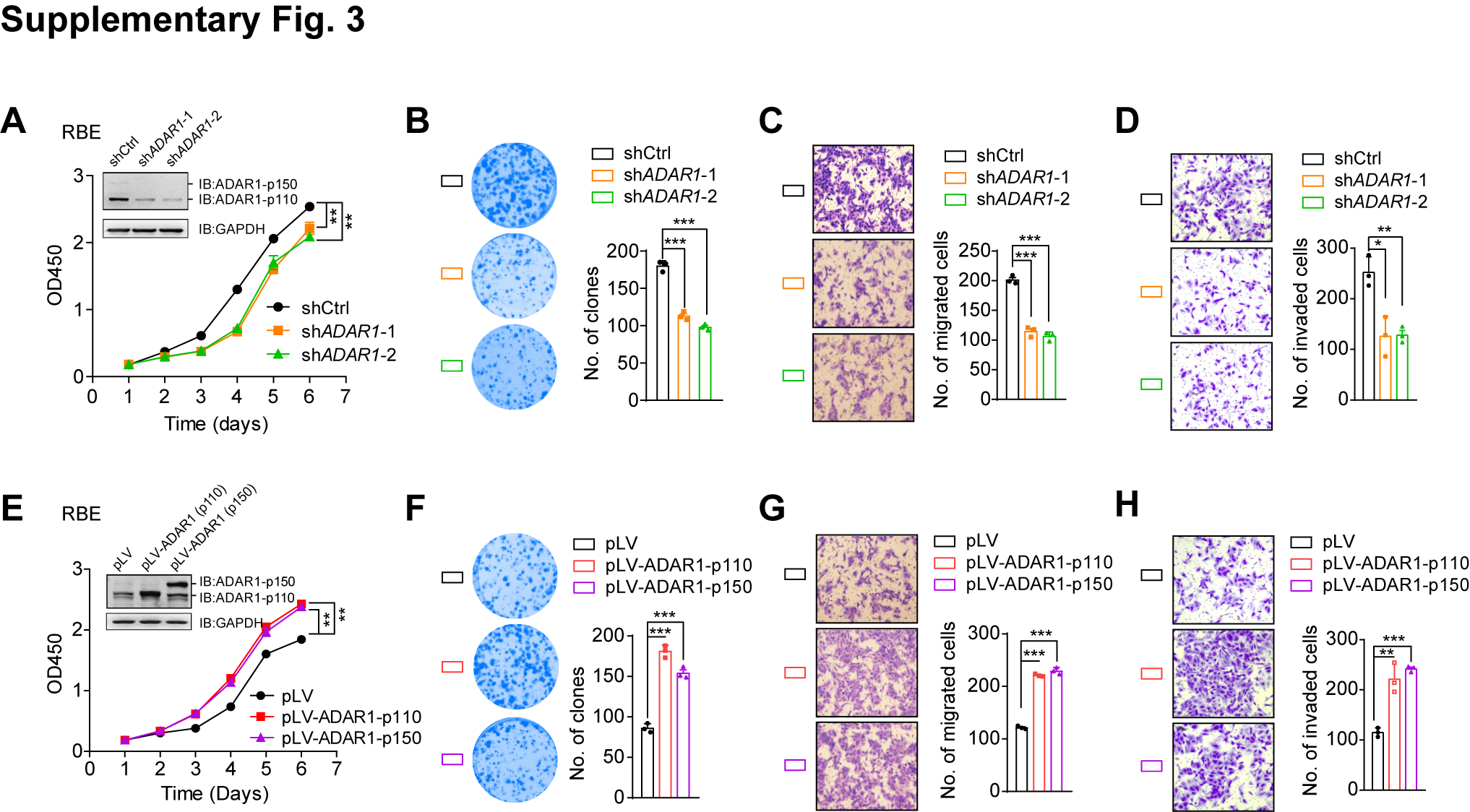


**Supplementary Fig. 3 ADAR1 promotes RBE cells growth, colony formation, migration and invasion.**

**A**-**D** Knockdown of *ADAR1* reduces the abilities of RBE cells growth (**A**), colony formation (**B**), migration (**C**) and invasion (**D**).

**E-H** Overexpression of *ADAR1* promotes the abilities of RBE cells growth (**E**), colony formation (**F**), migration (**G**) and invasion (**H**).

Data are presented as the mean ± standard deviation (s.d.). ^*^*P* < 0.05, ^**^*P* < 0.01 and ^***^*P* < 0.001; assessed by Student’s *t* test.


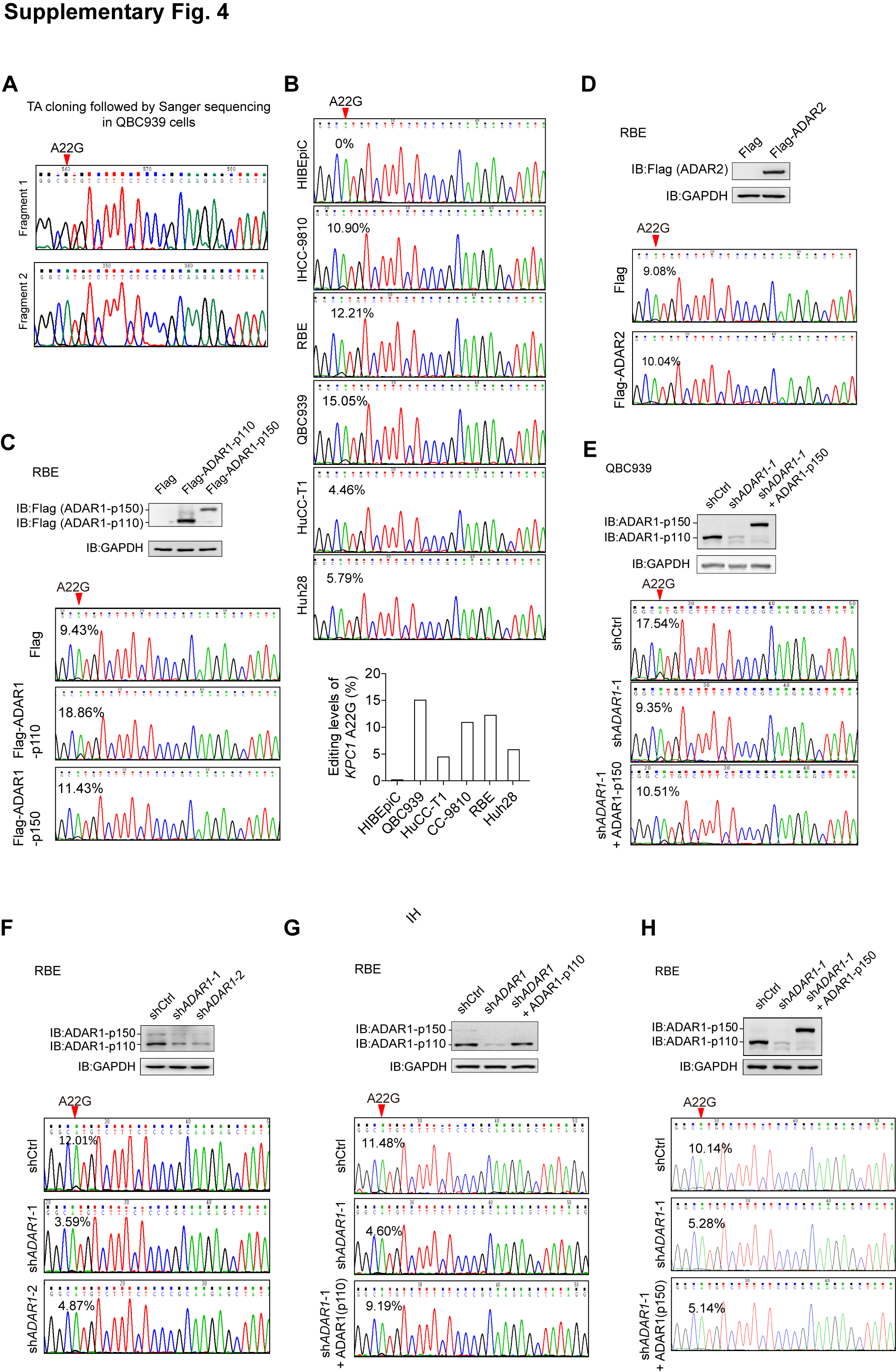


**Supplementary Fig. 4 Over-editing of A22G at *KPC1* mRNA was mediated by ADAR1-p110.**

**A** Chromatograms of the sequence surrounding the *KPC1* mRNA A22G in two TA cloning fragments by Sanger sequencing in QBC939 cells.

**B** The levels of *KPC1* mRNA A22G editing in a panel of cell lines, consisting of one normal intrahepatic biliary epithelial cell line (HIBEpiC) and six types of human iCCA cell line (including IHCC-9810, QBC939, HuCC-T1, RBE and Huh28). The red triangle highlights the double peak A/G, which was labeled with the percentage of edited transcripts assessed by (G/(G + A)).

**C** and **D** The levels of *KPC1* A22G editing in RBE cells upon overexpression of *ADAR1-p110* or -*p150* (**C**) or *ADAR2* (**D**).

**E** The levels of *KPC1* A22G editing in *ADAR1*-knocked-down (by sh*ADAR1*-1) QBC939 cells with enforced expression of *ADAR1-p150*.

**F** The levels of *KPC1* A22G editing in RBE cells upon knockdown of *ADAR1*.

**G** and **H** The levels of *KPC1* A22G editing in *ADAR1*-knocked-down (by sh*ADAR1*-1) RBE cells with enforced expression of *ADAR1-p110* (**G**) or -*p150* (**H**).


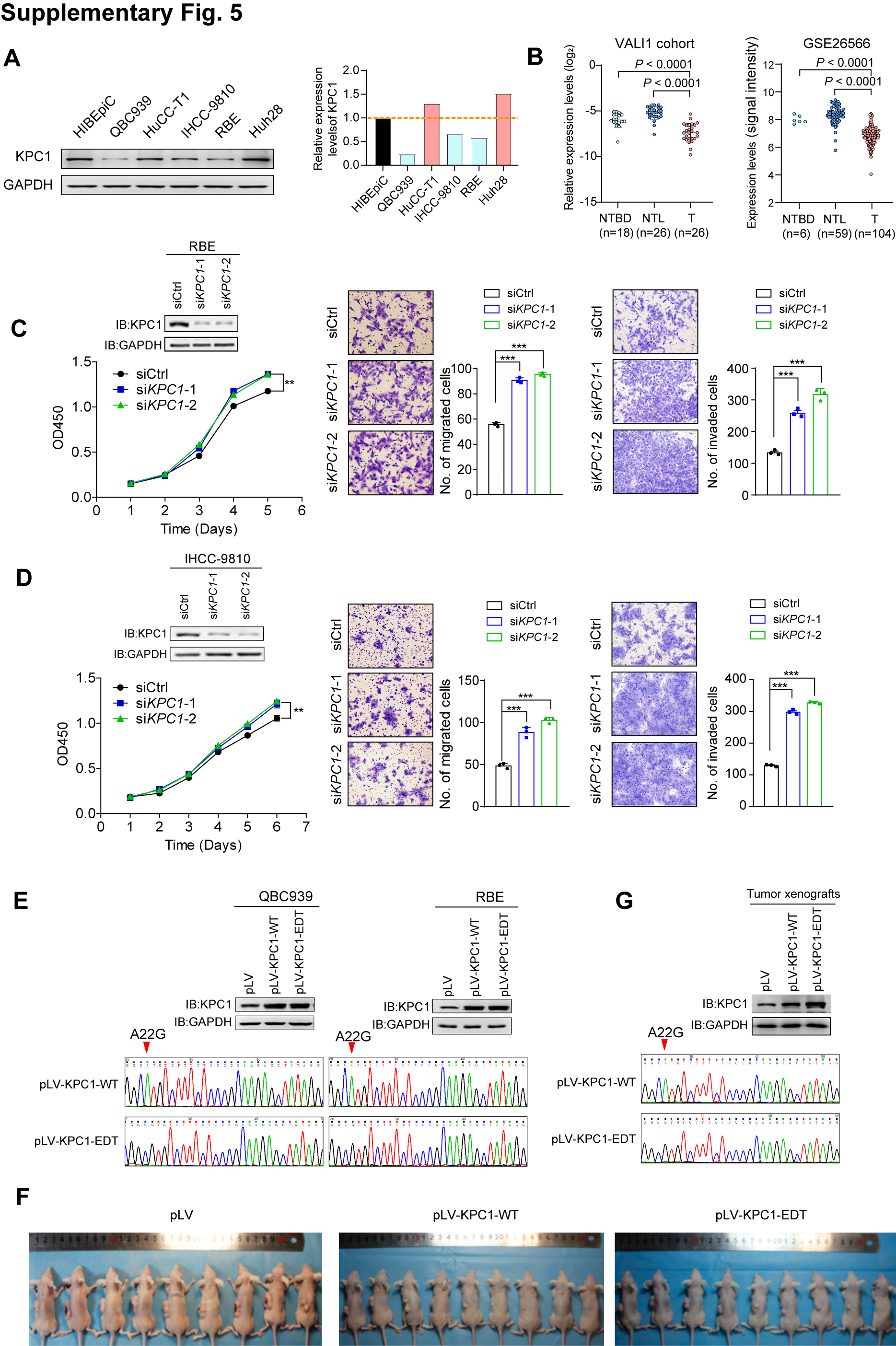


**Supplementary Fig. 5** **Effects of KPC1 and its p.M8V editing on malignant phenotypes of cholangiocarcinoma cells.**

**A** Immunoblotting of KPC1 in a panel of cell lines, consisting of one normal intrahepatic biliary epithelial cell line (HIBEpiC) and six types of human iCCA cell line (including IHCC-9810, QBC939, HuCC-T1, RBE and Huh28).

**B** The mRNA expression levels of *KPC1* in non-tumor bile duct tissues (NTBD), non-tumor liver tissues (NTL) and iCCA tumors (T) from the patients of VALI1 cohort assessed by qRT-PCR assays (left), and the patients of another independent cohort (GSE26566) assessed by RNA-seq (right).

**C** and **D** The effects of knockdown of *KPC1* (by si*KPC1*-1 or si*KPC1*-2) on cells growth, migration and invasion in RBE (**C**) and IHCC-9810 cells (**D**).

**E** Confirmation of overexpression of wide-type KPC1 (KPC1-WT) and edited KPC1 at p.M8V (A22G) (KPC1-EDT) in QBC939 and RBE cells.

**F** The effects of overexpression of KPC1-WT or KPC1-EDT in RBE cells on subcutaneous tumor growth in nude mice (n = 9).

**G** Confirmation of overexpression of KPC1-WT or KPC1-EDT in xenografted tumor tissues from the nude mice implanted with RBE cells.

Data are presented as the mean ± standard deviation (s.d.). ^*^*P* < 0.05, ^**^*P* < 0.01 and ^***^*P* < 0.001; assessed by Student’s *t* test.


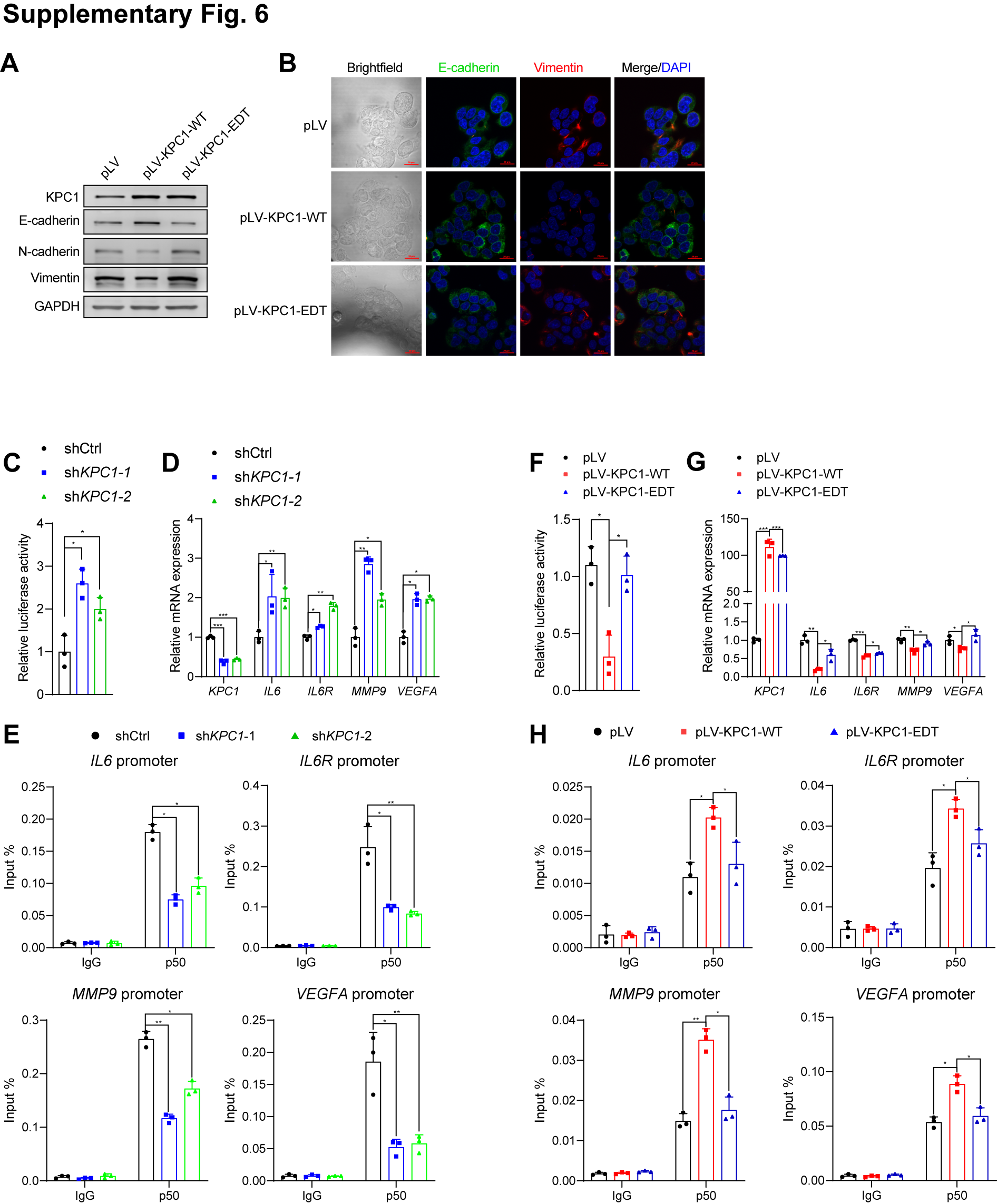


**Supplementary Fig. 6.** **KPC1 p.M8V editing induces inactivation of NF-**κ**B signaling.**

**A** and **B** Immunoblotting (**A**) and immunofluorescence assays (**B**) in QBC939 cells showed that overexpression of wide-type KPC1 (KPC1-WT) inhibits epithelial-mesenchymal transition (EMT) evidenced by epithelial marker E-cadherin and mesenchymal marker N-cadherin or Vimentin, while overexpression of edited KPC1 (KPC1-EDT) abolishes this effect. Scale bar in (**B**), 20 µm.

**C** Luciferase reporter assays showed that knockdown of *KPC1* enhances the NF-κB signaling activity in HEK293T cells.

**D** Quantitative real-time (qRT)-PCR assays in HEK293T cells showed that knockdown of *KPC1* induces the mRNA expression levels of multiple downstream targets of p50, including *IL6*, *IL6R*, *MMP9* and *VEGFA*. The relative expressions of these target genes were normalized to *GAPDH*.

**E** Chromatin immunoprecipitation (ChIP)-qPCR assays in HEK293T cells showed that knockdown of *KPC1* decreased the binding of p50 to the promoters of its targets, including *IL6*, *IL6R*, *MMP9* and *VEGFA*.

**F** Luciferase reporter assays showed that the wide-type KPC1 (KPC1-WT) reduces the NF-κB signaling activity in HEK293T cells; whereas the KPC1 p.M8V editing (KPC1-EDT) abolished this effect.

**G** qRT-PCR assays in HEK293T cells showed that the KPC1-WT reduces the mRNA expression levels of multiple downstream targets of p50, including *IL6*, *IL6R*, *MMP9* and *VEGFA*; whereas the KPC1-EDT abolished these effects. The relative expressions of these target genes were normalized to *GAPDH*.

**H** ChIP-qPCR assays in HEK293T cells showed that the KPC1-WT induces the binding of p50 to the promoters of its targets, including *IL6*, *IL6R*, *MMP9* and *VEGFA*; whereas the KPC1-EDT abolished these effects.

Data are presented as the mean ± standard deviation (s.d.). ^*^*P* < 0.05, ^**^*P* < 0.01 and ^***^*P* < 0.001; assessed by Student’s *t* test.


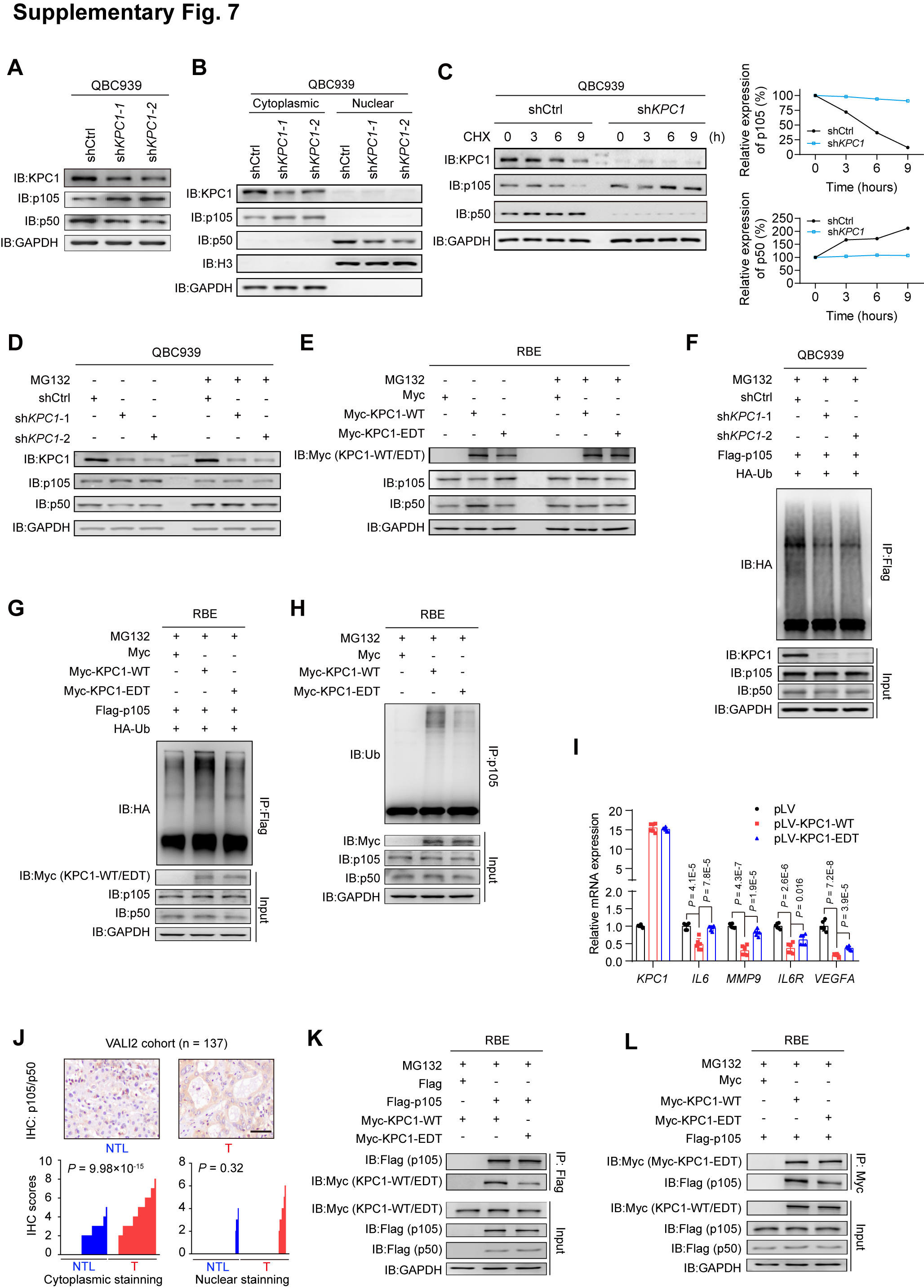


**Supplementary Fig. 7** **KPC1 p.M8V editing reduces NF-**κ**B signaling by attenuating the ubiquitination of p105 and its cleavage to p50.**

**A** The effects of *KPC1* knockdown on p105 and p50 proteins levels in QBC939 cells.

**B** The effects of *KPC1* knockdown on p105 and p50 proteins levels in cytoplasmic and nuclear fractions of QBC939 cells, respectively. Tubulin and histone H3 were used as cytoplasmic and nuclear markers, respectively.

**C** The effects of *KPC1* knockdown on the levels of p105 and p50 proteins in QBC939 cells upon cycloheximide (CHX) treatment (50 μg/mL) at the indicated times. The quantitative results of the p105 and p50 proteins levels were shown in the right panel.

**D** The effects of *KPC1* knockdown on p105 and p50 protein levels in QBC939 cells without or with MG132 treatment (20 μM).

**E** The effects of overexpression of wide-type KPC1 (KPC1-WT) or KPC1 p.M8V editing (KPC1-EDT) on the levels of p105 and p50 proteins in RBE cells without or with MG132 treatment (20 μM).

**F** The effects of *KPC1* knockdown on the ubiquitination of exogenous Flag-p105 in QBC939 cells treated with MG132 (20 µM). Lysates were immunoprecipitated and immunoblotted with antibodies against Flag (total p105) and HA (ubiquitinated p105), respectively.

**G** The effects of overexpression of KPC1-WT or KPC1-EDT on the ubiquitination of exogenous Flag-p105 in RBE cells treated with MG132 (20 µM). Lysates were immunoprecipitated and immunoblotted with antibodies against Flag (total p105) and HA (ubiquitinated p105), respectively.

**H** The effects of overexpression of KPC1-WT or KPC1-EDT on the ubiquitination of endogenous p105 in RBE cells treated with MG132 (20 µM). Lysates were immunoprecipitated and immunoblotted with antibodies against p105 (total p105) and Ub (ubiquitinated p105), respectively.

**I** The effects of overexpression of KPC1-WT or KPC1-EDT on the expression levels of p50 targets in subcutaneous tumors determined by qRT-PCR analyses.

**J** IHC assays for the cytoplasmic or nuclear p105/p50 levels in iCCA tissues from the VALI2 cohort. Upper, the representative IHC images of p105/p50 in tumor (T) and non-tumor liver (NTL) tissues. Lower, the cytoplasmic (left) and nuclear (right) IHC staining scores of p105/p50, respectively. Scale bars, 200 μm. The *P* value was assessed by Wilcox rank sum test.

**K** Co-immunoprecipitation (co-IP) of p105 in RBE cells transfected with the indicated constructs using a Flag-specific antibody. Lysates were immunoprecipitated with antibody against Flag followed by immunoblotting with antibodies to Myc (p105-bounded KPC1-WT or KPC1-EDT) and Flag (p105).

**L** Co-IP of KPC1-WT/EDT in RBE cells transfected with the indicated constructs using a Myc-specific antibody. Lysates were immunoprecipitated with antibody against Myc followed by immunoblotting with antibodies to Flag (KPC1-WT or KPC1-EDT-bounded p105) and Myc (KPC1-WT/EDT).


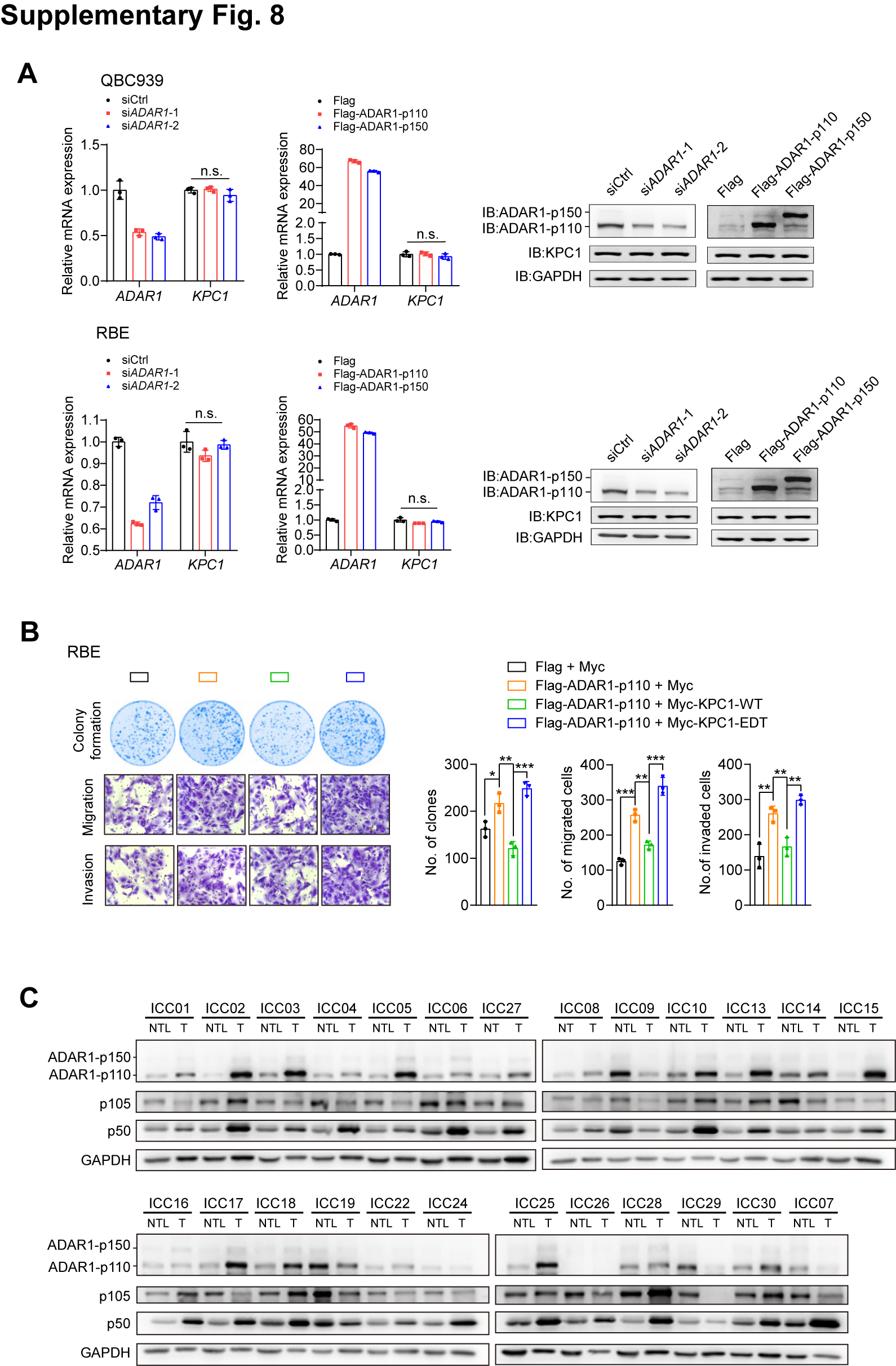


**Supplementary Fig. 8** **ADAR1 exerts its oncogenic role *via* KPC1 p.M8V editing.**

**A** The effects of overexpression or knockdown of *ADAR1* on the mRNA and protein expression levels of KPC1 in QBC939 (upper) and RBE (lower) cells. Left, qRT-PCR assays for assessing the effect of ADAR1 on the mRNA expression levels of *KPC1*. Right, immunoblotting assays for assessing the effect of ADAR1 on the protein expression levels of KPC1.

**B** The effects of overexpression of wide-type KPC1 (KPC1-WT) or KPC1 p.M8V editing (KPC1-EDT) on colony formation, migration and invasion capacities in *ADAR1-p110*-overexpressed RBE cells.

**C** The protein expression levels of ADAR1 (including p110 and p150), p105 and p50 determined by immunoblotting assays in iCCA tissues (T) and adjacent non-tumor liver tissues (NTL) of the patients from the VALI1 cohort (n = 25). Data are presented as the mean ± standard deviation (s.d.). ^*^*P* < 0.05, ^**^*P* < 0.01 and ^***^*P* < 0.001; assessed by Student’s *t* test.


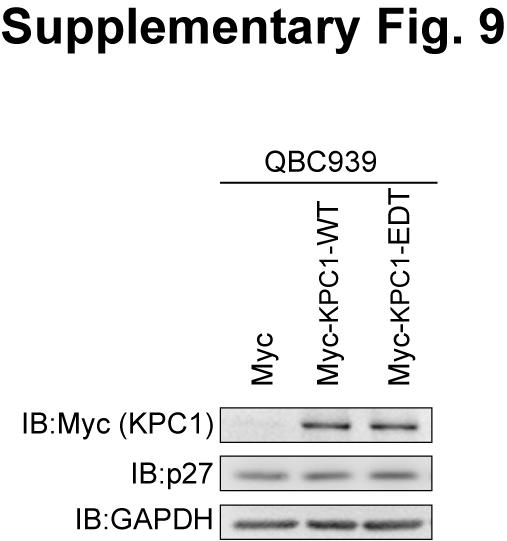


**Supplementary Fig. 9** **KPC1 doesn’t affect the protein levels of p27 in QBC939 cells.**

The effects of overexpression of wide-type KPC1 (KPC1-WT) and edited KPC1 at p.M8V (KPC1-EDT) on p27 protein levels in QBC939 cells.

**Supplementary Table 1. Major demographic and clinicopathological characteristics of iCCA patients in this study.**

| **Variables** | **DISC cohort**  **(n = 15)** | |  | **VALI1 cohort**  **(n = 26)** | |  | **VALI2 cohort**  **(n = 137)** | |
| --- | --- | --- | --- | --- | --- | --- | --- | --- |
|  | **No. of patients** | **%** |  | **No. of patients** | **%** |  | **No. of patients** | **%** |
| Sex |  |  |  |  |  |  |  |  |
| Male | 11 | 73.3 |  | 20 | 76.9 |  | 94 | 68.6 |
| Female | 4 | 26.7 |  | 6 | 23.1 |  | 43 | 31.4 |
| Age, years |  |  |  |  |  |  |  |  |
| Old (> 50) | 8 | 53.3 |  | 9 | 34.6 |  | 78 | 56.9 |
| Young (≤ 50) | 7 | 46.7 |  | 17 | 65.4 |  | 59 | 43.1 |
| HBV^a^ |  |  |  |  |  |  |  |  |
| Positive | 4 | 26.7 |  | 12 | 46.2 |  | 70 | 51.1 |
| Negative | 11 | 73.3 |  | 14 | 53.8 |  | 65 | 47.4 |
| NA | 0 | 0.0 |  | 0 | 0.0 |  | 2 | 1.5 |
| Cirrhosis |  |  |  |  |  |  |  |  |
| Positive | 3 | 20.0 |  | 13 | 50.0 |  | 97 | 70.8 |
| Negative | 12 | 80.0 |  | 11 | 42.3 |  | 40 | 29.2 |
| NA | 0 | 0.0 |  | 2 | 7.7 |  | 0 | 0.0 |
| Tumor Size |  |  |  |  |  |  |  |  |
| Large (> 5 cm) | 12 | 80.0 |  | 15 | 57.7 |  | 84 | 61.3 |
| Small (≤ 5 cm) | 3 | 20.0 |  | 11 | 42.3 |  | 52 | 38.0 |
| NA | 0 | 0.0 |  | 0 | 0.0 |  | 1 | 0.7 |
| Multinodular tumor^b^ |  |  |  |  |  |  |  |  |
| Yes | 6 | 40.0 |  | 14 | 53.8 |  | NA | NA |
| No | 9 | 60.0 |  | 12 | 46.2 |  | NA | NA |
| Histologic grade^c^ |  |  |  |  |  |  |  |  |
| III-IV | 2 | 13.3 |  | 10 | 38.5 |  | 73 | 53.3 |
| I-II | 12 | 80.0 |  | 12 | 46.2 |  | 64 | 46.7 |
| NA | 1 | 6.7 |  | 3 | 11.5 |  | 0 | 0.0 |
| Microvascular invasion |  |  |  |  |  |  |  |  |
| Positive | 3 | 20.0 |  | 6 | 23.1 |  | 51 | 37.2 |
| Negative | 11 | 73.3 |  | 20 | 76.9 |  | 86 | 62.8 |
| NA | 1 | 6.7 |  | 0 | 0.0 |  | 0 | 0.0 |

^a^The status of hepatitis B virus surface antigen (HBsAg). ^b^The status of the primary tumor nodules: Yes, multiple nodules; No, one nodule. ^c^The differentiation status of tumors: I, well differentiated (low grade); II, moderately differentiated (intermediate grade); III, poorly differentiated (high grade); and IV, undifferentiated (high grade).

All three cohorts were recruited from the Eastern Hepatobiliary Surgery Hospital (Shanghai, China). The RNAs extracted from the tissues of the patients from the DISC cohort (recruited between August, 2008 and December, 2012) were used for RNA-seq, and the matched genomic DNAs were used for WES and genome-wide SNP genotyping by Affymetrix SNP Array 6.0, respectively. The genomic DNAs and the RNAs from the tissues of the patients from the VALI1 cohort, which was recruited between August, 2008 and December, 2012, were used for RNA editing validation and mRNA expression levels quantification. The tissue microarrays from the tissues of the patients from the VALI2 cohort, which was recruited between July, 2001 and December, 2005, were used for immunohistochemistry (IHC) assays. NA, not available; DISC, the discovery cohort; VALI1, the validation cohort 1; VALI2, the validation cohort 2.

**Supplementary Table 2. Summary of the RNA-seq data for 15 pairs of iCCA tissues and adjacent non-tumor liver tissues.**

| **Samples** | **Clean reads^a^** | **Left mapped reads** | **Right mapped reads** | **Aligned pairs** | **Mapping rates (%)** | **Coverages (%)** |
| --- | --- | --- | --- | --- | --- | --- |
| ICC010T | 18,043,051 | 17,007,000 | 16,574,185 | 16,074,927 | 88.1 | 76.3 |
| ICC010N | 36,992,640 | 35,061,590 | 33,993,157 | 33,054,241 | 88.3 | 74.2 |
| ICC001T | 21,591,640 | 20,329,512 | 20,239,863 | 19,154,726 | 81.7 | 67.8 |
| ICC011T | 27,438,666 | 25,869,746 | 25,082,324 | 24,324,110 | 87.5 | 63.0 |
| ICC001N | 19,420,300 | 17,978,612 | 17,867,124 | 16,693,671 | 77.4 | 68.2 |
| ICC011N | 19,478,478 | 18,560,035 | 18,024,386 | 17,571,037 | 88.6 | 76.7 |
| ICC002T | 29,108,121 | 27,573,385 | 27,277,843 | 26,051,571 | 86.1 | 78.2 |
| ICC012T | 14,685,065 | 13,885,824 | 13,456,362 | 13,055,730 | 87.6 | 71.4 |
| ICC002N | 30,126,761 | 28,573,420 | 28,143,318 | 27,110,623 | 84.3 | 76.0 |
| ICC012N | 55,588,458 | 52,921,902 | 51,388,246 | 50,061,054 | 88.6 | 73.7 |
| ICC003T | 33,415,372 | 31,301,225 | 30,693,384 | 28,954,192 | 78.4 | 74.4 |
| ICC013T | 24,200,552 | 23,154,276 | 23,109,914 | 22,297,744 | 85.4 | 75.2 |
| ICC003N | 27,558,245 | 25,712,778 | 25,133,431 | 23,850,663 | 73.4 | 67.0 |
| ICC013N | 21,932,421 | 20,946,109 | 20,952,903 | 20,188,234 | 86.2 | 74.5 |
| ICC004T | 23,183,219 | 21,713,860 | 21,492,474 | 20,352,749 | 81.7 | 72.5 |
| ICC014T | 26,734,642 | 25,332,004 | 25,527,302 | 24,352,381 | 86.1 | 77.5 |
| ICC004N | 30,689,483 | 28,958,073 | 28,687,980 | 27,388,320 | 84.0 | 73.6 |
| ICC014N | 28,641,215 | 27,356,939 | 27,385,370 | 26,317,733 | 85.0 | 75.7 |
| ICC005T | 17,733,448 | 16,864,023 | 16,163,408 | 15,496,306 | 82.9 | 76.4 |
| ICC015T | 30,763,968 | 28,976,974 | 28,698,745 | 27,327,352 | 84.9 | 73.4 |
| ICC005N | 41,988,218 | 40,076,113 | 38,705,843 | 37,232,184 | 84.7 | 75.1 |
| ICC015N | 28,908,443 | 27,758,357 | 27,550,432 | 26,621,581 | 87.9 | 75.0 |
| ICC006T | 23,656,625 | 22,596,337 | 22,351,625 | 21,508,223 | 88.3 | 76.6 |
| ICC006N | 18,936,034 | 17,900,190 | 17,252,461 | 16,446,406 | 82.5 | 76.4 |
| ICC007T | 27,943,957 | 26,535,772 | 25,725,419 | 24,995,977 | 88.1 | 75.1 |
| ICC007N | 34,372,231 | 32,747,317 | 31,759,312 | 30,974,743 | 88.6 | 74.8 |
| ICC008T | 21,163,381 | 20,286,869 | 20,226,472 | 19,509,012 | 86.2 | 77.1 |
| ICC008N | 22,408,791 | 21,272,433 | 21,108,479 | 20,301,681 | 85.2 | 76.6 |
| ICC009T | 26,677,585 | 25,649,937 | 25,568,485 | 24,744,265 | 87.6 | 74.4 |
| ICC009N | 23,960,152 | 23,060,454 | 22,768,439 | 22,101,629 | 86.7 | 76.0 |
| **Average** | **26,911,372** | **25,532,036** | **25,096,956** | **24,137,102** | **85.1** | **74.1** |

^a^Reads that passed the filtering of rRNAs, adapters and low-quality reads.

The sequencing reads were mapped to the human genome (hg19 build) and UCSC annotated genes with at most 2 mismatches by Bowtie2.

**Supplementary** Table 3. Summary of whole-exome sequencing for 15 pairs of iCCA tissues and adjacent non-tumor liver tissues.

| **Samples** | **Depth (×)** | **Mapped bases at the indicated depth (%)** | | | | |  |
| --- | --- | --- | --- | --- | --- | --- | --- |
|  |  | **≥ 1 ×** | **≥ 4 ×** | **≥ 10 ×** | **≥ 20 ×** | **≥ 50 ×** |  |
| ICC001T | 89.7 | 99.9 | 99.7 | 98.8 | 95.3 | 71.1 |  |
| ICC001N | 94.9 | 100.0 | 99.8 | 99.1 | 96.6 | 76.4 |  |
| ICC002T | 91.1 | 99.8 | 99.5 | 98.7 | 95.6 | 73.2 |  |
| ICC002N | 107.9 | 99.9 | 99.7 | 99.2 | 97.4 | 81.9 |  |
| ICC003T | 91.2 | 99.9 | 99.7 | 98.8 | 95.3 | 71.3 |  |
| ICC003N | 77.6 | 99.9 | 99.7 | 98.7 | 94.7 | 66.3 |  |
| ICC004T | 99.6 | 100.0 | 99.8 | 99.1 | 96.6 | 77.2 |  |
| ICC004N | 84.7 | 100.0 | 99.8 | 98.9 | 95.6 | 70.7 |  |
| ICC005T | 86.2 | 99.8 | 99.5 | 98.6 | 95.4 | 71.5 |  |
| ICC005N | 81.4 | 99.8 | 99.5 | 98.6 | 95.2 | 69.6 |  |
| ICC006T | 90.7 | 99.9 | 99.7 | 98.9 | 95.8 | 73.1 |  |
| ICC006N | 109.1 | 99.9 | 99.8 | 99.2 | 97.4 | 82.3 |  |
| ICC007T | 90.5 | 99.9 | 99.7 | 98.8 | 95.7 | 73.3 |  |
| ICC007N | 87.2 | 99.9 | 99.7 | 98.9 | 95.9 | 72.8 |  |
| ICC008T | 87.7 | 99.8 | 99.6 | 98.6 | 95.1 | 71.0 |  |
| ICC008N | 87.4 | 99.8 | 99.6 | 98.8 | 95.9 | 72.7 |  |
| ICC009T | 94.7 | 99.9 | 99.7 | 98.8 | 95.6 | 73.4 |  |
| ICC009N | 94.1 | 99.9 | 99.7 | 98.8 | 95.9 | 75.3 |  |
| ICC010T | 109.6 | 99.9 | 99.6 | 99.0 | 97.1 | 82.0 |  |
| ICC010N | 106.2 | 99.9 | 99.6 | 98.9 | 96.8 | 81.0 |  |
| ICC011T | 82.7 | 99.9 | 99.6 | 98.4 | 94.3 | 67.4 |  |
| ICC011N | 82.7 | 99.9 | 99.6 | 98.6 | 94.9 | 69.6 |  |
| ICC012T | 96.1 | 99.9 | 99.7 | 98.9 | 96.2 | 75.8 |  |
| ICC012N | 88.2 | 99.9 | 99.6 | 98.5 | 95.1 | 71.6 |  |
| ICC013T | 78.9 | 99.9 | 99.5 | 97.9 | 92.6 | 63.0 |  |
| ICC013N | 83.0 | 99.9 | 99.6 | 98.5 | 94.9 | 69.6 |  |
| ICC014T | 103.0 | 99.9 | 99.7 | 98.9 | 96.6 | 79.0 |  |
| ICC014N | 96.9 | 99.9 | 99.7 | 98.9 | 96.3 | 76.9 |  |
| ICC015T | 99.4 | 99.9 | 99.7 | 99.0 | 96.5 | 77.8 |  |
| ICC015N | 108.0 | 99.9 | 99.7 | 99.1 | 97.0 | 81.3 |  |
| **Average** | **92.7** | **99.9** | **99.7** | **98.8** | **95.8** | **73.9** |  |

To obtain high-quality data, we removed the low-quality reads with more than five unknown bases and then aligned the remaining reads to the human genome (hg19 build) using BWA (v0.5.9) with default parameters. Duplication rate is the fraction of duplicated reads in raw data. Picard tool (v1.55) was used to detect and remove the PCR duplicates. Sequencing depth and coverage were calculated based on the high-quality data.

**Supplementary Table 4. T**he putative A-to-I editing sites in protein-coding regions.

| **ID** | **Positions^a^** | **No. of**  **samples^b^** | **Located within**  **Alu element** | **Documented**  **in databases** | **Genes** | **Annotation^c^** | **NS/S** | **PhyloP^d^** | **Validation** | **Functional relevance (PMID)** |
| --- | --- | --- | --- | --- | --- | --- | --- | --- | --- | --- |
| 1 | chr1:225974614 | 27 | Yes | DARNED, RADAR | *SRP9* | NM_001130440:exon3:  c.A192G:p.I64M | NS | 0.26 | TRUE | 19812674, 26439496 |
| 2 | chr3:58141791 | 20 | No | DARNED | *FLNB* | NM_001457:exon41:  c.A6877G:p.M2293V | NS | 1.52 | TRUE | 24302582 |
| 3 | chr3:49728642 | 17 | No |  | *KPC1 (RNF123)* | NM_022064:exon2:  c.A44G:p.Y15C | NS | 6.25 | FALSE |  |
| 4 | chr2:201750058 | 15 | Yes | DARNED, RADAR | *PPIL3* | NM_032472:exon4:  c.A111G:p.V37V | S |  | TRUE |  |
| 5 | chr8:103841636 | 15 | No | DARNED, RADAR | *AZIN1* | NM_148174:exon11:  c.A1099G:p.S367G | NS | 3.7 | TRUE | 23291631 |
| 6 | chr13:46090371 | 13 | No | DARNED, RADAR | *COG3* | NM_031431:exon17:  c.A1903G:p.I635V | NS | 8.89 | TRUE | 19812674 |
| 7 | chr3:49728620 | 11 | No |  | ***KPC1 (RNF123)*** | NM_022064:exon2:  c.A22G:p.M8V | NS | 1.25 | TRUE |  |
| 8 | chr15:75646087 | 10 | No | DARNED, RADAR | *NEIL1* | NM_024608:exon6:  c.A726G:p.K242K | S |  | TRUE |  |
| 9 | chr21:34923319 | 8 | No | DARNED, RADAR | *SON* | NM_138927:exon3:  c.A1782G:p.L594L | S |  | TRUE |  |
| 10 | chr15:75646086 | 7 | No | DARNED, RADAR | *NEIL1* | NM_024608:exon6:  c.A725G:p.K242R | NS | 6.04 | TRUE | 30061158, 21068368 |
| 11 | chr1:247263717 | 6 | Yes | RADAR | *ZNF669* | NM_001142572:exon4:  c.A1096G:p.N366D | NS | 1.01 | TRUE |  |
| 12 | chr7:99091155 | 5 | Yes | RADAR | *ZNF394* | NM_032164:exon3:  c.A1683G:p.L561L | S |  | TRUE |  |
| 13 | chr10:126451032 | 5 | Yes | DARNED, RADAR | *METTL10* | NM_212554:exon6:  c.A712G:p.T238A | NS | -1.54 | TRUE |  |
| 14 | chr2:201749994 | 4 | Yes | DARNED, RADAR | *PPIL3* | NM_032472:exon4:  c.A175G:p.S59G | NS | 0.25 | TRUE |  |
| 15 | chr11:66523904 | 4 | Yes | DARNED, RADAR | *C11orf80* | NM_024650:exon3:  c.A397G:p.S133G | NS | 0.33 | TRUE |  |
| 16 | chr19:18182130 | 4 | Yes | DARNED, RADAR | *IL12RB1* | NM_153701:exon10:  c.A1035G:p.S345S | S |  | TRUE |  |
| 17 | chr4:190873406 | 3 | No |  | *FRG1* | NM_004477:exon3:  c.A223G:p.N75D | NS | 8.48 | FALSE |  |
| 18 | chr6:5187158 | 3 | Yes | RADAR | *LYRM4* | NM_001164840:exon3:  c.A264G:p.L88L | S |  | TRUE |  |
| 19 | chr12:56629409 | 3 | No |  | ***SLC39A5*** | NM_001135195:exon6:  c.A870G:p.S290S | S |  | TRUE |  |
| 20 | chr19:3595042 | 3 | Yes | RADAR | *TBXA2R* | NM_201636:exon4:  c.A1016G:p.Y339C | NS | -0.72 | TRUE |  |
| 21 | chr19:14593693 | 3 | No | DARNED, RADAR | *GIPC1* | NM_202470:exon2:  c.A96G:p.P32P | S |  | TRUE |  |
| 22 | chr7:131195075 | 2 | Yes | RADAR | *PODXL* | NM_001018111:exon3:  c.A722G:p.H241R | NS | 0.15 | TRUE | 27373511 |
| 23 | chr10:126451098 | 2 | Yes | RADAR | *METTL10* | NM_212554:exon6:  c.A646G:p.T216A | NS | -0.13 | TRUE |  |
| 24 | chr12:49718012 | 2 | Yes | DARNED, RADAR | *TROAP* | NM_001100620:exon4:  c.A412G:p.R138G | NS | -0.17 | TRUE |  |
| 25 | chr14:102551278 | 2 | No |  | *HSP90AA1* | NM_005348:exon5:  c.A721G:p.K241E | NS | 2.25 | FALSE |  |
| 26 | chr16:77770615 | 2 | Yes |  | ***NUDT7*** | NM_001243657:exon4:  cA417G:p.S139S | S |  | TRUE |  |
| 27 | chr19:18182106 | 2 | Yes | DARNED, RADAR | *IL12RB1* | NM_153701:exon10:  c.A1059G:p.P353P | S |  | TRUE |  |
| 28 | chr19:21301135 | 2 | Yes | RADAR | *ZNF714* | NM_182515:exon5:  c.A1665G:p.X555X | S |  | TRUE |  |
| 29 | chrX:1422222 | 2 | Yes |  | *CSF2RA* | NM_001161530:exon9:  c.A1015G:p.R339G | NS | -0.52 | FAILED |  |

^a^The sequencing reads were mapped to the human genome (hg19 build). ^b^The number of samples that support the existence of the putative editing event(s). ^c^Annotation of editing event as follows: gene name:transcript name:exon number:base substitution:amino acid change. ^d^Conservations of editing sites that were predicted using PhyloP.

Chr., chromosome; Ref., reference allele; Var., variation allele; DARNED, a DAtabase of RNa EDiting in humans (https://darned.ucc.ie/); RADAR, a Rigorously Annotated Database of A-to-I RNA editing (http://RNAedit.com); NS, nonsynonymous; S, synonymous.

**Supplementary** Table 5. Comparisons of pathological parameters between the groups of "high" and "low" ADAR1 expression in iCCAs from the validation cohort 2.

| **Variables** | **IHC score for ADAR1** | | | | | ***P*^a^** |
| --- | --- | --- | --- | --- | --- | --- |
|  | **High (n = 74)** | |  | **Low (n = 63)** | |  |
|  | **No. of patients** | **%** |  | **No. of patients** | **%** |  |
| Sex |  |  |  |  |  |  |
| Male | 54 | 73.0 |  | 40 | 63.5 | 0.23 |
| Female | 20 | 27.0 |  | 23 | 36.5 |  |
| Age, years |  |  |  |  |  |  |
| Old (> 50) | 39 | 52.7 |  | 39 | 61.9 | 0.28 |
| Young (≤ 50) | 35 | 47.3 |  | 24 | 38.1 |  |
| AFP, ng/mL |  |  |  |  |  |  |
| High (> 20 ) | 23 | 31.1 |  | 14 | 22.2 | 0.24 |
| Low (≤ 20) | 51 | 68.9 |  | 49 | 77.8 |  |
| Cirrhosis |  |  |  |  |  |  |
| Positive | 48 | 64.9 |  | 49 | 77.8 | 0.10 |
| Negtve | 26 | 35.1 |  | 14 | 22.2 |  |
| Tumor size, cm |  |  |  |  |  |  |
| > 5 | 47 | 63.5 |  | 37 | 58.7 | 0.65 |
| ≤ 5 | 27 | 36.5 |  | 25 | 39.7 |  |
| NA | 0 | 0 |  | 1 | 1.6 |  |
| PVTT |  |  |  |  |  |  |
| Positive | 17 | 23.0 |  | 10 | 15.9 | 0.26 |
| Negtive | 56 | 75.7 |  | 53 | 84.1 |  |
| NA | 1 | 1.3 |  | 0 | 0 |  |
| Histologic grade^b^ |  |  |  |  |  |  |
| III-IV | 36 | 48.7 |  | 37 | 58.7 | 0.32 |
| I-II | 38 | 51.3 |  | 26 | 41.3 |  |

^a^*P* values were caculated by *χ*^2^ test. *P* < 0.05 was considered to be statistically significant. ^b^The differentiation status of tumors: I, well differentiated (low grade); II, moderately differentiated (intermediate grade); III, poorly differentiated (high grade); and IV, undifferentiated (high grade).

The immunohistochemistry score for ADAR1 greater than 6 were considered as “High” expression (n = 74), whereas the others were “Low” expression (n = 63). ADAR1, adenosine deaminase acting on RNA 1. AFP, alpha-fetoprotein; NA, not available; PVTT, portal vein tumor thrombus.

**Supplementary** Table 6. Cox hazard ratios for overall survival and disease-free survival rates based on ADAR1 expression levels in iCCAs from the validation cohort 2.

| **Pathological parameters** | **For overall survival rate** | | | | |  | **For disease-free survival rate** | | | | |
| --- | --- | --- | --- | --- | --- | --- | --- | --- | --- | --- | --- |
|  | **Univariateanalyses^a^** | |  | **Multivariate analyses^b^** | |  | **Univariate analyses^a^** | |  | **Multivariate analyses^b^** | |
|  | **HR**  **(95% CI)** | ***P*** |  | **HR**  **(95% CI)** | ***P*** |  | **HR**  **(95% CI)** | ***P*** |  | **HR**  **(95% CI)** | ***P*** |
| Age (> 50 years old) | 0.81 (0.58-1.14) | 0.24 |  | - | - |  | 0.81 (0.58-1.14) | 0.23 |  | - | - |
| Gender (male) | 1.13 (0.79-1.63) | 0.51 |  | - | - |  | 1.22 (0.84-1.75) | 0.30 |  | - | - |
| AFP (> 20 ng/mL) | 1.06 (0.72-1.55) | 0.77 |  | - | - |  | 1.09 (0.74-1.59) | 0.67 |  | - | - |
| Cirrhosis | 1.01 (0.70-1.46) | 0.97 |  | - | - |  | 0.98 (0.68-1.42) | 0.91 |  | - | - |
| Tumor size (> 5 cm) | 1.26 (0.89-1.79) | 0.19 |  | - | - |  | 1.34 (0.94-1.90) | 0.10 |  | - | - |
| PVTT | **1.57 (1.02-2.41)** | **0.040** |  | **1.53 (0.99-2.35)** | **0.054** |  | **1.73 (1.12-2.66)** | **0.013** |  | **1.76 (1.14-2.70)** | **0.010** |
| Histologic grade (III-IV) | 1.13 (0.80-1.58) | 0.48 |  | - | - |  | 1.20 (0.85-1.68) | 0.29 |  | - | - |
| ADAR1 expression levels (IHC score > 6) | **1.49 (1.06-2.09)** | **0.021** |  | **1.47 (1.04-2.06)** | **0.027** |  | **1.53 (1.09-2.15)** | **0.014** |  | **1.55 (1.10-2.18)** | **0.012** |

^a^Univariate analysis was performed by using univariate Cox hazard ratio regression for each indicated parameters. ^b^Multivariate analysis was performed by using multivariate Cox hazard ratio regression for PVTT and ADAR1 expression levels.

AFP, alpha-fetoprotein; CI, confidence interval; HR, hazard ratio; IHC, immunohistochemistry; PVTT, portal vein tumor thrombus; -, the parameters, which were not significant for univariate analyses (*P* > 0.05), were not included for multivariate analyses any more.

**Supplementary Table 7. GSEA based on the *KPC1*** expression levels in iCCAs from the DISC cohort.

| **Gene sets** | **ES** | **NES** | ***P*** | **FDR *q*-value** |
| --- | --- | --- | --- | --- |
| HALLMARK_EPITHELIAL_MESENCHYMAL_TRANSITION | -0.549 | -2.705 | 0.0000 | 0.0000 |
| HALLMARK_ESTROGEN_RESPONSE_EARLY | -0.371 | -1.805 | 0.0000 | 0.0021 |
| **HALLMARK_TNFA_SIGNALING_VIA_NFKB** | -0.361 | -1.783 | 0.0000 | 0.0022 |
| HALLMARK_KRAS_SIGNALING_UP | -0.381 | -1.845 | 0.0000 | 0.0023 |
| HALLMARK_TGF_BETA_SIGNALING | -0.470 | -1.857 | 0.0000 | 0.0028 |
| HALLMARK_PROTEIN_SECRETION | -0.395 | -1.733 | 0.0000 | 0.0050 |
| HALLMARK_HYPOXIA | -0.339 | -1.688 | 0.0000 | 0.0054 |
| HALLMARK_NOTCH_SIGNALING | -0.493 | -1.706 | 0.0071 | 0.0054 |
| HALLMARK_ANGIOGENESIS | -0.479 | -1.691 | 0.0025 | 0.0057 |
| HALLMARK_APICAL_JUNCTION | -0.345 | -1.635 | 0.0028 | 0.0074 |
| HALLMARK_P53_PATHWAY | -0.336 | -1.644 | 0.0000 | 0.0077 |
| HALLMARK_MYOGENESIS | -0.339 | -1.613 | 0.0026 | 0.0081 |
| HALLMARK_APOPTOSIS | -0.328 | -1.577 | 0.0000 | 0.0116 |
| HALLMARK_APICAL_SURFACE | -0.423 | -1.489 | 0.0545 | 0.0242 |
| HALLMARK_HEDGEHOG_SIGNALING | -0.439 | -1.469 | 0.0400 | 0.0255 |
| HALLMARK_ESTROGEN_RESPONSE_LATE | -0.301 | -1.449 | 0.0083 | 0.0282 |
| HALLMARK_UV_RESPONSE_DN | -0.307 | -1.416 | 0.0121 | 0.0341 |
| HALLMARK_UNFOLDED_PROTEIN_RESPONSE | -0.308 | -1.385 | 0.0239 | 0.0427 |
| HALLMARK_KRAS_SIGNALING_DN | 0.367 | 1.440 | 0.0273 | 0.0624 |
| HALLMARK_ADIPOGENESIS | 0.327 | 1.523 | 0.0000 | 0.0301 |
| HALLMARK_PEROXISOME | 0.381 | 1.573 | 0.0017 | 0.0226 |
| HALLMARK_CHOLESTEROL_HOMEOSTASIS | 0.438 | 1.753 | 0.0000 | 0.0041 |
| HALLMARK_XENOBIOTIC_METABOLISM | 0.429 | 1.999 | 0.0000 | 0.0000 |
| HALLMARK_BILE_ACID_METABOLISM | 0.474 | 2.002 | 0.0000 | 0.0000 |
| HALLMARK_FATTY_ACID_METABOLISM | 0.497 | 2.232 | 0.0000 | 0.0000 |

The transcriptome was profiled by RNA-seq in iCCAs from the DISC cohort (n = 15). The GSEA was performed based on the median of *KPC1* expression levels. The RNA-seq data has been deposited in NCBI’s GEO under accession number GSE119336. GSEA, gene set enrichment analysis; ES, enrichment score; NES, normalized enrichment score; FDR, false discovery rate; iCCAs, intrahepatic cholangiocarcinomas; DISC cohort, discovery cohort.

**Supplementary Table 8. Primers, siRNAs and shRNAs in this stu**dy.

| **Names** | **Sequences (5’→3’)** |
| --- | --- |
| **Primers for qRT-PCR assays** | |
| ADAR1-F | CCCTTCAGCCACATCCTTC |
| ADAR1-R | GCCATCTGCTTTGCCACTT |
| ADAR2-F | CTGACACGCTCTTCAATGGTT |
| ADAR2-R | GGCGCAGTTCGTTCAAGAT |
| IL6-F | CTCCTTCTCCACAAGCGCC |
| IL6-R | GATGCCGTCGAGGATGTACC |
| IL6R-F | TCACTGTGTCATCCACGACG |
| IL6R-R | CTGGATTCTGTCCAAGGCGT |
| MMP9-F | ACGACGTCTTCCAGTACCGA |
| MMP9-R | TTGGTCCACCTGGTTCAACT |
| VEGFA-F | GAGCTCATGGACGGGTGAG |
| VEGFA-R | CTGGGACCACTTGGCATGG |
| β-actin-F | AGAGCCTCGCCTTTGCCGAT |
| β-actin-R | CCATCACGCCCTGGTGCCT |
| GAPDH-F | CGGAGTCAACGGATTTGGTCGT |
| GAPDH-R | TCTCAGCCTTGACGGTGCCA |
| **Primers for ChIP-qPCR assays** | |
| IL6-F | TAAGGTTTCCAATCAGCCCCA |
| IL6-R | AGAGCTTCTCTTTCGTTCCCG |
| IL6R-F | CAGCGCGAGTTCCTCAAATG |
| IL6R-R | CTACACACACTGCGAGTCCC |
| MMP9-F | CCTGAAGATTCAGCCTGCGG |
| MMP9-R | CCCTGACAGCCTTCTTTGACT |
| VEGFA-F | CAGGCTTCACTGAGCGTCC |
| VEGFA-R | CCGCTACCAGCCGACTTTT |
| **Primers for PCR and Sanger sequencing** | |
| KPC1-gDNA-F | GAGGTTGGGCGGATGTTG |
| KPC1-gDNA-R | TGCGGTTCAGGTAGTCATTCAG |
| KPC1-cDNA-F | ATGGCATCCAAGGGG |
| KPC1-cDNA-R | TTTGTACACGCATGTG |
| ZNF669-gDNA-F | GCCTTCAGCTGTTCCACTTC |
| ZNF669-gDNA-R | TGCCCGGCATTATTTTATTT |
| ZNF669-cDNA-F | GCCTTCAGCTGTTCCACTTC |
| ZNF669-cDNA-R | TGCCCGGCATTATTTTATTT |
| FRG1-gDNA-F | GCAAGATTTAATCGAGAC |
| FRG1-gDNA-R | CTTTGGAAAA TGTTTGAG |
| FRG1-cDNA-F | AGAAGAAGATGAAGAAACCCAG |
| FRG1-cDNA-R | ACGCCCAACAACAAGTCC |
| HSP90AA1-gDNA-F | AAAAGTTCCTCGGGCTAT |
| HSP90AA1-gDNA-R | TTCCCAGTCA TTGGTCAAG |
| HSP90AA1-cDNA-F | GAACCTATGGGTCGTGGAA |
| HSP90AA1-cDNA-R | ATCGTCGGGA TTTCTGGT |
| CSF2RA-gDNA-F | GCCTCCTGAGTAGCTGGGATT |
| CSF2RA-gDNA-R7 | ATGGTGGTGGGTGCCTGT |
| CSF2RA-cDNA-F | TGTCGTACCTGGACTTTCA |
| CSF2RA-cDNA-R | CTCGTCTTCC ACCTCATG |
| SLC39A5-gDNA-F | GGGATGTTGTTGGGTATAGGG |
| SLC39A5-gDNA-R | AGCGATTCTC CTGCCTCA |
| SLC39A5-cDNA-F | TGGGACCTCGTCTACTACGG |
| SLC39A5-cDNA-R | TGGTGGGTGC TGGCTGTTC |
| NUDT7-gDNA-F | GTCTCGCTATGTTGATGA |
| NUDT7-gDNA-R | ACCCCTTCTAAAGTCAGT |
| NUDT7-cDNA-F | CCTGGAGGTAAGCGTGAC |
| NUDT7-cDNA-R | TGGTCTATTAAACCCACAAA |
| **shRNAs and siRNAs** | |
| ADAR1 siRNA-1 | GCATCTGACCCGTGCTATT |
| ADAR1 siRNA-2 | GCTTCAACACTCTGACTAA |
| ADAR2 siRNA-1 | CAGGCACAGATGTTAAAGA |
| ADAR2 siRNA-2 | CCGCTATTGAGGTCATCAA |
| KCP1 siRNA-1 | GGAATGTGACCACAACGAA |
| KPC1 siRNA-2 | GGAGCCAATACGAGTACTA |
| Control siRNA | GUGCGAGGGGGUUGUAAUCTT |
| ADAR1 shRNA-1 | CCTGTGGAATCCAGTGACATTGTGCCTAC |
| ADAR1 shRNA-2 | AGACTCCGTACCATGTCCTGTAGTGACAA |
| Control shRNA | UUCUCCGAACGUGUCACGU |

E, extended primer; F, forward primer; R, reverse primer; qRT-PCR, quantitative real-time polymerase chain reaction; ChIP-qPCR, chromatin immunoprecipitation-quantitative polymerase chain reaction.
