# Supplementary material for: The A-to-I editing of KPC1 promotes intrahepatic cholangiocarcinoma by attenuating proteasomal processing of NF-κB1 p105 to p50
Source: J Exp Clin Cancer Res. 2022 Dec 8;41:338. doi: 10.1186/s13046-022-02549-1 (PMC9730630; doi:10.1186/s13046-022-02549-1)

Figure1

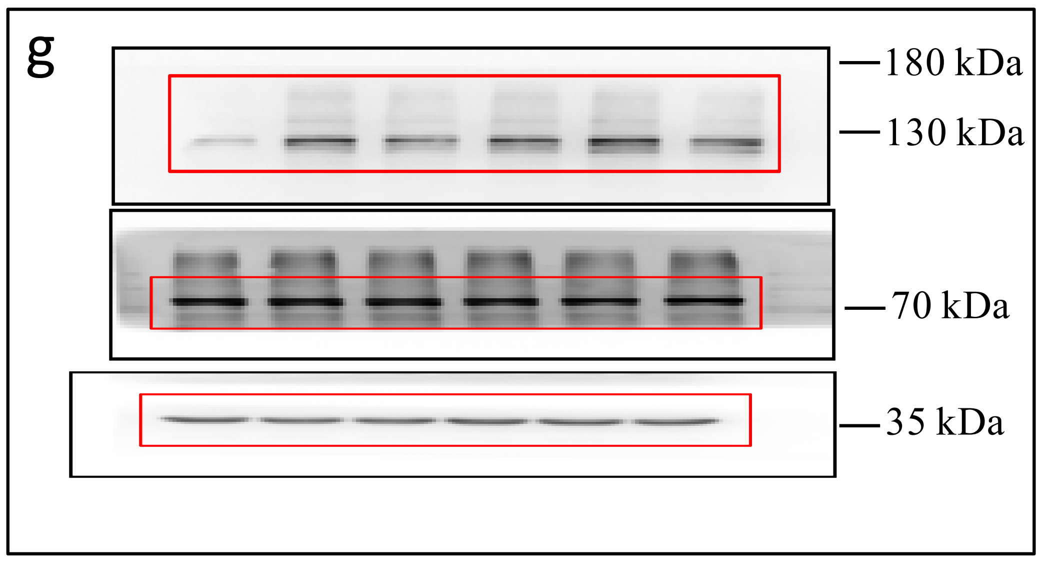

Figure2

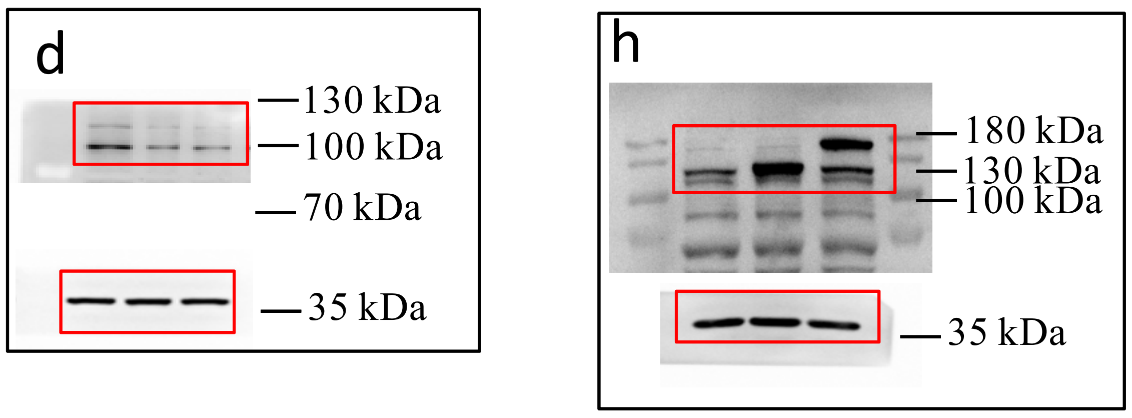

Figure3

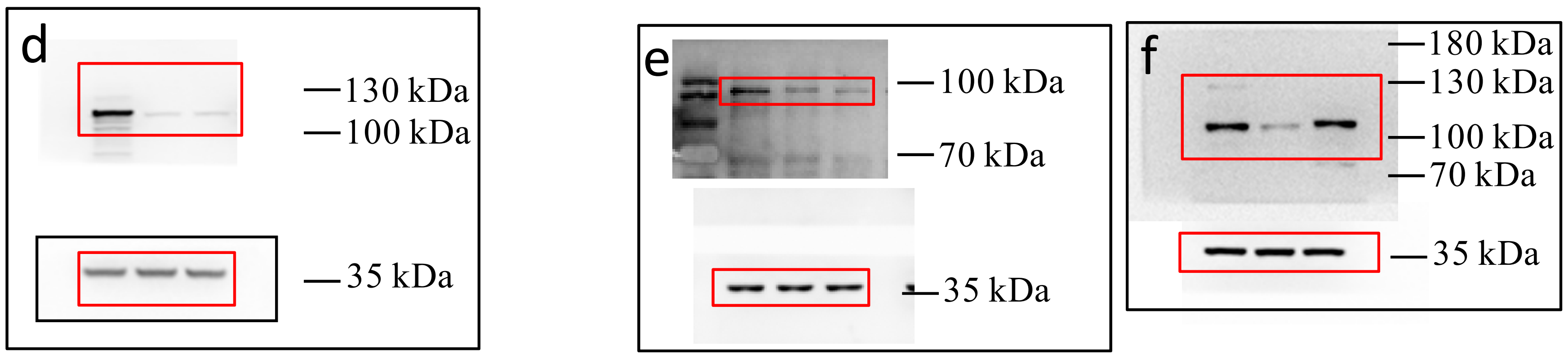

Figure5

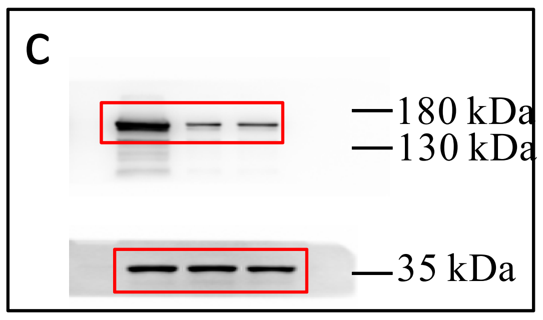

Figure6

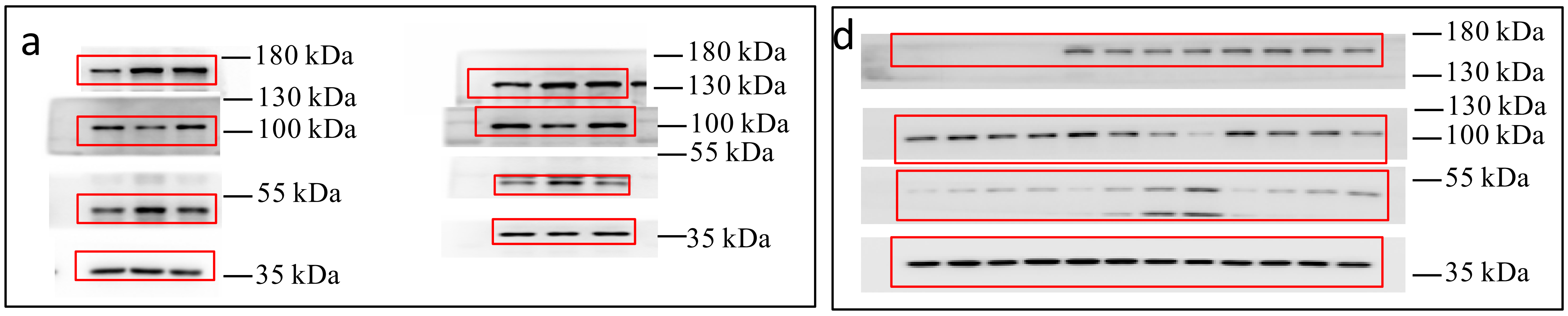

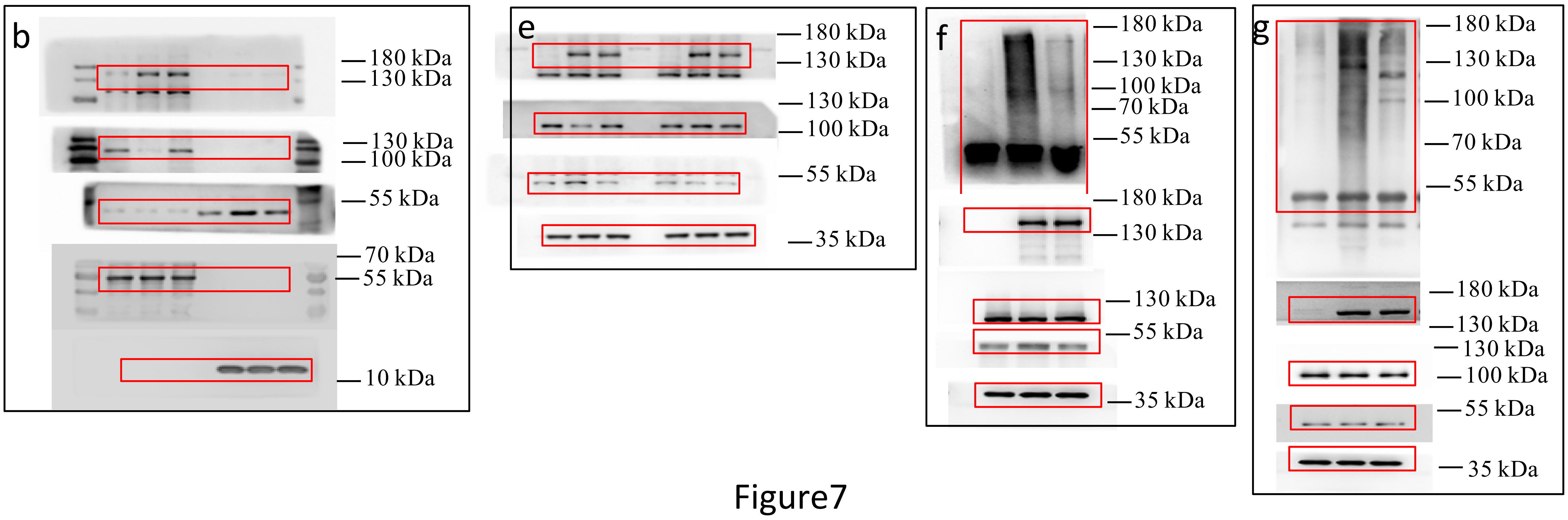

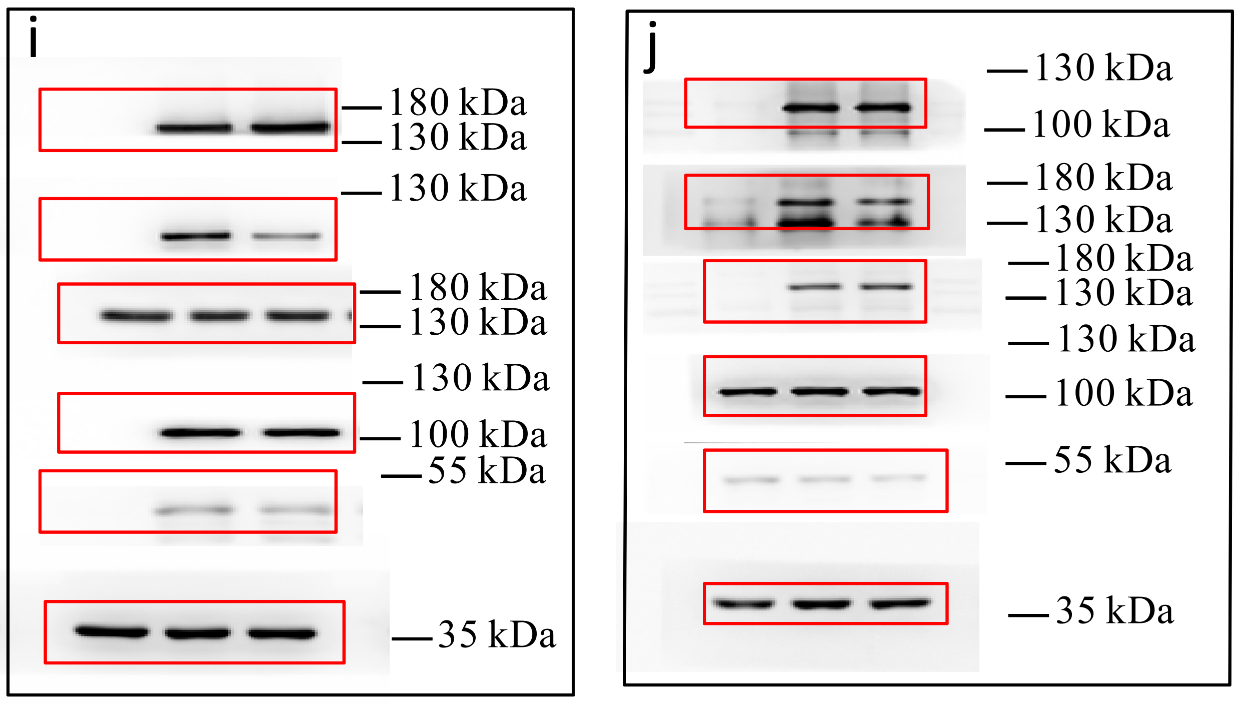

Figure7

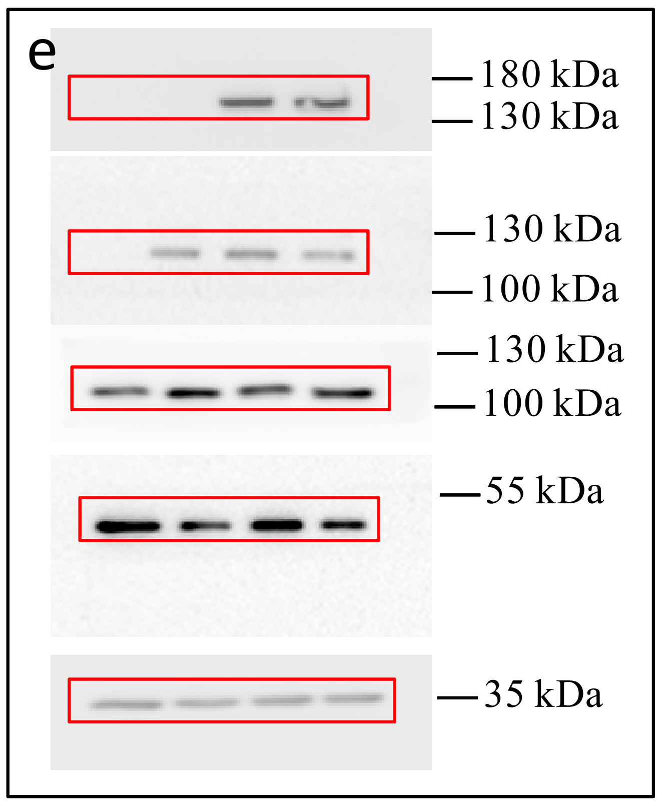

Figure S3

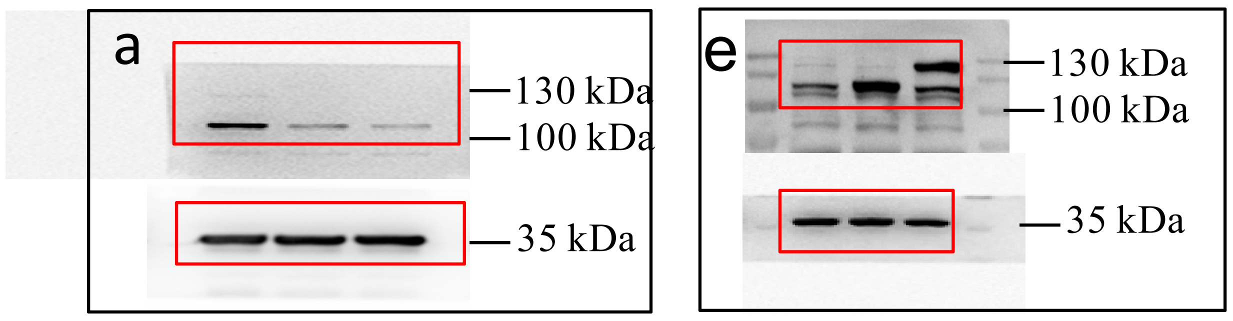

Figure S4

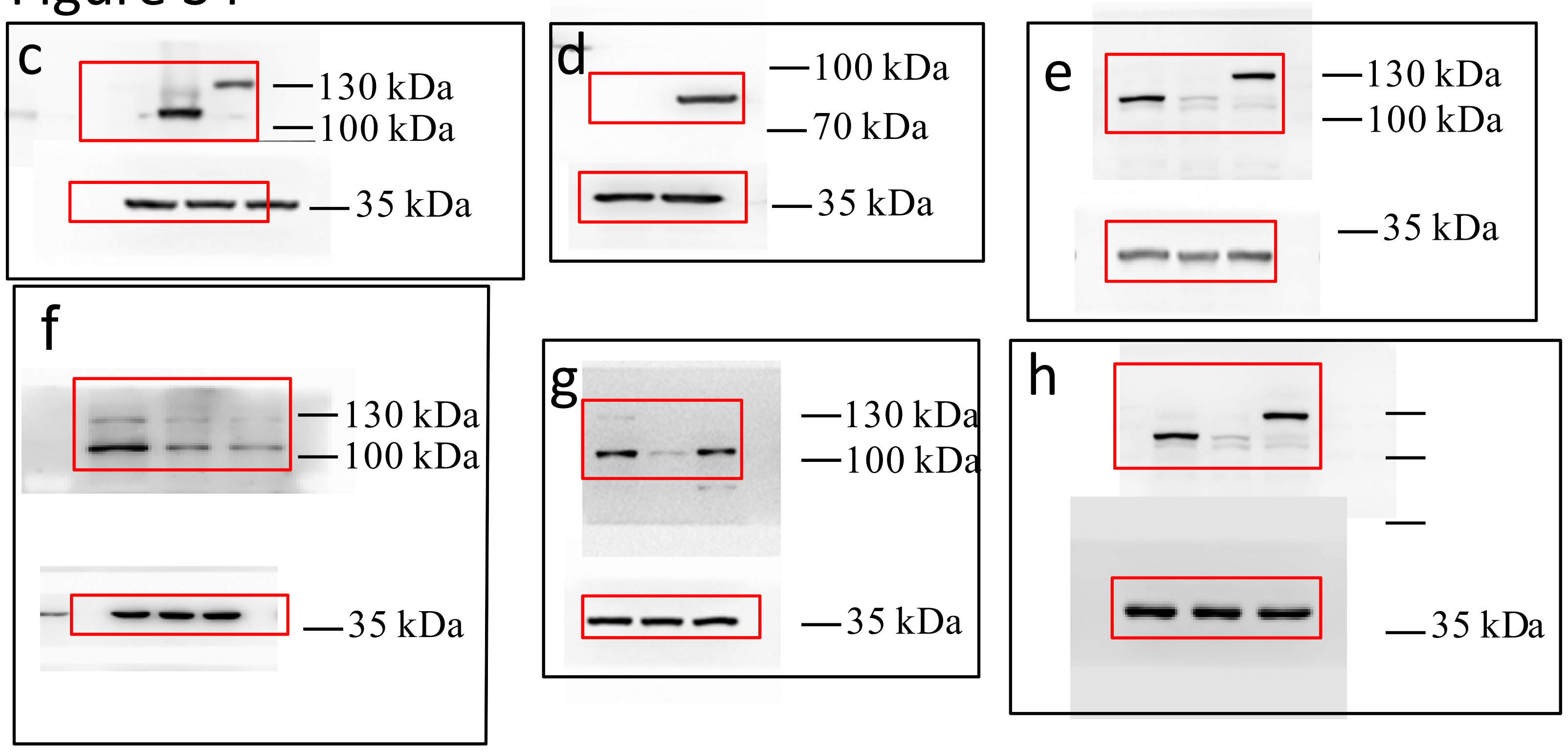

Figure S5

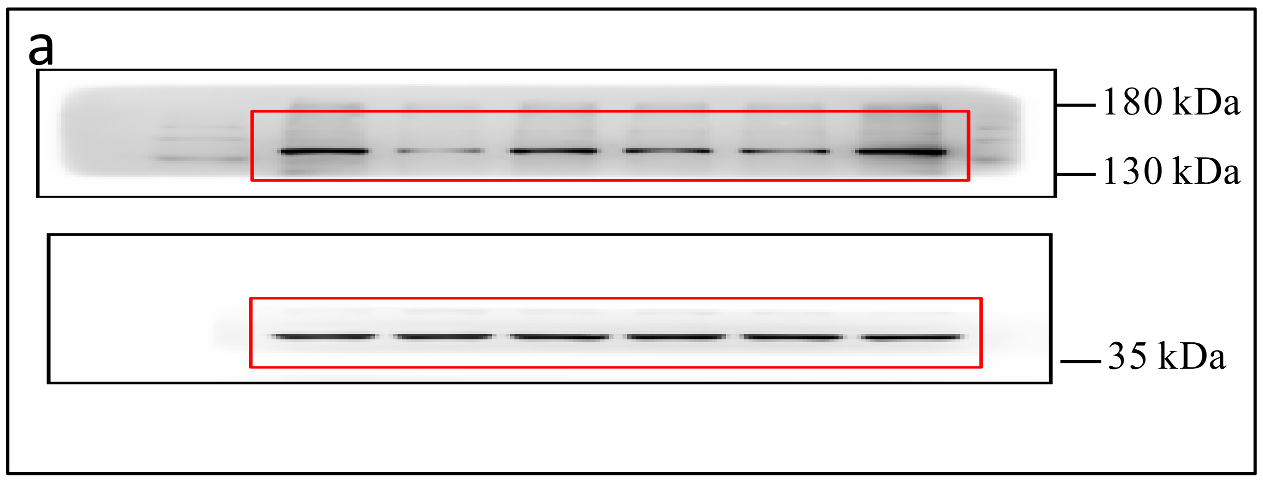

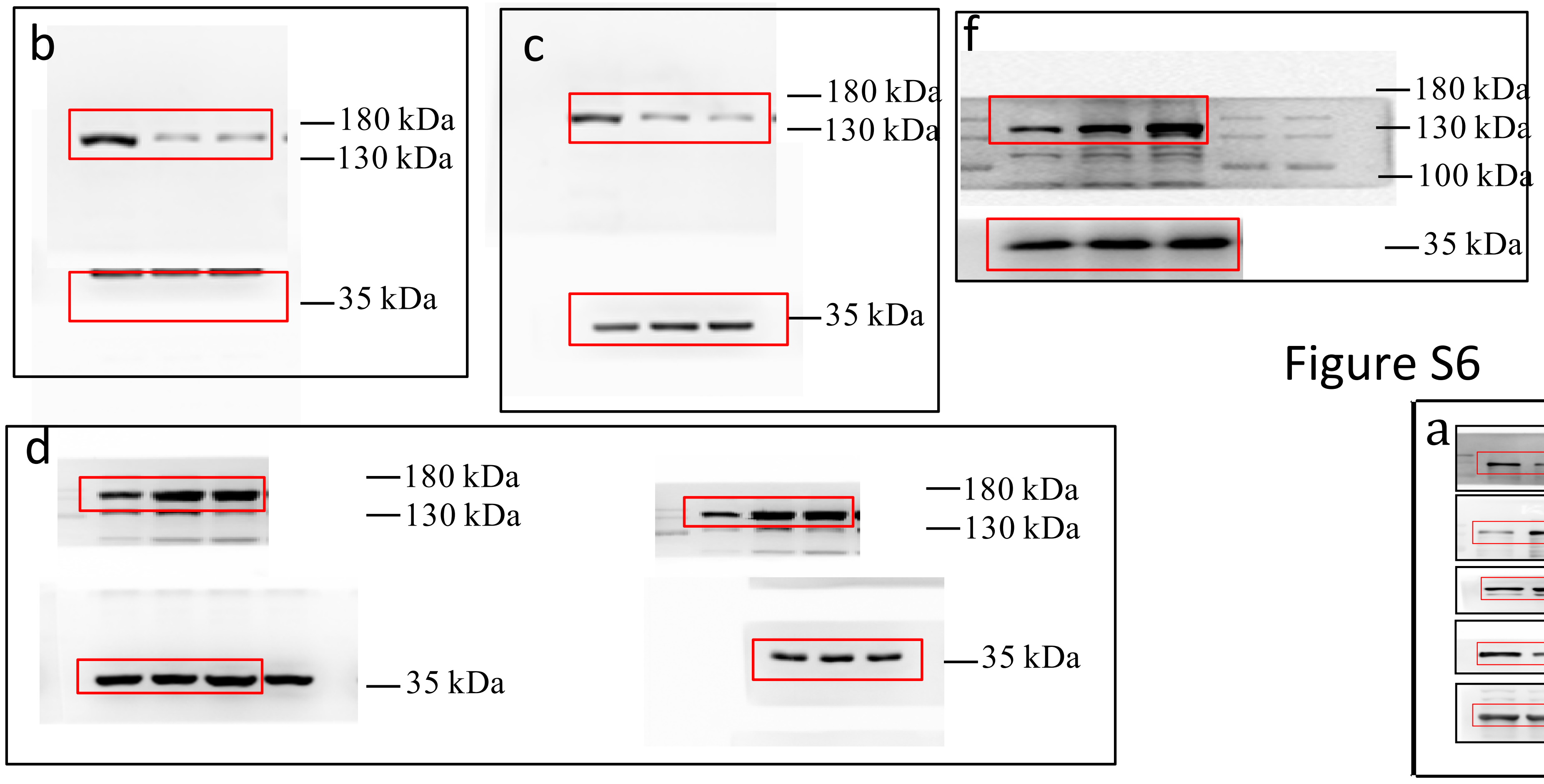

Figure S6

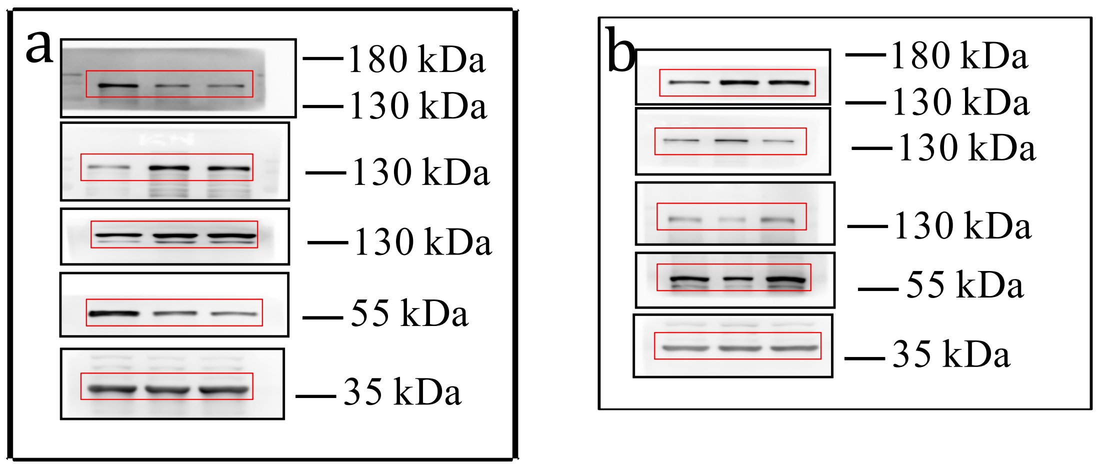

Figure S7

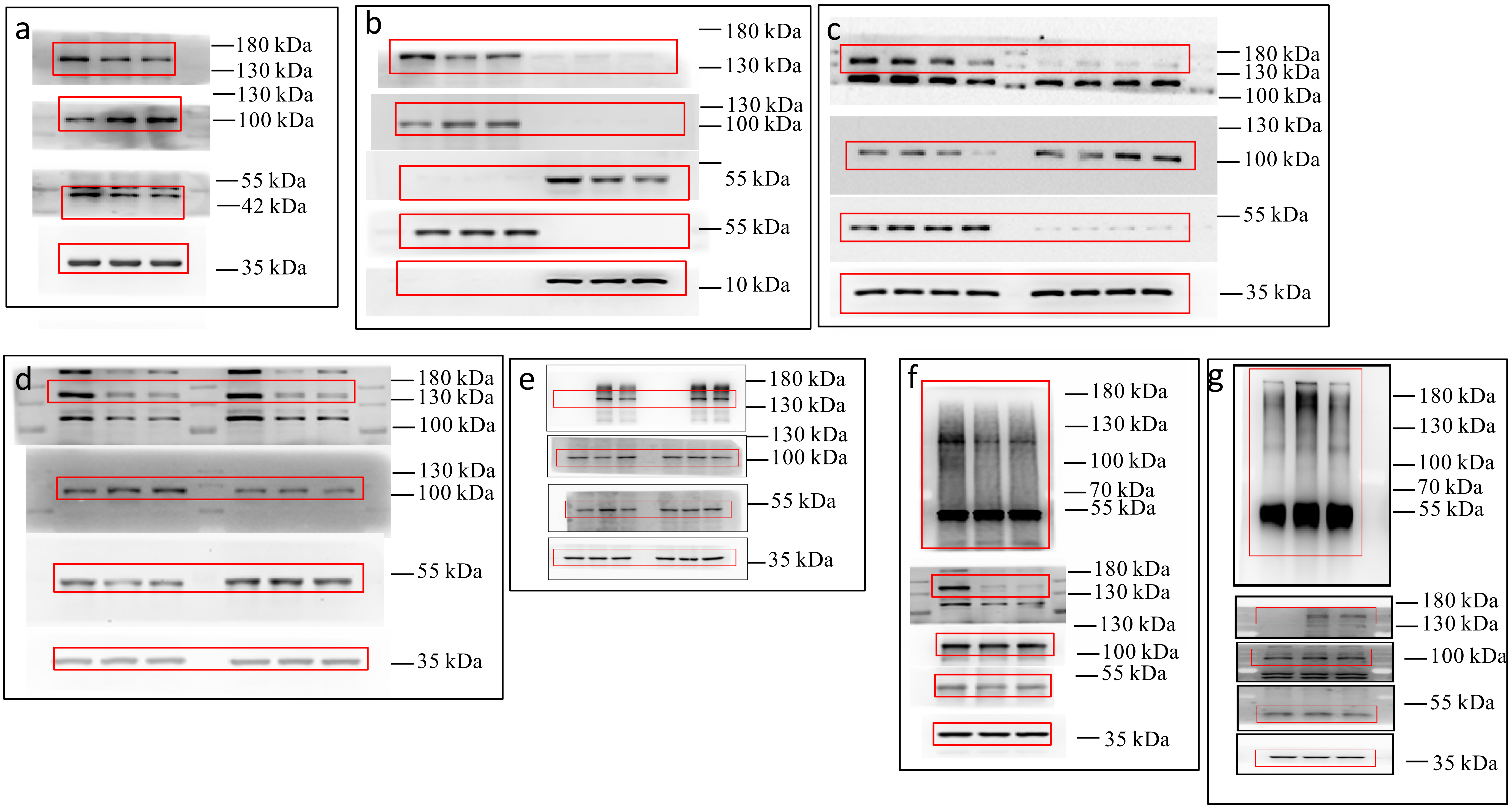

Figure S7

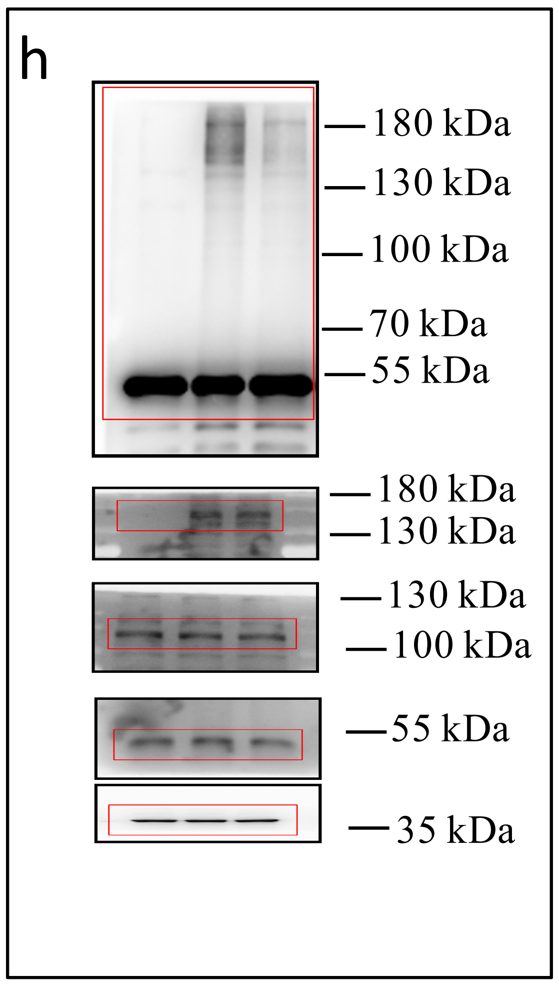

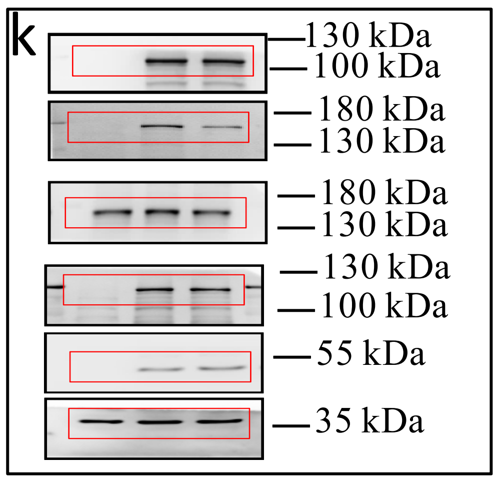

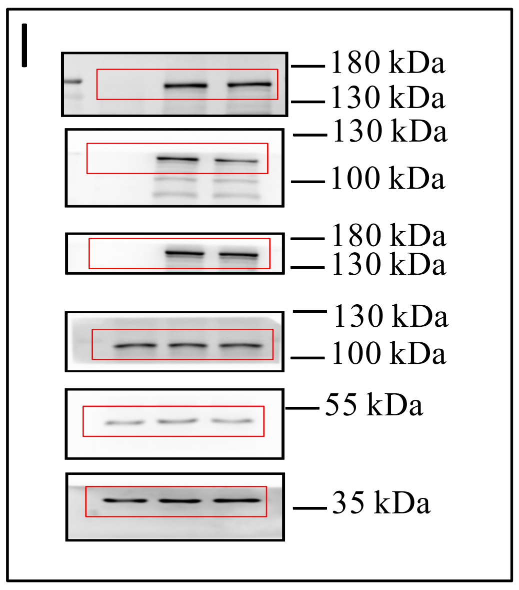

Figure S8 a

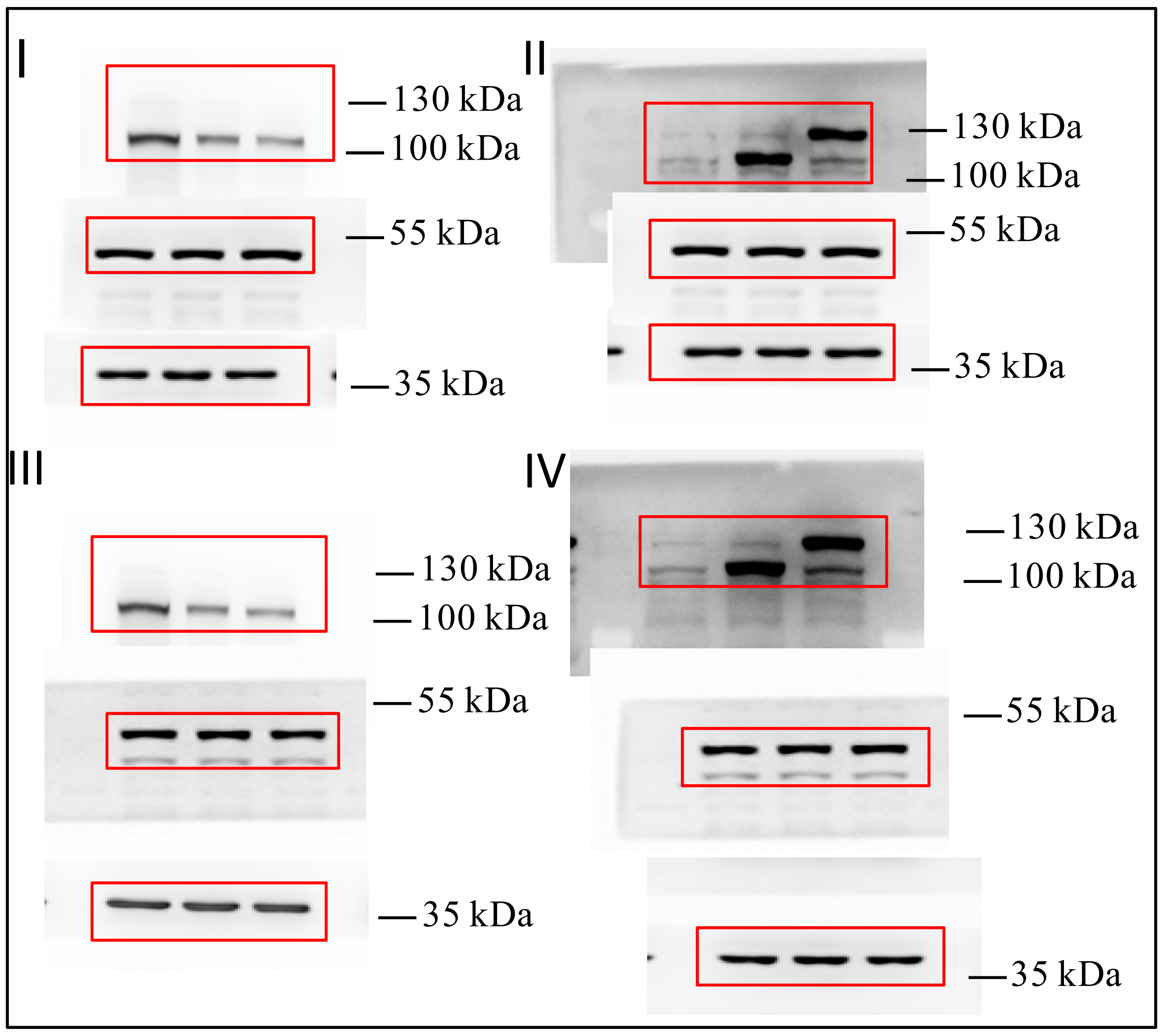

Figure S8 c

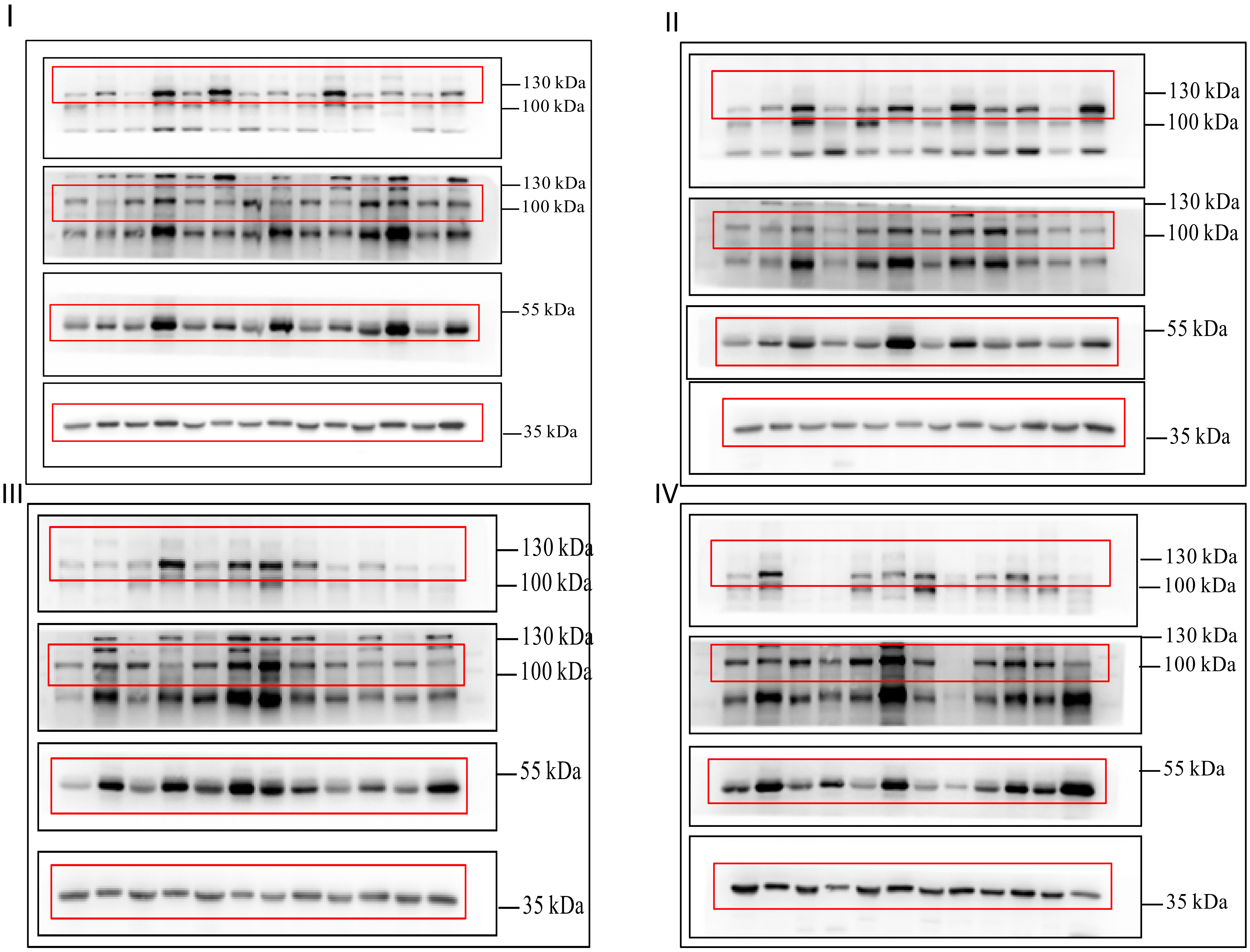

Figure S9

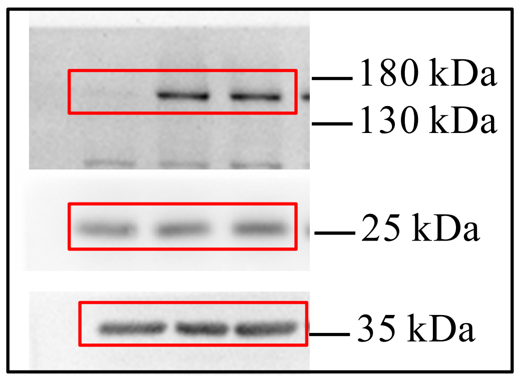

Supplement: Supplementary file 2 — Additional file 2: Supplementary file 2. [file 13046_2022_2549_MOESM2_ESM.pdf]
